# Pharmacokinetics and safety evaluation of anemoside B4 in healthy Beagle dogs

**DOI:** 10.3389/fvets.2025.1645372

**Published:** 2025-07-23

**Authors:** Jinzhao Ji, Yuqiao Ma, Shaobing Wan, Xiaoqing Ding, Jingyu Wang, Yongcheng Zhong, Yangyang Song, Junqing Zhao, Zhetong Su, Kun Jia, Shoujun Li

**Affiliations:** ^1^College of Veterinary Medicine, South China Agricultural University, Guangzhou, China; ^2^Guangdong Technological Engineering Research Center for Pet, Guangzhou, China; ^3^Guangxi Innovate Pharmaceutical Co., Ltd., Liuzhou, Guangxi, China

**Keywords:** anemoside B4, pharmacokinetics, safety, dog, veterinary medicine

## Abstract

**Background:**

Anemoside B4 (AB4), a pentacyclic triterpenoid saponin extracted from the traditional Chinese medicinal herb *Pulsatilla chinensis*, has shown anti-inflammatory and immunomodulatory effects in both preclinical and clinical studies. However, pharmacokinetic and safety data in dogs remain limited. This study aimed to evaluate the pharmacokinetics, bioavailability, and safety of AB4 in healthy Beagle dogs.

**Methods:**

In the single-dose pharmacokinetic study, 40 dogs received subcutaneous AB4 at 10, 20, or 40 mg/kg, or an intravenous bolus at 20 mg/kg. Plasma concentrations were measured using a validated HPLC–MS/MS method to determine pharmacokinetic parameters, bioavailability, dose proportionality, and sex-related differences. In the repeated-dose study, 10 dogs received 20 mg/kg subcutaneously once daily for 7 consecutive days to evaluate drug accumulation and fluctuation. In the target animal safety study, 32 dogs were randomly assigned to receive 1× (20 mg/kg), 3× (60 mg/kg), or 5× (100 mg/kg) doses of AB4, and saline as a control, via daily subcutaneous injection for 7 days. Routine clinical examinations, hematology, serum biochemistry, gross necropsy, and histopathology were assessed.

**Result:**

AB4 exhibited rapid elimination, high absolute bioavailability, and dose-proportional pharmacokinetics in the 10–40 mg/kg range. No evidence of accumulation after repeated dosing. Within the dose range of 20–100 mg/kg, AB4 demonstrated good safety, with no observable toxicity or adverse effects. No significant effects were observed on physiological parameters. Histopathological analysis revealed no consistent or target-organ specific lesions.

**Discussion:**

These findings provide fundamental pharmacokinetic and safety data to support the rational clinical use of AB4 in veterinary medicine and lay the groundwork for future clinical applications.

## 1 Introduction

*Pulsatilla chinensis* is a traditional Chinese medicinal herb that has been widely used in classical medicine for its antipyretic and detoxifying properties according to TCM theory (1, 2). In recent years, multiple triterpenoid saponins have been isolated from *P. chinensis*, among which anemoside B4 (AB4) is the most abundant (3, 4). AB4 belongs to the class of pentacyclic triterpenoid saponins (PTS) and exhibits various pharmacological effects, including immunomodulatory, anti-inflammatory, and anti-tumor activities (5–7). Owing to its low production cost and simple extraction process, AB4 has attracted increasing attention in veterinary medicine research.

Several studies have demonstrated the therapeutic potential of AB4 in inflammatory-related diseases, such as Colitis, enteritis, Acute gouty arthritis, and pneumonia (8–12). However, despite the expanding body of fundamental research, pharmacokinetic and safety data in target animal species remain scarce, particularly in dogs. Understanding the pharmacokinetic behavior and safety profile of AB4 is essential for guiding its rational clinical use, especially with regard to administration strategies, risk of drug accumulation, and the establishment of safe dosage ranges.

Pharmacokinetics, which studies the absorption, distribution, metabolism, and excretion (ADME) of drugs, plays a vital role in determining both efficacy and safety (13). As a PTS compound, AB4 is highly polar and exhibits poor membrane permeability. After oral administration, it is mainly absorbed via passive diffusion, with only a small portion absorbed actively (14, 15). AB4 has been reported to exhibit low systemic exposure and marked first-pass effects following oral administration (16). Following intravenous injection, AB4 is primarily distributed in the kidneys, followed by the lungs (17). Studies have shown that AB4 undergoes extensive hepatic metabolism, including deglycosylation, oxidation, dehydrogenation, reduction, sulfation, hydration, acetylation, and glucuronidation (18). These metabolites are mainly excreted via bile and feces, while the unmetabolized AB4 is primarily eliminated through urine (17).

In preclinical safety assessments, repeated intraperitoneal injection of AB4 at 2.5 g/kg daily for 14 days in mice did not induce adverse effects on survival, locomotor activity, or liver and kidney function (19). *In vitro* experiments also showed that AB4, at concentrations up to 200 μmol/L (approximately 244.276 μg/mL), had no significant impact on cell viability (20). Furthermore, long-term oral administration of AB4 at doses of 100 and 200 mg/kg for 38 days did not result in decreased peripheral lymphocyte counts or increased neutrophil counts in mice, nor were any obvious adverse effects or behavioral abnormalities observed, suggesting that AB4 is well tolerated under experimental conditions (21).

Although a number of preclinical studies have been conducted, further comprehensive evaluation of the pharmacokinetics and safety of AB4 in dogs is still needed. This study aims to elucidate the absorption, distribution, metabolism, and excretion of AB4 in healthy dogs, thereby providing a foundation for optimizing clinical dosing regimens. In addition, by conducting a target animal safety test, the study aims to assess the safe dosage range and potential toxicity of AB4 in dogs to ensure its safe clinical application.

## 2 Materials and methods

### 2.1 Medication and reagents

Anemoside B4 (AB4, purity: 96.4%) was purchased from the National Institutes for Food and Drug Control (Beijing, China). The investigational drug, AB4 for injection (purity: 94.2%) was provided by Guangxi Innovate Pharmaceutical Co., Ltd. (Guangxi, China). Normal saline for injection was obtained from HFQ Co., Ltd. (Jiangsu, China). All reagents and solvents used were of analytical or HPLC grade.

### 2.2 LC-MS/MS instrumentation and conditions

The HPLC-MS/MS analysis was performed using a SHIMADZU LCMS-8045 system (Shimadzu Corporation, Kyoto, Japan) equipped with a ZORBAX Eclipse Plus C18 column (2.1 × 50 mm, 1.8 μm; Agilent Technologies, USA). The mobile phase consisted of solvent A (ultrapure water) and solvent B (acetonitrile containing 0.1% formic acid). The gradient elution conditions are summarized in Table 1.

**Table 1:** Gradient elution conditions for LC-MS/MS analysis of AB4 in canine plasma.

Time (min)	Flow rate (mL/min)	Mobile phase A (%)	Mobile phase B (%)
1.00	0.30	75	25
1.50		10	90
2.50		10	90
2.60		75	25
5.50		75	25

Mass spectrometric detection was performed in MRM mode using an ESI source. The analyte AB4 was quantified using transitions m/z 1219.50 → 749.20 (quantifier, CE 55 V, Q3 pre-bias 36 V) and 1219.50 → 468.90 (qualifier, CE 41 V, Q3 pre-bias 23 V), with a Q1 pre-bias of 34 V and a dwell time of 100 ms. Instrument parameters included nebulizing gas flow 3 L/min, heating gas flow 10 L/min, interface temperature 300°C, drying gas flow 10 L/min, and desolvation temperature 526°C.

### 2.3 Method validation

The method was evaluated in terms of specificity, linearity, accuracy, precision, recovery, matrix effect, and stability, in accordance with standard bioanalytical guidelines (ICH M10, 2022).

### 2.4 Dogs

Healthy Beagle dogs (9–11 months old) purchased from Zhenhe Laboratory Animal Co., Ltd. (Fuzhou, China) were used in this study. All procedures were approved by the Animal Clinical Research Ethics Committee of the Veterinary Drug Evaluation Center, South China Agricultural University (Approval No. 2023E007). Before the study, dogs underwent a 7-day acclimation. During the study, dogs were fed a standardized diet with daily intake calculated based on resting energy requirement (RER) and maintenance energy requirement (MER) to ensure nutritional balance and appropriate caloric intake. Water was freely available.

### 2.5 Grouping and drug administration

In the single-dose pharmacokinetic (PK) study, 40 Beagle dogs (equal numbers of males and females) were randomly assigned into four parallel groups: low-dose (10 mg/kg), medium-dose (20 mg/kg), high-dose (40 mg/kg), and intravenous (20 mg/kg). AB4 was administered as a single subcutaneous injection in the dorsal neck or intravenously into the cephalic vein; In the multiple-dose PK study, 10 dogs (five males and five females) received 20 mg/kg AB4 once daily (8:00 a.m.) by subcutaneous injection for 7 consecutive days.

In the safety study, 40 dogs (equal numbers of males and females) were randomized into four groups: low-dose (20 mg/kg), medium-dose (60 mg/kg), high-dose (100 mg/kg), and a saline control group (0 mg/kg). AB4 was administered subcutaneously once daily (8:00 a.m.) for 7 days at 24-h intervals.

All AB4 solutions were prepared at a concentration of 48 mg/mL, and the administered volume was adjusted according to body weight and dosing requirements. Detailed dosing information is provided in the Supplementary materials 7–9.

### 2.6 Sample collection and processing

In the single-dose pharmacokinetic (PK) study, a blank blood sample was collected before dosing. After administration, 2 mL blood samples were collected from the cephalic vein at 5, 10, 15, 30, and 45 min, and at 1, 2, 3, 3.5, 4, 6, 9, 12, 16, 24, 36, and 48 h.

In the multiple-dose PK study, a pre-dose blood sample was collected before the first administration, followed by sampling at the same time points as the single-dose study. Additional trough samples were collected within 5 min before each daily administration. On days 2 to 6, samples were collected at 3, 3.25, 3.5, 3.75, and 4 h. After the final dose, samples were taken at the same time points as the single-dose group, with additional collections at 60 and 72 h.

Plasma was separated by centrifugation at 4000 rpm for 10 min. Subsequently, 300 μL of plasma was mixed with 900 μL of methanol, vortexed for 3 min, and centrifuged at 14,000 rpm for 10 min at 4°C. The resulting supernatant was filtered through a 0.22 μm PTFE membrane and subjected to LC–MS/MS analysis.

In the safety study, blood was collected from the cephalic vein for hematology, serum biochemistry. According to the AVMA Guidelines for the Euthanasia of Animals (2020 Edition), dogs were rapidly administered propofol intravenously at a dose of 10 mg/kg. After confirming deep anesthesia, bilateral axillary vessels were incised for exsanguination, ensuring a painless procedure. Following euthanasia, major organs were collected, weighed for organ coefficient calculation, then fixed in 10% neutral-buffered formalin, and prepared for histopathological examination using H&E staining.

### 2.7 Pharmacokinetic analysis

Plasma concentration–time data were analyzed using non-compartmental analysis (NCA) in Phoenix WinNonlin software (Certara, USA) (22). Pharmacokinetic Parameters were compared between male and female dogs within each dose group to assess sex-based differences. Bioavailability was calculated based on AUC comparison between IV bolus and SC groups. Dose linearity for key PK parameters (C_max_, AUC_0–t_, and AUC_0-∞_) was assessed using the power model approach.

### 2.8 Safety assessment procedures

Safety assessments were conducted on Days 0 (D0), 4 (D4), 7 (D7), and 14 (D14). Blood samples were collected for laboratory testing, including hematology and serum biochemistry. Local tolerance at the injection site was also monitored. Body weight was recorded on D0 and D14 (after fasting). On D14, gross anatomical examinations and organ weight measurements were performed in the control and high-dose groups. Throughout the study, dogs were monitored for clinical signs and adverse events (AEs).

### 2.9 Statistical analysis

Statistical analyses were conducted using SPSS software (version 25.0; IBM, Armonk, NY, USA), with a two-sided significance level of *α* = 0.05.

In the pharmacokinetic study, continuous variables were tested for normality and homogeneity of variance. Normally distributed data with equal variances were analyzed using independent samples t-tests; those with unequal variances were analyzed using Welch’s ANOVA. Non-normally distributed data were assessed using the Mann–Whitney U test.

In the safety study, normally distributed data are presented as mean ± SD and analyzed using one-way ANOVA, followed by Dunnett’s test or Tamhane’s T2 test depending on variance equality. Non-normally distributed data are expressed as median (P25, P75) and analyzed using the Wilcoxon rank-sum test. Categorical data are shown as frequency and percentage (*n*, %) and compared using the chi-square or Fisher’s exact test. Paired data were evaluated using the paired t-test or Wilcoxon signed-rank test.

## 3 Result

### 3.1 Method validation

The HPLC–MS/MS method established for AB4 quantification in Beagle dog plasma demonstrated acceptable selectivity, sensitivity, accuracy, and reproducibility. No significant endogenous interference or system carryover was observed. The method showed good linearity over 0.2–20 μg/mL (*R*^2^ > 0.99). Precision and accuracy were within ±15% (±20% for Lower Limit of Quantification), and recovery exceeded 80%. Dilution integrity and stability under various conditions (freeze–thaw, short−/long-term storage, post-preparative) were confirmed. Detailed validation data are provided in the Supplementary materials 1–6, 36–37.

### 3.2 Pharmacokinetics after single dose administration

At baseline, no statistically significant differences in body weight were observed among groups (*F* = 0.397, *p* = 0.756). Pharmacokinetic parameters of AB4 were analyzed using a non-compartmental model. Following single-dose administration, AB4 exhibited a short elimination half-life (*t*₁/₂ ≈ 3–4 h), a high clearance rate, and a moderate apparent volume of distribution (*V*_d_/*F* < 300 mL/kg), indicating rapid elimination and limited tissue distribution. Moreover, AB4 showed high absolute bioavailability.

Sex-based comparisons revealed statistically significant differences in terminal elimination rate constant(*λ_z_*) and t₁/₂ in the low-dose subcutaneous group: *λ_z_* was 0.23 ± 0.02 h^−1^ in females and 0.20 ± 0.02 h^−1^ in males (*t* = −2.361, *p* = 0.046), while t₁/₂ was 3.08 ± 0.22 h vs. 3.48 ± 0.22 h, respectively (*t* = 2.374, *p* = 0.045). In the intravenous group, only steady-state volume of distribution (*V*_ss_) showed a significant difference (164 ± 9 mL/kg in females vs. 207 ± 30 mL/kg in males; *F* = 8.935, *p* = 0.033). No other pharmacokinetic parameters showed statistically significant sex differences (*p* > 0.05).

**Figure 1:**
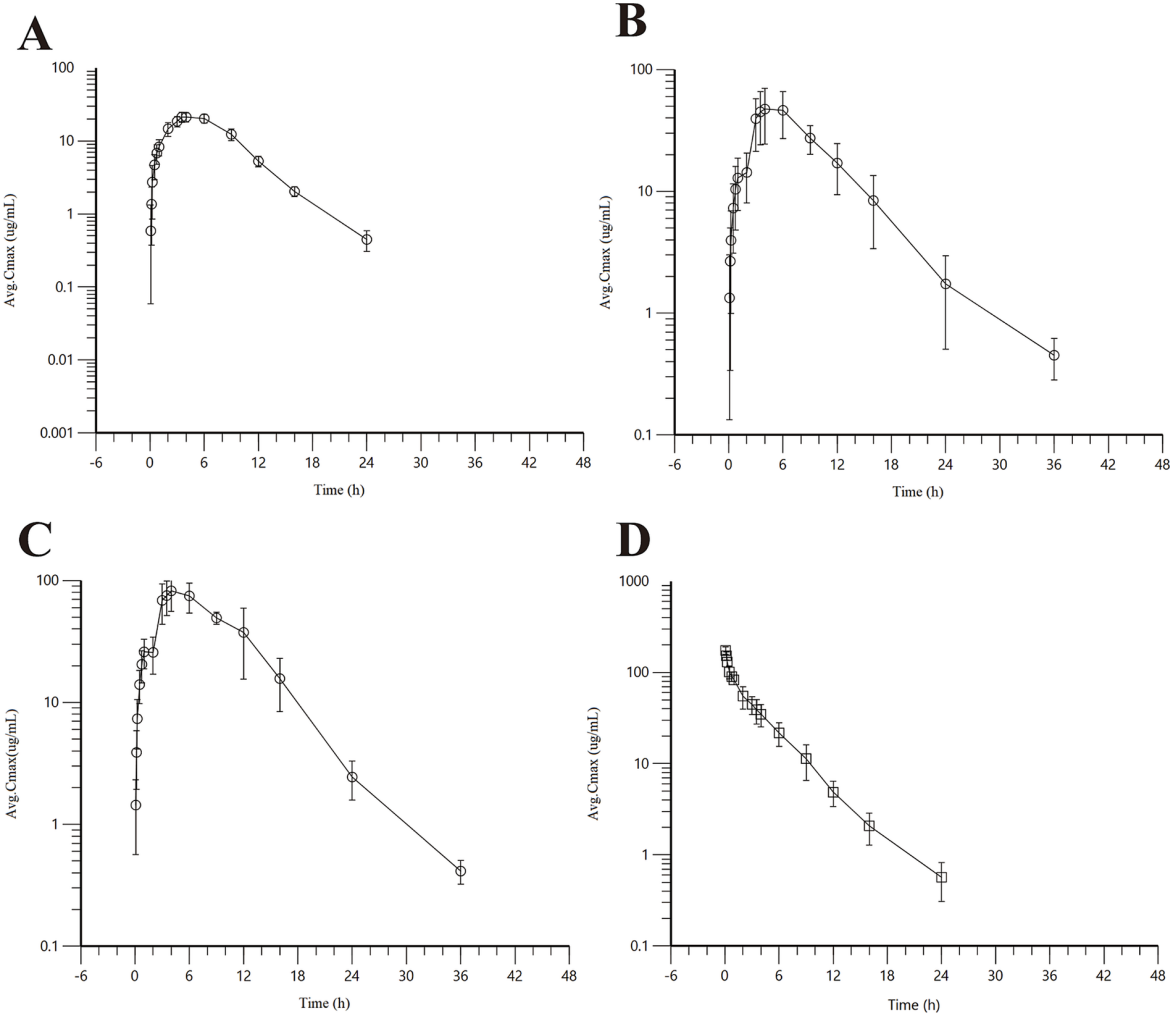
Concentration-time curve of AB4 in dogs after single dose. **(A)** Low-dose subcutaneous injection group (10 mg/kg); **(B)** midium-dose subcutaneous injection group (20 mg/kg); **(C)** High-dose subcutaneous injection group (40 mg/kg); **(D)** Intravenous bolus injection (20 mg/kg).

Detailed pharmacokinetic parameters are summarized in Table 2, and mean plasma concentration-time profiles are illustrated in Figure 1.

**Table 2:** Pharmacokinetic parameters of AB4 in canine plasma following single-dose administration ( $\bar{X}$ , *n* = 10).

Parameters	Unit	Subcutaneous injection			Intravenous bolus injection
		10 mg/kg	20 mg/kg	40 mg/kg	20 mg/kg
*C*_max_	μg/mL	22.33 ± 2.99	52.63 ± 21.27	84.28 ± 25.50	NA
*C*_0_	μg/mL	NA	NA	NA	198.30 ± 19.95
*t*_max_	h	4.00 ± 0.75	4.95 ± 1.12	4.30 ± 0.95	NA
MRT_0-t_	h	6.79 ± 0.34	8.10 ± 1.48	8.29 ± 0.62	3.74 ± 0.46
MRT_0-∞_	h	7.03 ± 0.39	8.30 ± 1.49	8.39 ± 0.60	3.91 ± 0.51
*t*_1/2_	h	3.28 ± 0.32*	3.57 ± 0.61	3.53 ± 0.50	3.34 ± 0.54
*λ_z_*	1/h	0.21 ± 0.02*	0.20 ± 0.03	0.20 ± 0.03	0.21 ± 0.04
AUC_0-t_	h·μg/mL	194.0 ± 23.3	461.8 ± 130.1	829.2 ± 239.0	427.7 ± 76.3
AUC_0-∞_	h·μg/mL	196.2 ± 23.6	465.3 ± 129.7	831.9 ± 238.6	430.7 ± 76.5
*V*_d_/F	mL/kg	245 ± 45	233 ± 59	257 ± 56	NA
CL/F	mL/h/kg	51.7 ± 6.5	46.3 ± 13.6	50.5 ± 9.9	NA
*V*_ss_	mL/kg	NA	NA	NA	185 ± 31*
CL	mL/h/kg	NA	NA	NA	48.1 ± 10.5
F	%	90.72^#^	107.97	96.94^#^	NA

“NA” indicates not applicable or statistical analysis required.

“*” indicates a statistically significant difference between females and males within the same group (*p* < 0.05).

“#” indicates that dose normalization was applied.

### 3.3 Evaluation of dose proportionality

Dose proportionality of AB4 was assessed in Beagle dogs following single subcutaneous administration at 10, 20, and 40 mg/kg. After normalization by body weight, the systemic exposure parameters(C_max_, AUC_0–t_, and AUC_0–∞_)increased approximately in proportion to the dose (1:2:4), indicating a dose-proportional relationship.

A power model analysis was performed to further evaluate linear pharmacokinetics. The estimated exponents (*β*) for C_max_, AUC_0–t_, and AUC_0–∞_ were 0.9417, 1.032, and 1.027, respectively, all of which are close to 1. These results indicated that AB4 exhibits linear pharmacokinetic behavior over 10-40 mg/kg dose range in dogs.

**Figure 2:**
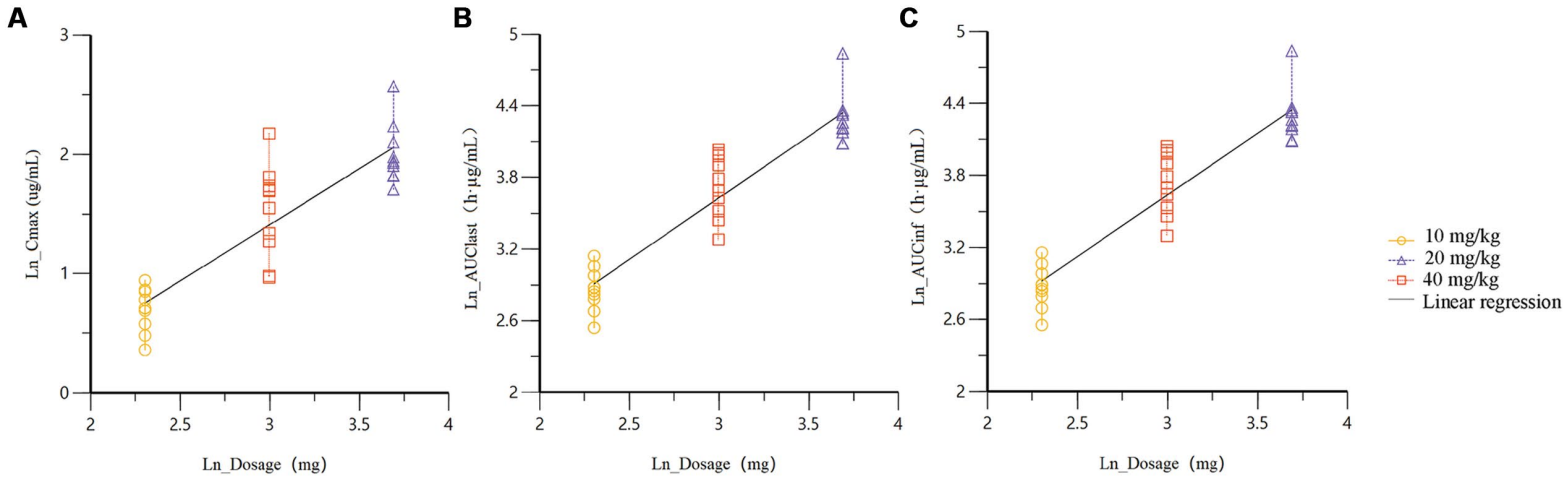
Linear regression plots of the power function model for key pharmacokinetic parameters **(A)** C_max_; **(B)** AUC_0-t_; **(C)** AUC_0-∞_.

Dose proportionality and fitted curves of systemic exposure are presented in Table 3. Linear regression plots based on the power function model for key pharmacokinetic parameters are shown in Figure 2.

**Table 3:** Dose proportionality of systemic exposure and fitted curves.

Parameters	Dose-proportionality of systemic exposure	Regression equation	Correlation coefficient (*R*^2^)
*C*_max_	1:2.36:3.77	*C*_max_ = 0.2429·Dose^0.9417^	*R*^2^ = 0.7832
AUC_0-t_	1:2.41:4.22	AUC_0-t_ = 1.706·Dose^1.032^	*R*^2^ = 0.8760
AUC_0-∞_	1:2.41:4.19	AUC_0-∞_ = 1.747·Dose^1.027^	*R*^2^ = 0.8764

### 3.4 Pharmacokinetics after multiple dose administration

Following repeated subcutaneous administration of AB4 at 20 mg/kg once daily for 7 days, the mean steady-state trough concentration (C_ss-min_) was 1.35 ± 0.51 μg/mL, and the mean steady-state average concentration (C_ss-avg_) was 19.61 ± 5.27 μg/mL. The degree of fluctuation (DF) was 2.52 ± 0.42.

**Figure 3:**
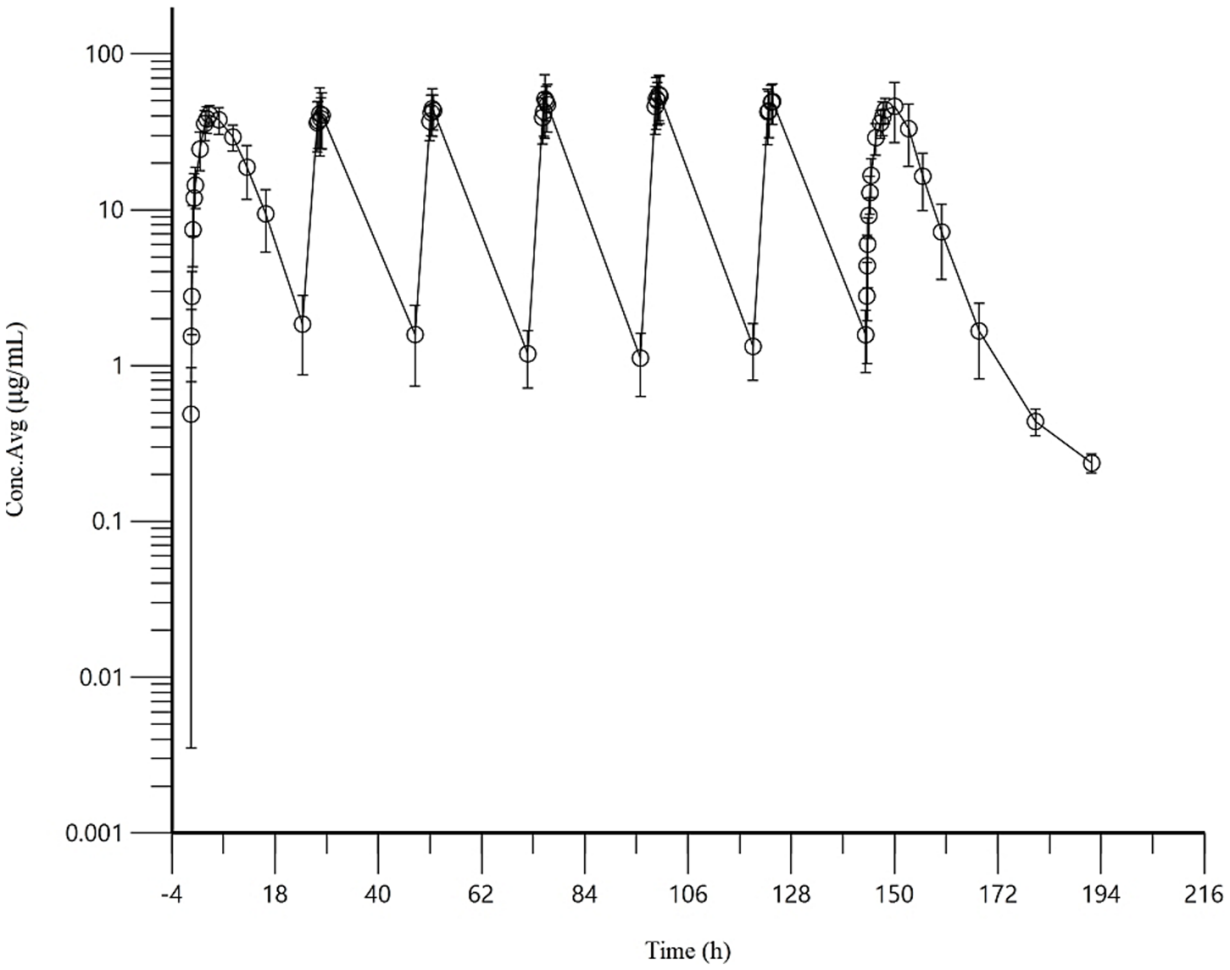
Concentration-time curve of AB4 in dogs after multiple doses (20 mg/kg). “NA” indicates not applicable or statistical analysis required.

The accumulation ratios estimated by three different methods were as follows: R (based on the terminal elimination rate constant) was 1.03 ± 0.01, R_AUC_ was 1.22 ± 0.52, and R_Cmax_ was 1.16 ± 0.18. These results indicate that AB4 exhibits no significant accumulation in dogs under the tested dosing regimen. The concentration-time curve of AB4 in dogs after multiple dosing is presented in Figure 3, and pharmacokinetic parameters following repeated subcutaneous administration (*n* = 10) are summarized in Table 4.

**Table 4:** Pharmacokinetic parameters of AB4 in dogs following multiple dose administration ( $\bar{X}$ , *n* = 10).

Parameters	Unit	Dosing Schedule	
		Initial administration	Continuous administration for 7 days
*λ_z_*	h^−1^	0.17 ± 0.05	0.16 ± 0.03
*t*_1/2_	h	4.29 ± 1.07	4.39 ± 0.76
*t*_max_	h	4.3 ± 0.92	4.8 ± 1.74
*C*_ss-min_	μg/mL	NA	1.35 ± 0.51
*C*_ss-avg_	μg/mL	NA	19.61 ± 5.27
*C*_ss-max_	μg/mL	NA	51.05 ± 18.61
*C*_max_	μg/mL	42.66 ± 7.01	NA
AUC_0-t_	h·μg/mL	403.2 ± 82.6	482.0 ± 133.8
AUC_0-∞_	h·μg/mL	466.8 ± 119.0	485.1 ± 133.0
AUC_ss_	h·μg/mL	NA	470.6 ± 126.5
*V*_d_/F	mL/kg	267 ± 35	NA
*V*_ss_/F	mL/kg	NA	280 ± 58
CL/F	mL/h/kg	45.4 ± 11.7	NA
CL_ss_/F	mL/h/kg	NA	45.6 ± 23.1
MRT_0-∞_	h	9.02 ± 1.36	8.21 ± 0.89
*R_λz_*		NA	1.03 ± 0.01
*R*_AUC_		NA	1.22 ± 0.52
*R*_*C*max_		NA	1.16 ± 0.18
*DF*		NA	2.52 ± 0.42

“NA” indicates not applicable or statistical analysis required.

### 3.5 Clinical observations

At baseline, body weight in the 5 × dose group was significantly lower than the saline control group (*p* = 0.008), while no significant differences were observed in other treatment groups (*p* > 0.05). During the entire observation period (Days 0–14), Dogs in treatment and control groups maintained stable vital signs. Body temperature, respiratory rate, and heart rate remained within normal physiological ranges, with no signs of fever, hypothermia, tachypnea, or arrhythmia. Food and water intake were normal, with no cases of anorexia or polydipsia. Fecal and urinary characteristics appeared normal. Dogs were alert and active, with bright eyes and responsive behavior. Mucous membranes were moist and pink, and the skin on the lips and nose remained normal. No injection site reactions (e.g., erythema, edema, pain, or pruritus) were observed, apart from mild bleeding due to needle puncture. No significant differences in body weight gain were observed between groups (*p* > 0.05), indicating no treatment-related effects. No adverse events or mortality occurred during the study. Injection site manifestations are provided in the Supplementary material 38.

### 3.6 Blood hematology and serum biochemistry

Hematological results showed that during the administration period, red blood cell parameters, platelet counts, and white blood cell counts in dogs remained within normal ranges, with no statistically significant differences compared to the saline control group. On Day 14 (seven days after drug withdrawal), statistically significant differences in neutrophil (NEUT) and total white blood cell (WBC) counts were observed between the treatment groups and the control group.

Serum biochemistry results indicated that serum markers of liver function (AST, ALT, ALP, GGT, and TBIL) remained within reference ranges, with no statistically significant differences from the control group on any test day. Renal function parameters (CREA and UREA) showed minor statistical differences at isolated time points, however, all values remained within physiological ranges and did not persist throughout the study. Indicators of protein metabolism (TP, ALB, GLOB) and lipid/glucose metabolism (TG, GLU, TC) were also within reference ranges. Although some intergroup differences were observed at certain time points, no trends related to drug intervention were identified, and the variations were primarily attributed to individual differences among animals. No significant effects were observed on muscle function (CK) or on calcium and phosphorus levels.

These findings indicated that AB4 administered at doses of 20–100 mg/kg did not induce clinically significant effects on hematological or serum biochemical profiles in dogs. All data are detailed in Supplementary materials 12–35.

### 3.7 Necropsy and histopathological examination

Gross necropsy findings in the 5 × dose group revealed no visible abnormalities in the heart, liver, spleen, lungs, kidneys, thymus, pancreas, gastrointestinal tract, and lymph nodes. All examined organs exhibited normal color, intact serosal surfaces, and well-defined margins, with no signs of hemorrhage, ulceration, necrosis, or other gross lesions. No apparent differences were noted compared to the saline control group.

Organ coefficient analysis showed no statistically significant differences (*p* > 0.05) in the weights of major organs (heart, liver, spleen, lungs, and kidneys) between the 5 × dose group and the saline control group. These results indicated that AB4 administration did not cause hypertrophy, atrophy, or abnormal enlargement of the major organs. Detailed results are presented in Table 5.

**Table 5:** Results of organ coefficient measurement (Unit: /, $\bar{X}$ ±SD, *n* = 8).

Group	Organ name				
	Heart	Liver	Spleen	Lungs	Kidneys
5 × dose group	0.84 ± 0.13	3.09 ± 0.30	0.26 ± 0.02	0.91 ± 0.13	0.52 ± 0.08
Saline control group	0.94 ± 0.04	2.81 ± 0.29	0.30 ± 0.05	0.94 ± 0.13	0.48 ± 0.07
*t*	−2.08	1.902	−1.983	−0.468	0.975
*p* value	0.069	0.078	0.067	0.647	0.346

**Figure 4:**
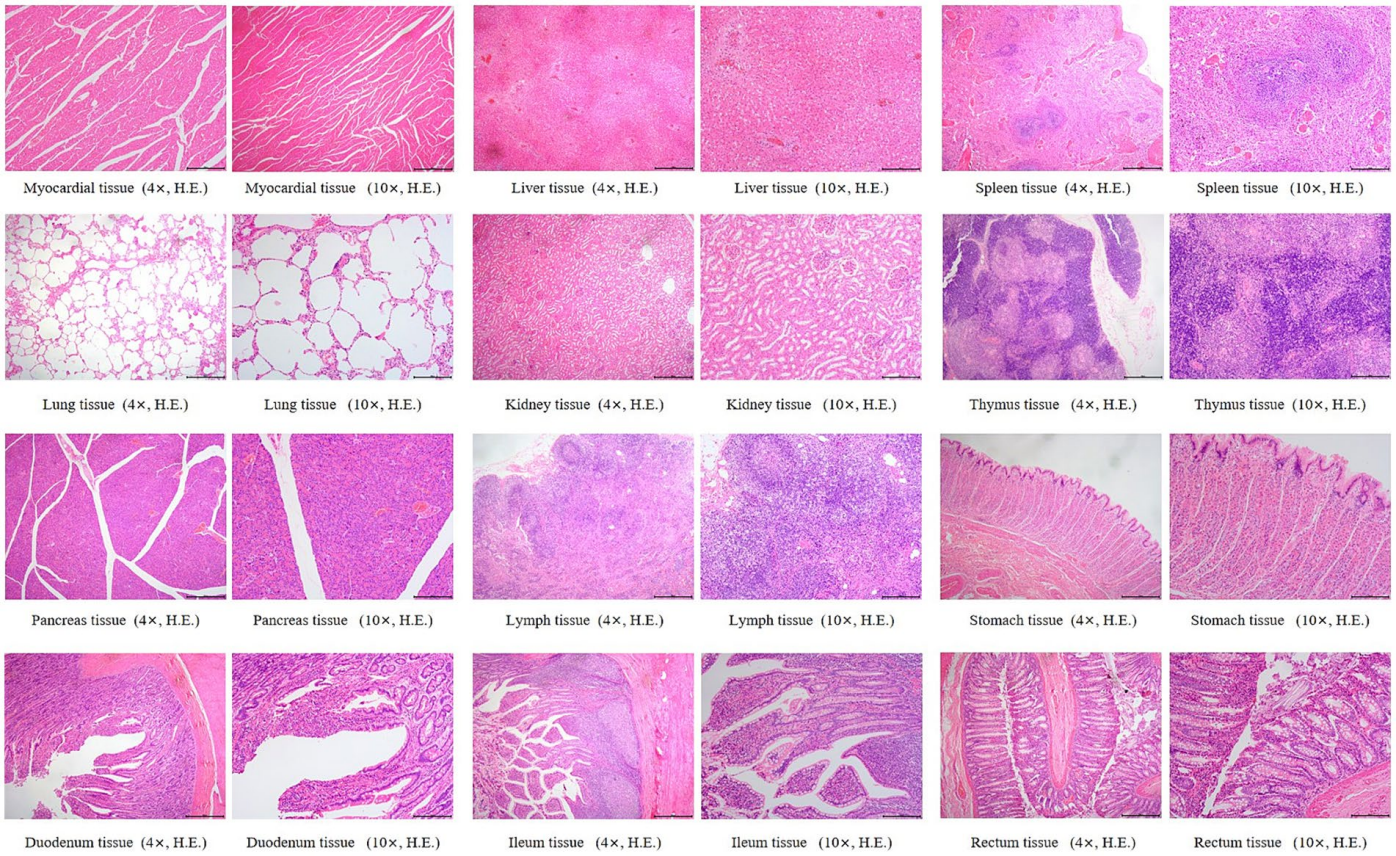
Histopathological sections of dogs in the 5 × dose group. “4×”: Microscopic images were captured using a 4 × objective lens, scale bar = 500 μm; “10×”: Microscopic images were captured using a 10 × objective lens, scale bar = 200 μm.

**Figure 5:**
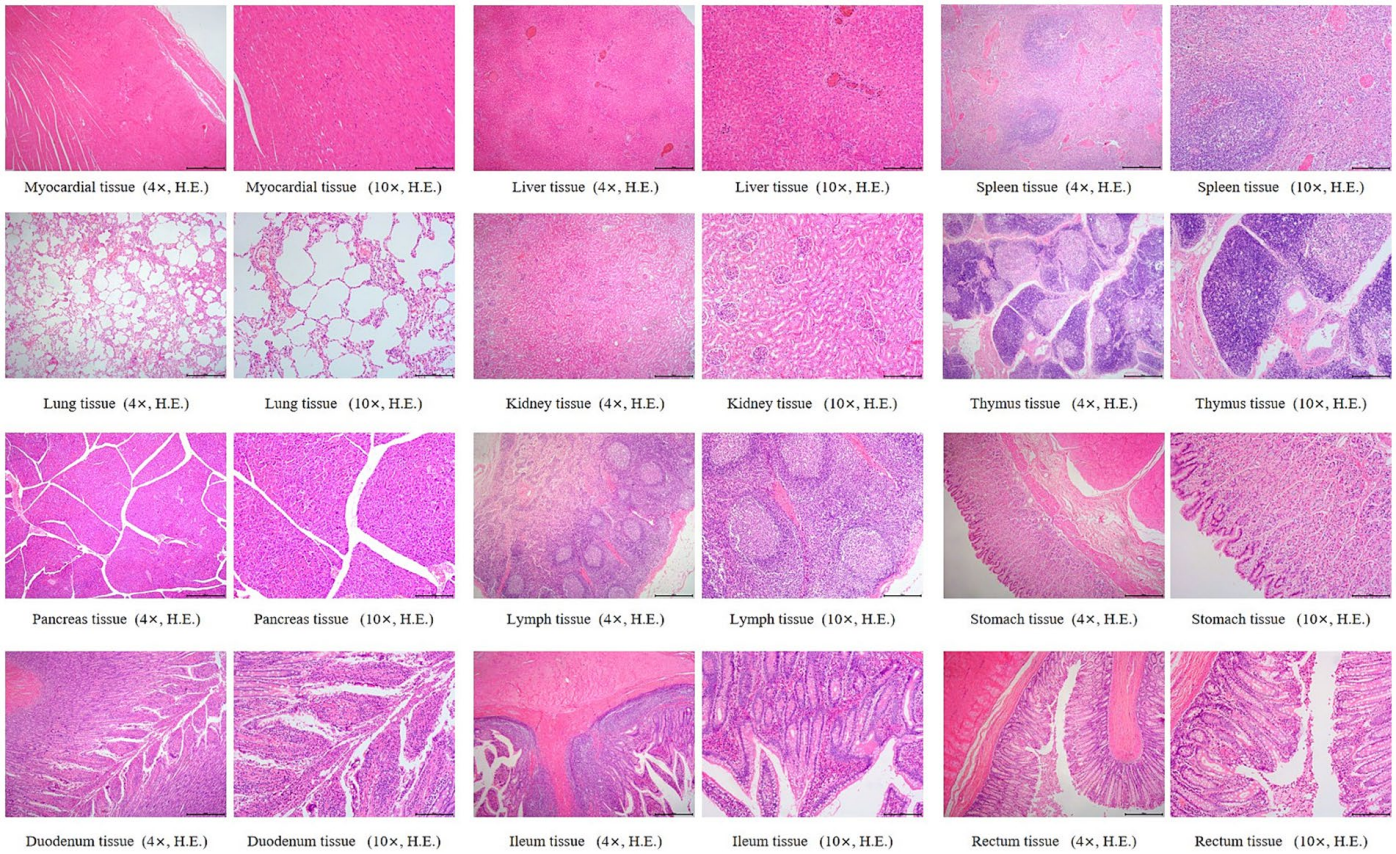
Histopathological sections of dogs in the saline control group. “4×”: Microscopic images were captured using a 4 × objective lens, scale bar = 500 μm; “10×”: Microscopic images were captured using a 10 × objective lens, scale bar = 200 μm.

Histopathological changes observed in the 5 × dose group, diffuse hepatic vacuolar degeneration, lymphoid follicle hyperplasia in the renal cortex (1/8, 12.5%), splenic nodule edema with loosely arranged lymphocytes, intestinal villus edema, lymph node edema (1/8, 12.5%), and congestion of hepatic vessels and sinusoids (1/8, 12.5%) were observed. In the saline control group, focal pulmonary interstitial hyperplasia (1/8, 12.5%), decreased lymphocytes in the splenic red pulp, proteinaceous exudate in renal tubules accompanied by epithelial necrosis, and pancreatic edema (1/8, 12.5%) were noted. These histopathological findings did not exhibit consistency or directional trends and were determined to be incidental or attributable to individual variation. There was no statistically significant difference in the incidence of lesions between the two groups (*p* > 0.05). Detailed results are presented in Table 6. Representative histopathological sections are shown in Figures 4, 5, and additional sections are provided in the Supplementary material 39.

**Table 6:** Incidence of tissue lesions in dogs.

Group	Dogs exhibiting tissue lesions		*Z*	*p* value
	Frequency count (*n* = 8)	Frequency (%)		
5 × dose group	3	37.5	−0.522	0.602
Saline control group	2	25.0		

## 4 Discussion

To date, most publicly available pharmacokinetic data on AB4 have been obtained from rodent models, with a lack of corresponding PK and safety data in dogs (23–25). This study presents a comprehensive and detailed characterization of the pharmacokinetic profile of AB4 in healthy dogs following both single and repeated subcutaneous administration, and systematically evaluates its safety. The findings provide a predictive framework for developing rational dosing strategies in clinical veterinary settings.

Following a single subcutaneous injection, AB4 exhibited limited distribution, rapid elimination, and high bioavailability, with linear pharmacokinetics. At the low dose level (10 mg/kg), statistically significant differences in *λ_z_* and *t*_1/2_ were observed between male and female dogs (*p* < 0.05), whereas no such differences were noted at the medium and high dose levels. This phenomenon may be attributed to AB4’s strong plasma protein binding capacity (binding rate >80%) (26). It has been reported that *α*₁-acid glycoprotein (AAG), a major binding protein for basic drugs, is typically more abundant in males (27). At lower plasma concentrations, variations in protein binding can substantially affect the free drug fraction. As the administered dose increases, binding sites become saturated, diminishing the influence of plasma proteins and thereby reducing sex-related difference. In this study, although *λ_z_* and *t*_1/2_ (*p* = 0.045) in the low-dose group were statistically significant, their *p*-values approached the 0.05 threshold. To further assess the robustness of these findings, the Mann–Whitney U test-a nonparametric test with broader applicability-was employed, revealing no significant sex differences for *λ_z_* (*U* = 21, *p* = 0.76) or *t*_1/2_ (*U* = 3.5, *p* = 0.59). Given that no sex differences were observed in other PK parameters or in the medium- and high-dose groups, these results are considered marginally significant and of limited clinical relevance.

Under repeated subcutaneous administration at 20 mg/kg for seven consecutive days, AB4 demonstrated an accumulation ratio <2 and a degree of fluctuation between 1 and 3, indicating no significant accumulation and moderate plasma concentration variability. Although repeated administration led to a slight increase in *t*_1/2_ and volume of distribution compared to single dosing, the differences were not statistically significant, suggesting that AB4 maintains a stable pharmacokinetic profile upon multiple dosing. However, the mean peak-to-trough concentration ratio at steady state was relatively high. Based on these pharmacokinetic findings, it is recommended that clinical dosing strategies be adjusted accordingly: For acute or severe cases, an increased initial dose or more frequent dosing may be warranted to rapidly achieve therapeutic plasma levels. Conversely, for milder cases or during recovery, a reduced dose with increased dosing frequency may help lower drug burden and improve treatment precision.

In the safety study, a high dose of 100 mg/kg was employed without inducing abnormalities in liver or kidney function or histopathological alterations. Combined with the PK findings, which indicate rapid elimination and no evidence of drug accumulation after 7 days of administration, the data further support the safety of AB4. Throughout the study period, no antibiotics were administered. Although subcutaneous injections and blood sampling could induce minor inflammatory responses, NEUT and WBC levels in the treatment groups remained within normal limits and exhibited minimal fluctuation, which may reflect the anti-inflammatory properties of AB4 (28, 29). In contrast, the saline control group exhibited greater variability in NEUT and WBC levels, with values significantly higher on Day 14 compared to some treatment groups.

Body weight in all groups showed a significant increase on Day 14 compared with baseline (Day 0), and the extent of weight gain was consistent with the expected growth pattern for Beagle dogs of this age, with no evidence of drug-induced weight loss or growth suppression, further supporting the favorable tolerability of AB4. Detailed results are provided in the Supplementary material 10.

In current veterinary practice, treatment of inflammatory diseases such as pneumonia commonly relies on glucocorticoids, which can lead to adverse effects including immunosuppression, gastrointestinal bleeding, and osteoporosis when used long term (30, 31). Compared with glucocorticoids, AB4 appears to offer a more favorable safety profile, making it a promising candidate for long-term anti-inflammatory therapy.

Future studies should focus on elucidating the tissue distribution and metabolic pathways of AB4 in target organs, particularly the lungs and kidneys. These investigations will provide critical insights into its therapeutic mechanisms and the potential risk of organ-specific accumulation. Additionally, further pharmacodynamic (PD) studies are warranted to establish a comprehensive PK/PD model. This model would enable more precise characterization of AB4’s *in vivo* disposition and therapeutic effects, facilitating accurate predictions of drug concentrations and efficacy under various dosing regimens. Collectively, these studies will inform optimized treatment strategies and reinforce the scientific foundation for AB4’s future application in veterinary medicine.

## 5 Conclusion

This study provides a detailed pharmacokinetic and safety evaluation of AB4 in dogs. Following subcutaneous administration, AB4 exhibited limited distribution, rapid elimination, high absolute bioavailability, and linear pharmacokinetic characteristics, with no evident accumulation after repeated dosing. Within the dose range of 20–100 mg/kg, AB4 demonstrated good safety, with no observable toxicity or adverse effects. These pharmacokinetic and safety data may inform future veterinary applications and contribute to rational dose optimization in subsequent clinical studies.

## Data Availability

The original contributions presented in the study are included in the article/Supplementary material, further inquiries can be directed to the corresponding author.
