# Supplementary material for: Pharmacokinetics and safety evaluation of anemoside B4 in healthy Beagle dogs
Source: Front Vet Sci. 2025 Jul 23;12:1645372. doi: 10.3389/fvets.2025.1645372 (PMC12327295; doi:10.3389/fvets.2025.1645372)
Supplement: Supplementary file 1 [file Data_Sheet_1.pdf]

Supplementary Material

1 Supplementary Table 1- S/N at Different Concentrations

	Concentration(μg/mL)	Signal-to-noise ratio(S/N)					Average
LOD	0.16	200.9	347.58	443.25	456.45	604.35	432.83
LLOQ	0.20	660.96	711.72	783.64	569.13	1098.79	749.78

2 Supplementary Table 2- Regression Equations of Calibration Curves and Coefficients of Determination (R^2)

Batch	Linear range(μg/mL)	Regression equation of the calibration curve	R^2
1	0.2～20	S= 330711C + 2835.38	0.9903
2		S= 361502C +520.945	0.9949
3		S= 359662C +5405.48	0.9947

3 Supplementary Table 3- Accuracy and Precision of AB4 Determination in Canine Plasma($\overline{X}$, n=6)

Nominal concentration (μg/mL)	Batch	Intra-batch accuracy(RE, %)	Intra-batch precision(RSD, %)	Inter-batch accuracy(RE, %)	Inter-batch precision(RSD, %)
0.2	1	-17.03	10.99	-15.55	7.78
	2	-18.41	4.17		
	3	-11.22	4.68		
0.6	1	-6.26	7.64	-7.92	7.77
	2	-14.22	3.73		

	3	-3.30	6.21		
	1	-2.48	5.39		
2.0	2	-11.81	1.71	-4.76	7.61
	3	-0.01	7.46		
	1	-7.30	4.94		
15.0	2	-14.60	1.92	-11.55	6.77
	3	-12.76	9.13		

4 Supplementary Table 4- Extraction Recovery of AB4 in Canine Plasma($\overline{X}$, n=6)

Nominal concentration (μg/mL)	Batch	Recovery (%)	Standard deviation (SD, %)	Coefficient of variation (CV, %)
	1	82.98	-17.03	10.99
0.2	2	81.59	-18.41	4.17
	3	88.78	-11.22	4.68
	1	93.74	-6.26	7.64
0.6	2	85.78	-14.22	3.73
	3	96.70	-3.30	6.21
	1	97.52	-2.48	5.39
2.0	2	88.19	-11.81	1.71
	3	100.00	-0.01	7.46

	1	92.70	-7.30	4.94
15.0	2	85.40	-14.60	1.92
	3	87.24	-12.76	9.13

5 Supplementary Table 5- Dilution Reliability Assessment ($\overline{X}$, n=6)

Dilution factor	AB4(μg/mL)		Accuracy deviation (RE, %)	Precision (RSD, %)
	Nominal concentration	Measured concentration		
5×	10.0	11.17	11.66	5.84
10×	5.0	5.70	14.08	5.13

6 Supplementary Table 6- Stability Assessment ($\overline{X}$, n=6)

Sample type	Evaluated conditions	AB4(μg/mL)		Accuracy deviation (RE, %)	Precision (RSD, %)
		Nominal concentration	Measured concentration		
Stock solution	-20 ℃ (30 d)	1.0	1.05	4.85	1.92
	-20 ℃ (60 d)	1.0	1.08	7.66	1.40
Whole blood	Left at room temperature (2 h)	0.6	0.60	0.17	7.02
		15.0	13.34	-11.06	7.59
Biological matrix	Long-term storage at − 20 °C (30 d)	0.6	0.65	1.40	7.10
		15.0	14.41	-3.92	6.11

	Long-term storage at -20 °C (60 d)	0.6	0.69	7.06	6.50
		15.0	14.17	-5.50	4.54
	Three freeze-thaw cycles	0.6	0.55	-8.72	6.92
		15.0	12.91	-13.94	4.65
Sample extract to be analyzed	Left at room temperature (24 h)	0.6	0.55	-8.31	6.29
		15.0	13.24	-11.71	3.86
	Placed in the autosampler tray(24 h)	0.6	0.67	11.85	8.89
		15.0	15.83	5.53	8.17

7 Supplementary Table 7- Body Weight and Dosing Information of Dogs in Single-Dose Administration Trial

Group	Number (n)	Body weight (kg)	F	*P* value	Dose (mg/kg)	Route of administration
Low-dose subcutaneous injection group	10	11.06±0.73			10	Subcutaneous injection, single dose
Medium-dose subcutaneous injection group	10	10.75±0.91	0.397	0.756	20	Subcutaneous injection, single dose
High-dose subcutaneous injection group	10	11.06±0.72			40	Subcutaneous injection, single dose
Intravenous bolus injection group	10	10.76±1.12			20	Intravenous injection, single dose

1. Body weight between groups was Analyzed by one-way ANOVA.

2. The dosing regimen in this study was based on prior dose-ranging experiments identifying 20 mg/kg as an effective dose. This was further validated in a Phase III clinical study. To assess dose proportionality, 10 mg/kg and 40 mg/kg dose groups were included in the single-dose pharmacokinetic study.

8 Supplementary Table 8- Body Weight and Dosing Information of Dogs in Repeated-Dose Administration Trial

Group	Number (n)	Body weight (kg)	Dose (mg/kg)	Route of administration
Multiple-dose administration group	10	10.84±0.48	20	Subcutaneous injection, once daily for 7 consecutive days

9 Supplementary Table 9- Body Weight and Dosing in safety trail

Group	Number (n)	Body weight (kg)	Dose (mg/kg)
5× dose group	8	9.84±0.61	100
3× dose group	8	9.86±0.83	60
1× dose group	8	10.63±0.65	20
Normal saline control group	8	10.61±0.38	0

1. To evaluate the safety margin, 60 mg/kg (3×) and 100 mg/kg (5×) dose groups were selected based on the effective dose of 20 mg/kg established in prior experiments and validated in a Phase III clinical study.

10 Supplementary Table 10- Body Weight and Weight Gain of Experimental Dogs(Unit: kg, $\overline{X}$±SD, n=8)

Group	Assessment day		Weight gain	Feed intake
	D0	D14		
5× dose group	9.84±0.61*	10.15±0.64#	0.31±0.26	2.94±0.14
3× dose group	9.86±0.83	10.31±0.91#	0.45±0.38	2.94±0.19
1× dose group	10.63±0.65	11.06±0.82#	0.44±0.27	3.11±0.14

Normal saline control group	10.61±0.38	10.99±0.48#	0.38±0.27	3.11±0.08

1. Body weight between groups was Analyzed by one-way ANOVA. post-hoc comparisons were performed using Dunnett's test.The mark "*" indicates that the treatment group is statistically significant from the Normal saline control group ($p < 0.05$).

2. Body weight gain between groups was Analyzed by one-way ANOVA. post-hoc comparisons were performed using Dunnett's test.

3. Paired t-tests were used to compare body weights before and after treatment. '#' indicates a statistically significant difference between Day 14 and Day 0 ($p < 0.05$).

11 Supplementary Table 11-Results of RBC examination (Unit: $\times 10^{12}\cdot L^{-1}$，M(P25,P75)，n=8)

Group	Assessment day			
	D0	D4	D7	D14
5× dose group	6.33(5.96,6.84)	6.03(5.52,6.73)	6.13(5.37,6.21)	6.25(5.81,6.95)
3× dose group	6.05(6.53,5.51)	6.29(5.58,6.90)	5.84(5.73,6.36)	6.22(5.19,6.70)
1× dose group	6.66(5.82,7.07)	7.11(6.80,7.32)	6.04(5.56,6.82)	6.87(5.85,7.19)
Normal saline control group	6.72(5.86,6.72)	7.09(5.47,8.03)	6.60(5.70,7.28)	6.55(5.52,8.52)

1.Analyzed using the Wilcoxon rank-sum test.

12 Supplementary Table 12- Results of HGB examination (Unit: $g\cdot L^{-1}$，M(P25,P75)，n=8)

Group	Assessment day			
	D0	D4	D7	D14
5× dose group	143.50(136.75,150.25)	144.00(139.25,152.75)	141.00(112.75,142.75)	140.50(135.00,153.00)
3× dose group	130.50(117.25,145.00)	145.00(130.00,151.00)	126.50(122.50,146.25)	135.00(114.25,145.00)
1× dose group	143.00(133.25,147.75)	154.00(137.00,171.00)	129.50(121.25,151.25)	141.50(132.50,158.25)

Normal saline control group	144.50(134.00,160.25)	167.00(135.50,174.75)	150.00(127.00,157.25)	140.00(129.00,187.00)

1. Analyzed using the Wilcoxon rank-sum test.

13 Supplementary Table 13-Results of HCT examination(Unit: %, M(P25,P75)，n=8)

Group	Assessment day			
	D0	D4	D7	D14
5× dose group	41.20(39.43,43.68)	39.45(37.43,45.25)	39.95(32.78,40.58)	39.75(38.73,43.73)
3× dose group	38.15(34.80,41.75)	41.95(37.60,45.13)	36.70(35.65,41.23)	39.05(33.43,41.80)
1× dose group	41.90(38.50,42.83)	45.50(41.40,48.70)	37.70(34.53,43.00)	41.10(37.85,44.63)
Normal saline control group	41.30(38.65,46.38)	47.65(36.43,51.73)	42.55(36.20,44.58)	39.90(36.50,53.00)

1.Analyzed using the Wilcoxon rank-sum test.

14 Supplementary Table 14-Results of PLT examination(Unit:$\times 10^9 \cdot L^{-1}$, M(P25,P75)，n=8)

Group	Assessment day			
	D0	D4	D7	D14
5× dose group	344.00(88.00,502.00)	235.00(210.50,267.00)	296.50(114.75,384.25)	301.00(222.50,438.00)
3× dose group	216.50(179.25,372.25)	211.00(173.50,337.50)	189.00(151.75,332.25)	198.50(153.75,243.75)

1× dose group	272.00(197.75,348.75)	257.00(170.00,295.00)	224.50(157.50,328.50)	250.50(59.50,254.25)
Normal saline control group	212.50(173.25,391.00)	215.50(199.75,387.75)	200.50(135.25,379.75)	208.00(122.00,318.00)

1. Analyzed using the Wilcoxon rank-sum test.

15 Supplementary Table 15- Results of WBC examination(Unit:×$10^9 \cdot L^{-1}$, M(P25,P75)，n=8)

Group	Assessment day			
	D0	D4	D7	D14
5× dose group	11.39(9.60,16.50)	11.34(9.01,13.41)	14.03(13.54,15.14)	13.93(11.79,14.44)
3× dose group	11.93(9.55,14.39)	9.97(8.81,11.75)	10.99(7.34,13.91)	9.76(7.93,13.94)*
1× dose group	10.95(10.17,15.13)	13.09(9.28,13.87)	11.03(7.47,14.48)	10.93(8.54,13.23)
Normal saline control group	13.65(12.19,15.23)	13.97(8.60,15.97)	10.49(8.27,14.12)	14.39(11.60,16.20)

1. Analyzed using the Wilcoxon rank-sum test. post-hoc comparisons were performed using the Bonferroni method.

2. The mark “*” indicates that the treatment group is statistically significant from the Normal saline control group ($p < 0.05$).

16 Supplementary Table 16- Results of NEUT examination(Unit:×$10^9 \cdot L^{-1}$, M(P25,P75)，n=8)

Group	Assessment day			
	D0	D4	D7	D14
5× dose group	7.61(5.21,11.19)	7.58(5.71,9.68)	8.80(8.31,10.26)	8.96(7.35,10.36)
3× dose group	7.78(6.50,9.09)	6.76(6.67,7.93)	7.61(4.89,7.77)	6.32(4.65,8.66)*
1× dose group	7.44(6.62,9.64)	8.76(6.91,9.15)	6.28(4.63,8.48)	6.17(5.38,7.03)*
Normal saline control group	9.22(8.59,10.34)	8.84(5.64,10.93)	6.62(5.63,8.67)	9.66(9.22,10.74)

1. Analyzed using the Wilcoxon rank-sum test. post-hoc comparisons were performed using the Bonferroni method.

2. The mark “*” indicates that the treatment group is statistically significant from the Normal saline control group ($p < 0.05$).

17 Supplementary Table 17- Results of LYM examination(Unit:$\times 10^{9} \cdot L^{-1}$, M(P25,P75)， n=8)

Group	Assessment day			
	D0	D4	D7	D14
5× dose group	2.95(2.45,4.11)	2.23(1.80,2.71)	3.14(2.16,4.00)	2.82(2.09,3.86)
3× dose group	2.82(2.11,3.45)	1.74(1.50,2.55)	2.23(1.78,3.82)	2.32(2.25,3.40)
1× dose group	2.91(2.02,3.19)	2.74(1.94,3.09)	3.09(1.64,3.60)	3.09(2.24,4.23)
Normal saline control group	3.12(2.02,3.69)	2.52(1.31,3.89)	2.95(1.81,3.60)	2.87(1.91,4.27)

1. Analyzed using the Wilcoxon rank-sum test.

18 Supplementary Table 18- Results of MONO examination(Unit:$\times 10^{9} \cdot L^{-1}$, M(P25,P75)， n=8)

Group	Assessment day			
	D0	D4	D7	D14
5× dose group	0.54(0.39,0.73)	0.64(0.53,0.69)	0.61(0.52,0.76)	0.49(0.36,0.63)
3× dose group	0.43(0.36,0.50)	0.55(0.41,0.58)	0.39(0.29,0.61)	0.38(0.31,0.48)
1× dose group	0.41(0.36,0.59)	0.57(0.44,0.58)	0.37(0.26,0.70)	0.43(0.31,0.48)
Normal saline control group	0.62(0.47,0.63)	0.61(0.55,0.66)	0.55(0.35,0.67)	0.56(0.45,0.65)

1. Analyzed using the Wilcoxon rank-sum test.

19 Supplementary Table 19- Results of UREA examination(Unit:mmol·L^{-1}, M(P25,P75)，n=8)

Group	Assessment day			
	D0	D4	D7	D14
5× dose group	3.26(2.51,4.56)	5.18(3.97,5.96)*	3.19(2.26,5.45)	3.57(3.09,4.14)
3× dose group	4.02(2.68,4.40)	8.29(6.26,9.22)	4.05(3.47,4.97)	4.26(3.01,5.13)
1× dose group	4.02(3.13,4.63)	7.18(6.83,7.26)	3.86(3.59,5.21)	4.36(3.53,5.01)
Normal saline control group	4.54(2.83,5.00)	7.28(5.63,8.89)	4.45(3.47,4.69)	4.42(3.48,5.43)

1. Analyzed using the Wilcoxon rank-sum test. post-hoc comparisons were performed using the Bonferroni method.

2. The mark "*" indicates that the treatment group is statistically significant from the Normal saline control group ($p < 0.05$).

20 Supplementary Table 20- Results of CREA examination(Unit:μmol·L^{-1}, M(P25,P75)，n=8)

Group	Assessment day			
	D0	D4	D7	D14
5× dose group	54.65(46.13,64.85)	50.00(41.93,63.83)	46.85(36.43,64.85)	61.95(37.38,71.78)
3× dose group	80.45(64.00,90.73)	69.25(51.63,71.20)	75.20(52.85,82.83)	87.15(60.35,90.60)
1× dose group	76.40(52.48,80.65)	68.30(43.90,78.30)	61.70(40.25,72.58)	76.85(52.25,80.38)
Normal saline control group	81.45(52.78,87.23)	67.30(49.05,76.45)	64.55(44.05,68.65)	84.70(70.10,93.70)

1. Analyzed using the Wilcoxon rank-sum test.

21 Supplementary Table 21- Results of CK examination(Unit:U·L^{-1}, M(P25,P75，n=8)

Group	Assessment day			
	D0	D4	D7	D14
5× dose group	99.95(85.98,108.10)	134.45(87.58,167.63)	122.30(93.15,139.90)	71.95(62.30,83.03)
3× dose group	121.30(109.70,167.93)	120.15(111.83,150.13)	133.50(131.15,137.45)	83.25(72.50,97.05)
1× dose group	112.55(70.38,146.18)	121.10(63.80,143.70)	125.20(87.40,144.40)	89.85(56.35,92.53)
Normal saline control group	126.25(89.90,143.10)	145.30(104.35,150.98)	126.50(116.90,148.85)	84.40(80.60,102.20)

1. Analyzed using the Wilcoxon rank-sum test.

22 Supplementary Table 22- Results of AST examination(Unit:U·L^{-1}, M(P25,P75)，n=8)

Group	Assessment day			
	D0	D4	D7	D14
5× dose group	22.40(20.73,29.13)	20.25(17.85,27.68)	25.00(23.83,33.13)	22.30(21.10,29.93)
3× dose group	26.10(25.65,39.25)	26.55(22.08,41.23)	28.95(27.23,44.65)	24.65(21.13,41.68)
1× dose group	25.40(17.05,49.73)	22.40(15.60,25.30)	24.65(19.65,27.25)	22.65(16.80,25.80)
Normal saline control group	24.35(20.60,31.93)	27.70(19.78,33.23)	24.50(22.05,34.88)	25.70(18.70,26.40)

1. Analyzed using the Wilcoxon rank-sum test.

23 Supplementary Table 23- Results of ALT examination(Unit:U·L^{-1}, M(P25,P75)，n=8)

Group	Assessment day			
	D0	D4	D7	D14
5× dose group	26.10(21.00,35.28)	26.80(20.00,32.50)	26.20(21.98,36.75)	30.30(22.85,35.83)
3× dose group	23.80(19.53,25.78)	25.15(23.20,26.98)	24.90(23.48,25.98)	23.65(21.70,28.93)
1× dose group	31.65(22.65,63.53)	36.00(29.00,42.50)	30.65(22.00,34.43)	31.60(20.43,45.13)
Normal saline control group	25.50(24.10,33.88)	33.70(22.70,38.28)	27.90(22.75,36.48)	24.80(22.90,27.80)

1. Analyzed using the Wilcoxon rank-sum test.

24 Supplementary Table 24- Results of ALP examination(Unit:U·L^{-1}, M(P25,P75)，n=8)

Group	Assessment day			
	D0	D4	D7	D14
5× dose group	44.35(24.45,93.08)	58.55(32.75,99.13)	38.30(17.93,85.20)	37.15(21.28,86.88)
3× dose group	58.25(47.48,77.20)	57.55(20.75,63.28)	59.35(16.20,68.23)	49.40(17.38,66.60)
1× dose group	54.75(44.93,60.73)	51.50(47.00,65.90)	52.15(45.20,55.58)	54.95(51.40,60.75)
Normal saline control group	35.60(30.78,54.18)	35.10(33.58,55.23)	39.30(28.98,63.85)	44.30(33.90,50.70)

1. Analyzed using the Wilcoxon rank-sum test.

25 Supplementary Table 25- Results of GGT examination(Unit:U·L^{-1}, $\bar{X}$±SD，n=8)

Group	Assessment day			
	D0	D4	D7	D14
5× dose group	3.41±1.02	1.53±0.63	1.40±1.03	3.60±0.89
3× dose group	2.84±0.54	2.09±1.08	1.34±0.78	3.36±0.59
1× dose group	3.90±1.13	2.43±1.43	1.94±1.35	4.20±1.30
Normal saline control group	3.40±1.62	2.53±1.38	1.75±1.29	4.29±1.54

1.Analyzed using Dunnett's test in one-way ANOVA.

26 Supplementary Table 26- Results of ALP examination(Unit:μmol·L^{-1}, M(P25,P75)，n=8)

Group	Assessment day			
	D0	D4	D7	D14
5× dose group	1.08(0.77,1.30)	1.20(0.90,2.14)	1.03(0.81,1.80)	1.75(1.05,1.98)
3× dose group	1.24(0.84,1.50)	1.28(1.04,1.69)	1.35(0.84,1.64)	1.53(0.51,1.86)
1× dose group	0.97(0.79,1.26)	1.21(1.04,1.63)	1.07(0.97,1.56)	1.37(0.88,1.69)
Normal saline control group	1.33(1.07,1.49)	1.33(0.81,1.54)	1.32(0.86,1.84)	1.76(1.31,2.16)

1. Analyzed using the Wilcoxon rank-sum test.

27 Supplementary Table 27- Results of TP examination(Unit:g·L^{-1}, M(P25,P75)，n=8)

Group	Assessment day			
	D0	D4	D7	D14
5× dose group	57.00(54.21,60.63)*	58.19(54.60,63.12)	60.13(52.67,63.58)	56.07(55.23,62.04)
3× dose group	50.64(48.78,53.83)	57.30(54.16,61.04)	52.81(51.13,54.47)	51.65(50.45,54.50)
1× dose group	53.24(51.05,54.94)	62.84(55.04,63.26)	54.26(54.02,55.51)	53.94(53.39,54.70)
Normal saline control group	49.99(46.14,56.58)	56.87(53.77,58.61)	53.17(49.23,58.87)	55.00(50.24,56.72)

1. Analyzed using the Wilcoxon rank-sum test. post-hoc comparisons were performed using the Bonferroni method.

2. The mark “*” indicates that the treatment group is statistically significant from the Normal saline control group ($p < 0.05$).

28 Supplementary Table 28- Results of ALB examination(Unit:g·L^{-1}, M(P25,P75)，n=8)

Group	Assessment day			
	D0	D4	D7	D14
5× dose group	21.60(18.53,22.60)	22.10(18.95,25.65)	23.30(19.10,24.03)	21.35(19.23,22.88)
3× dose group	18.45(17.93,19.25)*	22.25(19.20,24.40)	20.80(20.50,22.98)	20.45(18.15,21.30)
1× dose group	21.85(20.00,22.68)	24.10(23.60,25.90)	22.50(21.10,23.30)	21.65(20.88,22.98)
Normal saline control group	20.45(19.43,21.33)	22.30(21.00,24.53)	22.15(21.55,23.08)	21.10(20.30,21.60)

1. Analyzed using the Wilcoxon rank-sum test. post-hoc comparisons were performed using the Bonferroni method.

2. The mark "*" indicates that the treatment group is statistically significant from the Normal saline control group ($p < 0.05$).

29 Supplementary Table 29- Results of GLB examination(Unit:g·L^{-1}, $\bar{X}$±SD，n=8)

Group	Assessment day			
	D0	D4	D7	D14
5× dose group	36.06±3.09*	36.45±2.34	36.58±3.96	36.69±3.03
3× dose group	32.29±2.70	35.73±3.02	31.53±3.07	32.32±2.39
1× dose group	31.62±2.16	35.14±3.34	32.50±2.18	32.19±1.08
Normal saline control group	30.28±5.11	33.85±3.47	31.50±4.70	32.05±4.01

1.Analyzed by one-way ANOVA. post-hoc comparisons were performed using Dunnett's test.

2. Paired t-tests were used to compare body weights before and after treatment. '#' indicates a statistically significant difference between Day 14 and Day 0 ($p < 0.05$).

30 Supplementary Table 30- Results of GLU examination(Unit:mmol·L^{-1}, M(P25,P75)，n=8)

Group	Assessment day			
	D0	D4	D7	D14
5× dose group	4.75(4.28,5.20)	4.24(4.05,5.07)	4.95(4.61,5.00)	5.00(4.59,7.27)
3× dose group	4.73(4.59,5.19)	4.31(4.04,5.10)	5.00(4.75,5.04)	4.93(4.84,5.07)
1× dose group	4.45(4.27,4.78)	4.23(3.92,4.37)*	5.09(4.59,5.28)	4.79(4.56,5.09)
Normal saline control group	4.65(4.14,4.73)	3.92(3.65,4.05)	4.68(4.62,4.77)	4.02(3.92,4.98)

1. Analyzed using the Wilcoxon rank-sum test. post-hoc comparisons were performed using the Bonferroni method.

2. The mark "*" indicates that the treatment group is statistically significant from the Normal saline control group ($p < 0.05$).

31 Supplementary Table 31- Results of TG examination(Unit:mmol·L^{-1}, M(P25,P75)，n=8)

Group	Assessment day			
	D0	D4	D7	D14
5× dose group	0.46(0.34,0.69)	0.50(0.31,0.77)	0.49(0.37,0.59)	0.62(0.53,0.69)
3× dose group	0.51(0.37,0.61)	0.63(0.44,0.87)	0.46(0.31,0.53)	0.47(0.29,0.56)
1× dose group	0.46(0.44,0.52)	1.28(0.70,1.76)	0.45(0.36,0.47)	0.54(0.44,0.63)
Normal saline	0.45(0.31,0.59)	0.82(0.65,1.27)	0.50(0.29,0.73)	0.52(0.47,0.82)

control group

1. Analyzed using the Wilcoxon rank-sum test.

32 Supplementary Table 32- Results of TC examination(Unit:mmol·L^{-1}, M(P25,P75)，n=8)

Group	Assessment day			
	D0	D4	D7	D14
5× dose group	3.35±0.45	3.72±0.32	3.45±0.65	3.35±0.50
3× dose group	3.03±0.25*	3.76±0.33	3.24±0.17	3.10±0.22*
1× dose group	3.72±0.59	4.35±0.99	3.81±0.55	3.78±0.47
Normal saline control group	3.45±0.25	3.96±0.42	3.58±0.24	3.72±0.38

1.Analyzed by one-way ANOVA. post-hoc comparisons were performed using Dunnett's test.

2. Paired t-tests were used to compare body weights before and after treatment. '#' indicates a statistically significant difference between Day 14 and Day 0 ($p < 0.05$).

33 Supplementary Table 33- Results of TCa examination(Unit:mmol·L^{-1}, M(P25,P75)，n=8)

Group	Assessment day			
	D0	D4	D7	D14
5× dose group	2.18(2.11,2.20)	2.16(2.08,2.26)	2.25(2.22,2.47)	2.29(2.16,2.37)
3× dose group	2.21(2.14,2.28)	2.27(2.23,2.38)	2.38(2.25,2.41)	2.28(2.22,2.34)
1× dose group	2.22(1.95,2.27)	2.35(2.14,2.51)	2.32(2.17,2.47)	2.26(2.13,2.48)

Normal saline control group	2.15(2.03,2.27)	2.22(2.15,2.32)	2.34(2.23,2.46)	2.27(2.17,2.34)

1. Analyzed using the Wilcoxon rank-sum test.

34 Supplementary Table 34- Results of P examination(Unit:mmol·L^{-1}, M(P25,P75)， n=8)

Group	Assessment day			
	D0	D4	D7	D14
5× dose group	1.20(0.95,1.82)	1.29(1.07,1.37)	1.13(0.98,1.26)	0.99(0.94,1.14)
3× dose group	1.79(1.37,2.26)	1.37(1.20,1.42)	1.41(0.97,1.52)	1.33(0.93,1.41)
1× dose group	1.78(1.13,2.15)	1.15(0.97,1.34)	1.19(0.90,1.46)	1.12(0.86,1.36)
Normal saline control group	1.53(0.97,1.90)	0.96(0.81,1.33)	1.06(0.87,1.40)	1.17(0.81,1.29)

1. Analyzed using the Wilcoxon rank-sum test.

35 Supplementary Table 35-Abbreviations and Reference Ranges for Hematological Parameters

Abbreviations	Full name	Unit	Reference Range
WBC	White Blood Cell Count	$\times10^9\cdot L^{-1}$	6.00～17.00
RBC	Red Blood Cell Count	$\times10^{12}\cdot L^{-1}$	5.10～8.50
HGB	Hemoglobin	$g\cdot L^{-1}$	110.00～190.00
HCT	Hematocrit	%	33.00～56.00
PLT	Platelet Count	$\times10^9\cdot L^{-1}$	117.00～490.00
NEUT	Neutrophil Count	$\times10^9\cdot L^{-1}$	3.62～12.30
LYM	Lymphocyte Count	$\times10^9\cdot L^{-1}$	0.83～4.91
MONO	Monocyte Count	$\times10^9\cdot L^{-1}$	0.14～1.97

1. Data were obtained from the Mindray BC-5000VET Hematology Analyzer.

35 Supplementary Table 35-Abbreviations and Reference Ranges for Biochemical Parameters

Abbreviations	Full name	Unit	Reference Range
TCa	Total Calcium	mmol·L^{-1}	1.95~3.15
P	Inorganic Phosphorus	mmol·L^{-1}	0.81~3.35
TC	Total Cholesterol	mmol·L^{-1}	1.07~10.34
GLU	Glucose	mmol·L^{-1}	3.14~8.33
CREA	Creatinine	μmol·L^{-1}	27.0~159.0
TBIL	Total Bilirubin	μmol·L^{-1}	0.00~15.00
ALT	Alanine Aminotransferase	U·L^{-1}	8.00~100.00
AST	Aspartate Aminotransferase	U·L^{-1}	0.00~50.00
ALP	Alkaline Phosphatase	U·L^{-1}	23.00~337.00
GGT	Gamma-Glutamyl Transferase	U·L^{-1}	0.00~7.00
TP	Total Protein	g·L^{-1}	48.00~82.00
ALB	Albumin	g·L^{-1}	21.00~40.00
UREA	Urea	mmol·L^{-1}	2.50~10.40
GLOB	Globulin	g·L^{-1}	8.00~61.00
CK	Creatine Kinase	U·L^{-1}	10.00~436.00
TG	Triglyceride	mmol·L^{-1}	0.22~1.20

1. Data were obtained from the Mindray BS-240 Fully Automatic Biochemical Analyzer.

36 Supplementary Picture 1 Chromatograms for specificity assessment

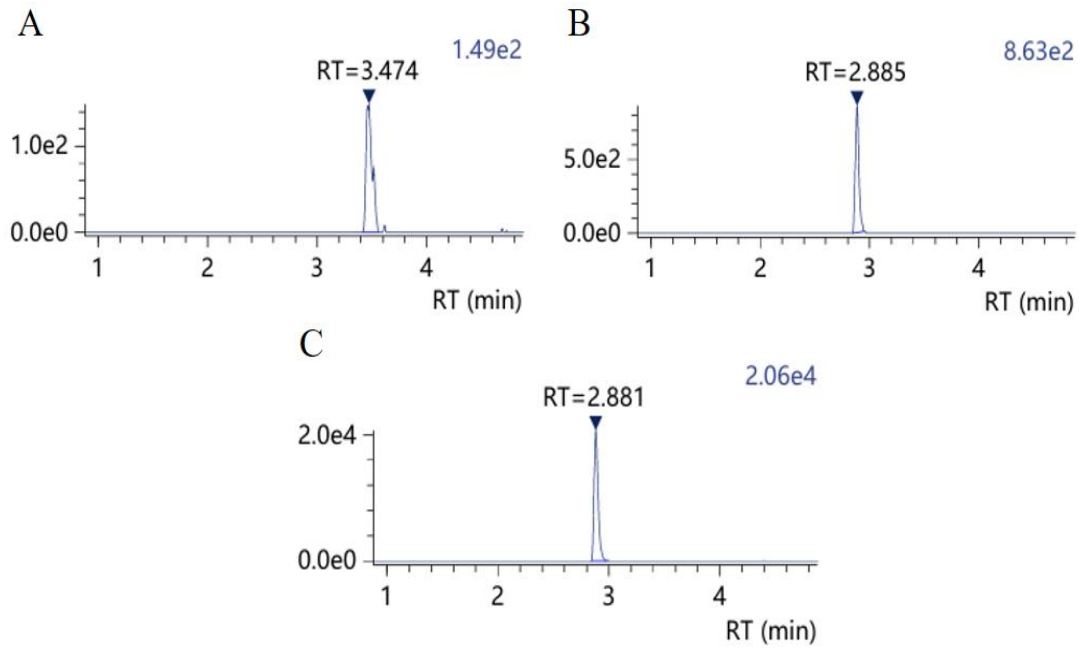

1.A: Chromatogram of water; B:Chromatogram of blank plasma sample ; C: Chromatogram of LLOQ sample(2 μg/mL).

37 Supplementary Picture 2 System Carryover Chromatogram

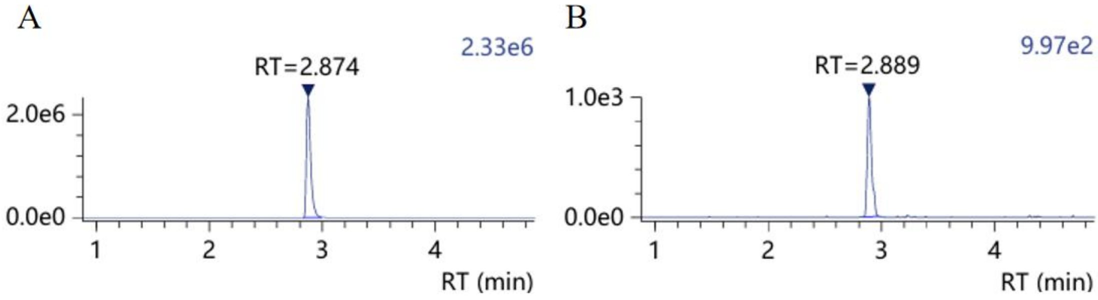

1.A: Chromatogram of ULOQ sample (20 μg/mL); B:Post-High-Concentration Blank Sample Chromatogram.

38 Supplementary Picture 3 Injection site manifestations

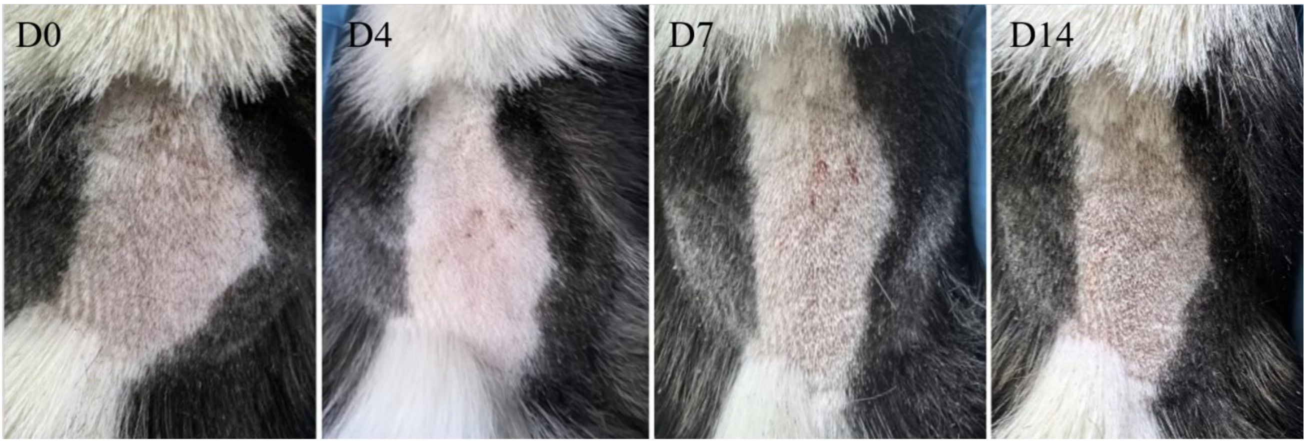

1. D0: Pre-dose; D4: Mid-dosing period; D7: end of dosing period; 7 days post-dosing.

39 Histopathological sections

5× dose group——Histopathological sections of Canine No. 08

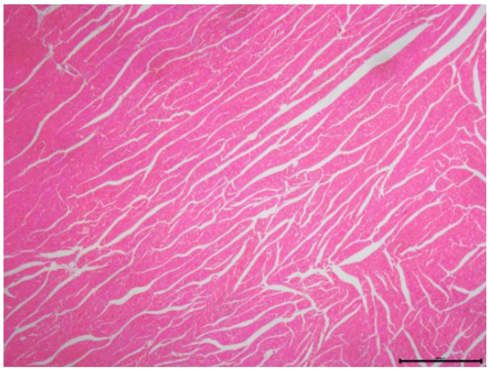

Canine myocardial tissue section

(4×，H.E.)

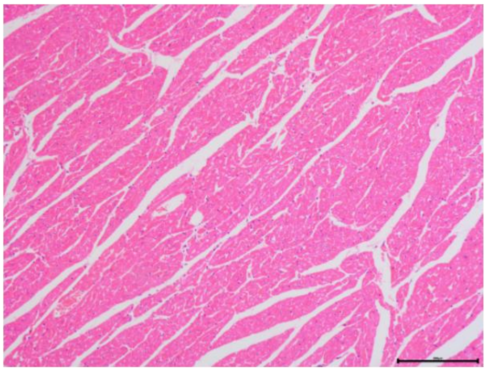

Canine myocardial tissue section

(10×，H.E.)

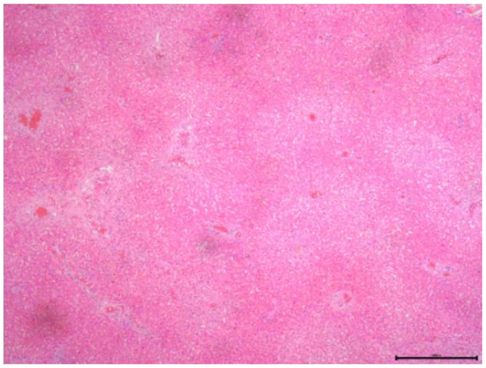

Canine liver tissue section

(4×，H.E.)

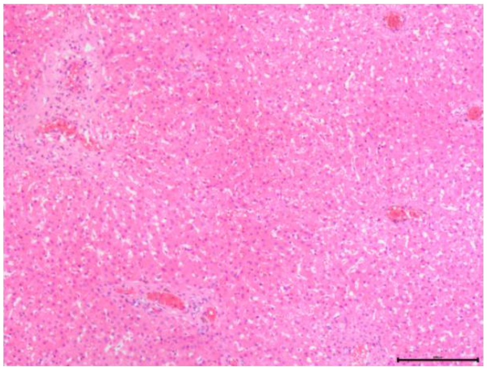

Canine liver tissue section

(10×，H.E.)

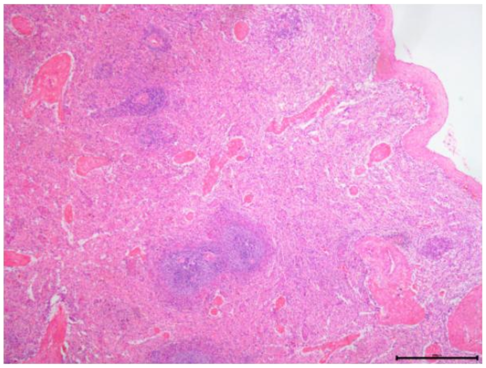

Canine spleen tissue section

(4×，H.E.)

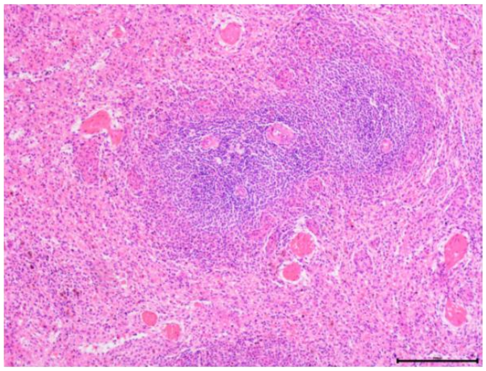

Canine spleen tissue section

(10×，H.E.)

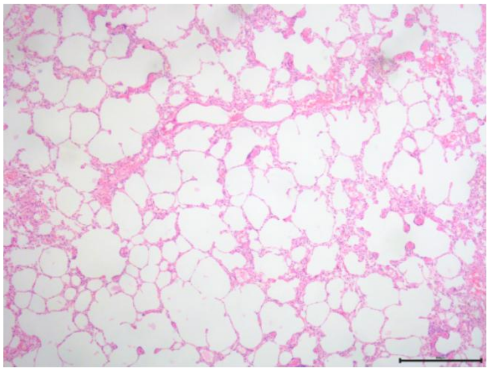

Canine lung tissue section

(4×，H.E.)

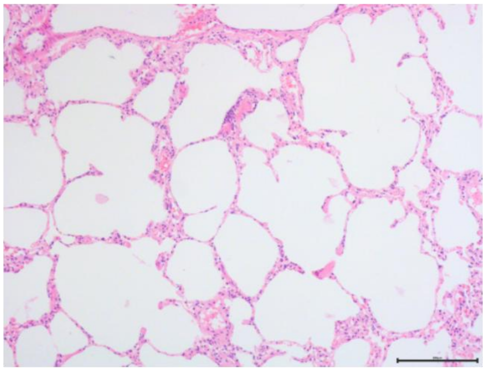

Canine lung tissue section

(10×，H.E.)

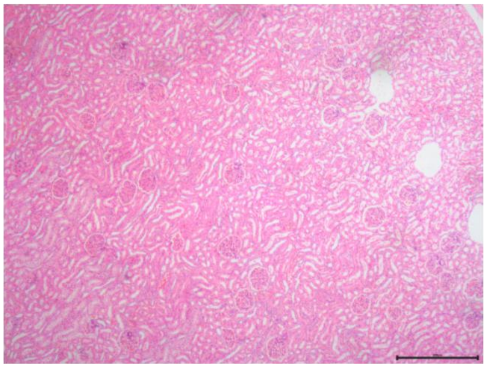

Canine kidney tissue section

(4×，H.E.)

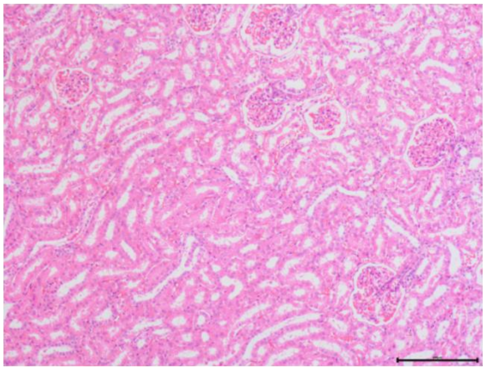

Canine kidney tissue section

(10×，H.E.)

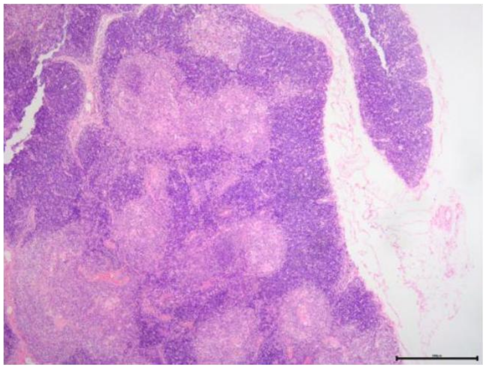

Canine thymus tissue section

(4×，H.E.)

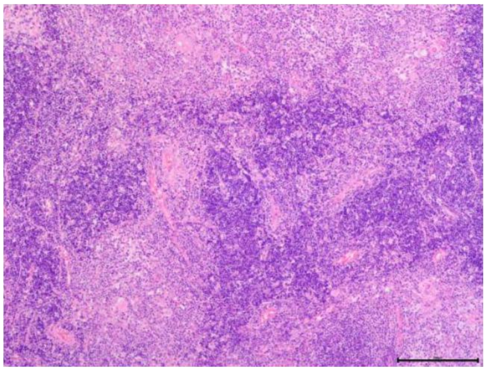

Canine thymus tissue section

(10×，H.E.)

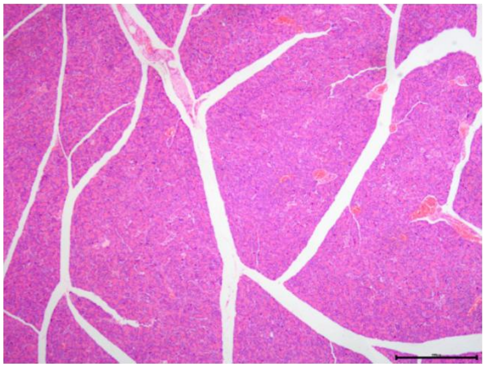

Canine pancreas tissue section

(4×, H.E.)

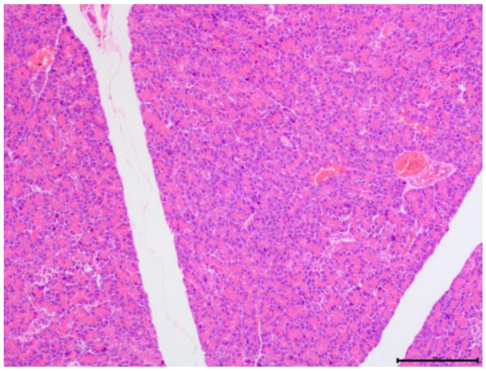

Canine pancreas tissue section

(10×, H.E.)

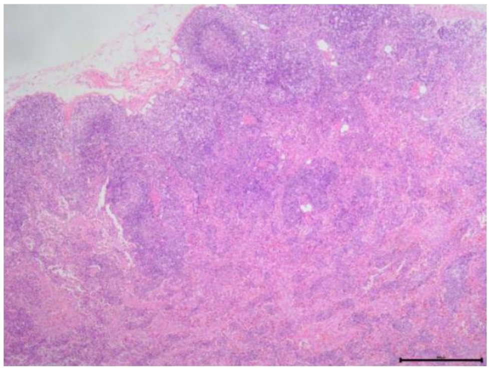

Canine lymph node tissue section

(4×, H.E.)

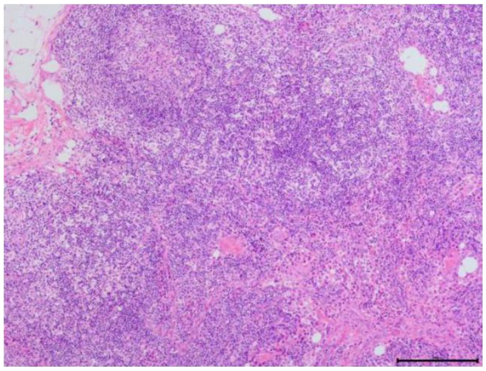

Canine lymph node tissue section

(10×, H.E.)

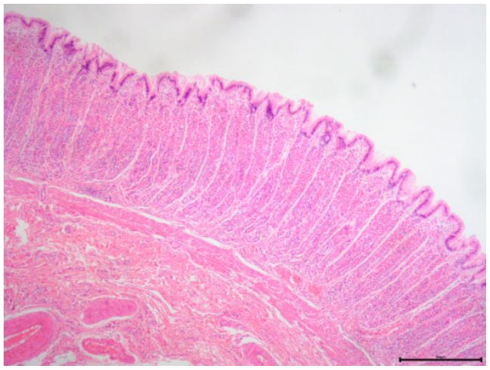

Canine stomach tissue section

(4×, H.E.)

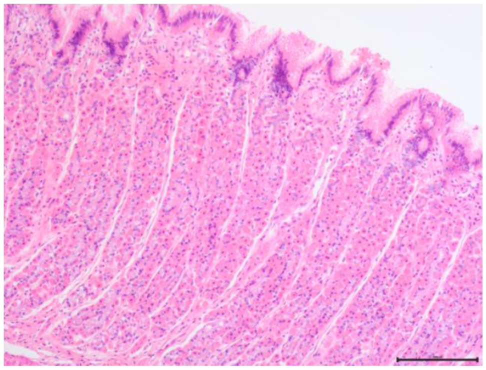

Canine stomach tissue section

(10×, H.E.)

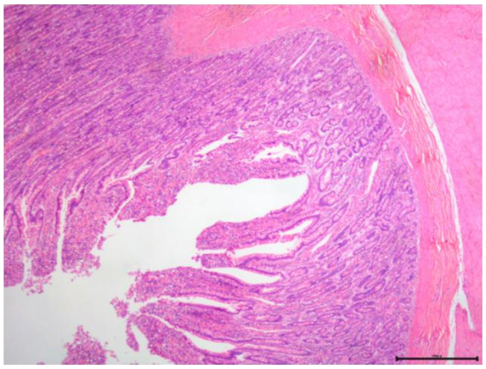

Canine duodenum tissue section

(4×， H.E.)

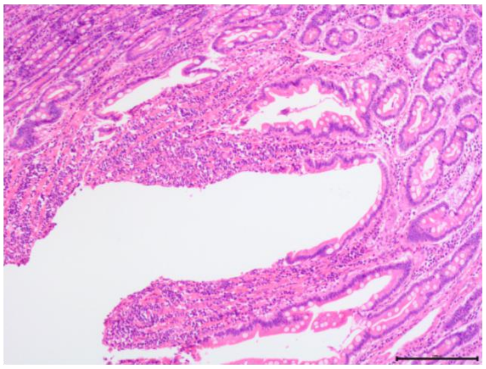

Canine duodenum tissue section

(10×， H.E.)

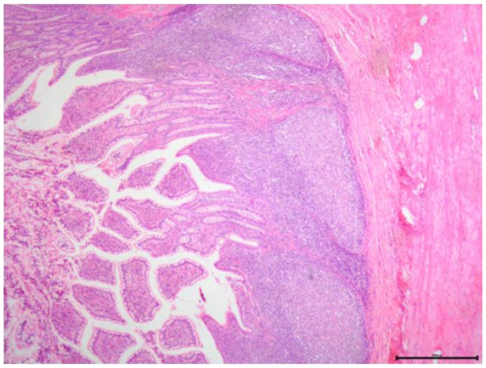

Canine ileum tissue section

(10×， H.E.)

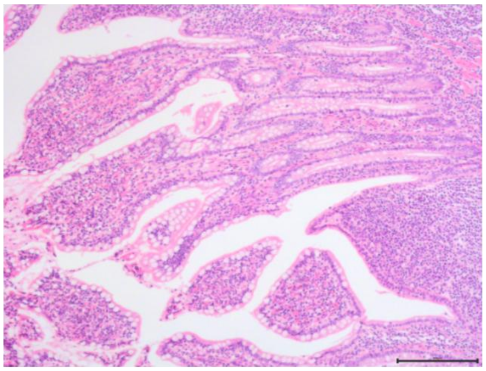

Canine ileum tissue section

(10×， H.E.)

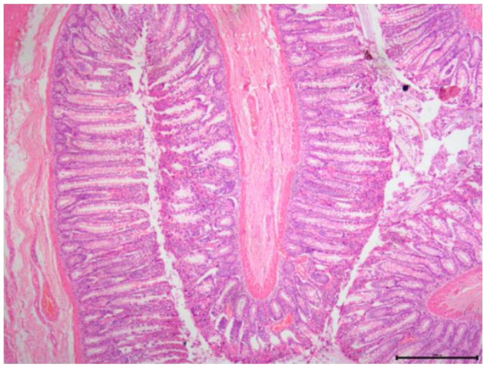

Canine rectum tissue section

(4×， H.E.)

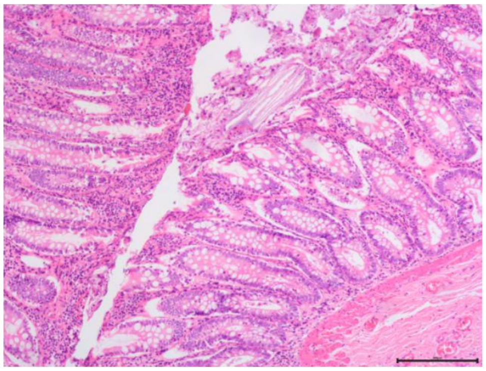

Canine rectum tissue section

(10×， H.E.)

5× dose group——Histopathological sections of Canine No. 03

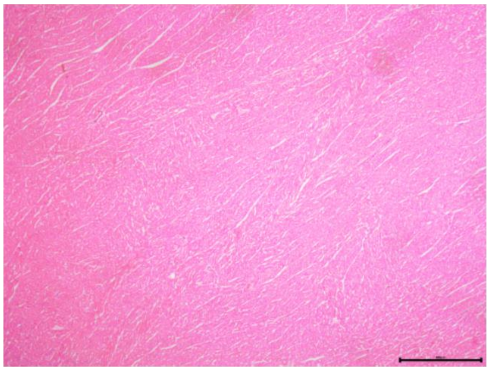

Canine myocardial tissue section(4×，H.E.)

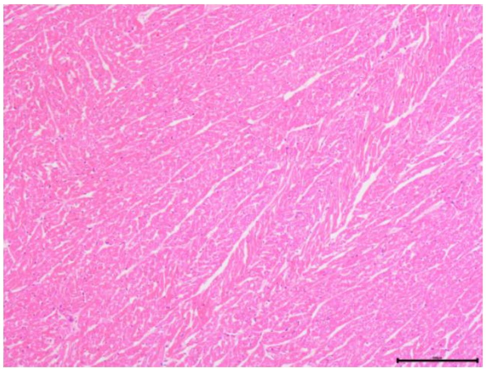

Canine myocardial tissue section(10×，H.E.)

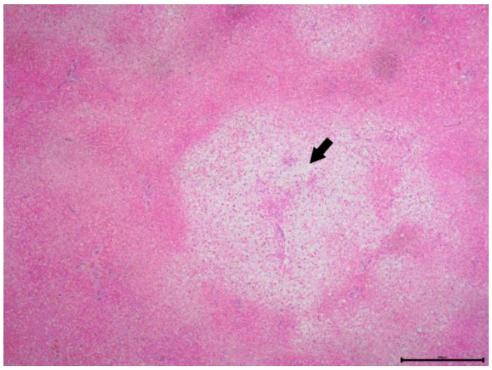

Canine liver tissue section [*1]

(4×，H.E.)

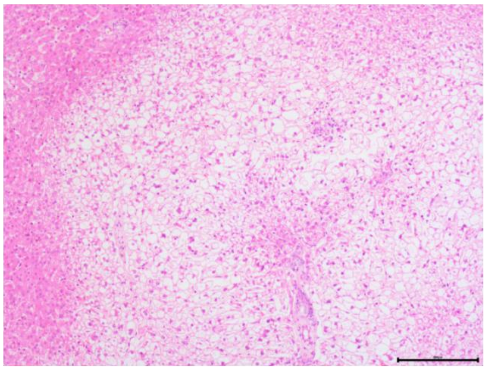

Canine liver tissue section

(10×，H.E.)

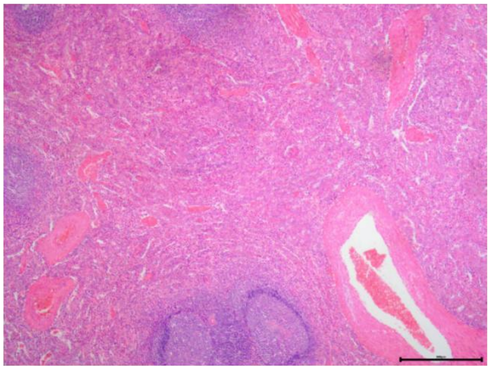

Canine spleen tissue section

(4×，H.E.)

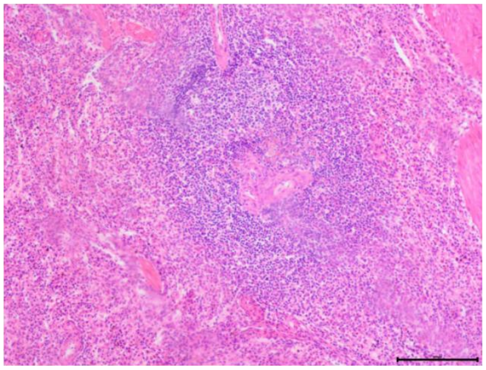

Canine spleen tissue section

(10×，H.E.)

"*1": Diffuse hepatic vacuolar degeneration

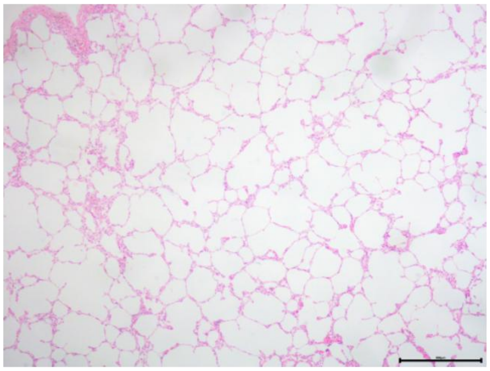

Canine lung tissue section

(4×, H.E.)

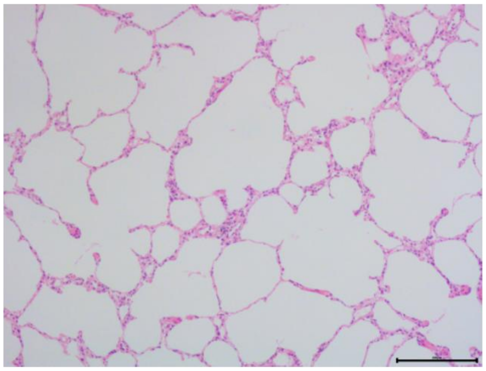

Canine lung tissue section

(10×, H.E.)

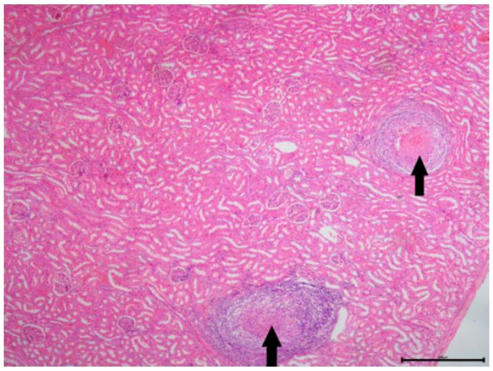

Canine kidney tissue section*2

(4×, H.E.)

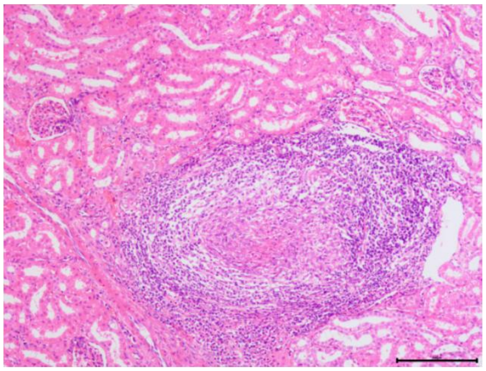

Canine kidney tissue section

(10×, H.E.)

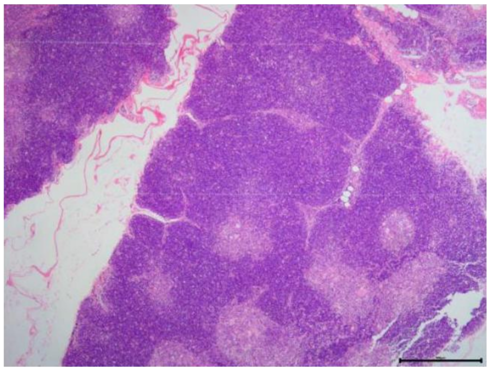

Canine thymus tissue section

(4×, H.E.)

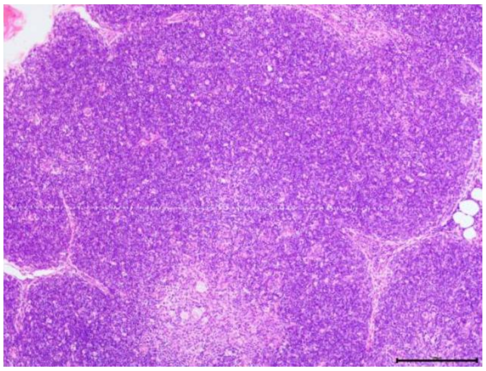

Canine thymus tissue section

(10×, H.E.)

"*2": Lymphoid follicle hyperplasia in the renal cortex.

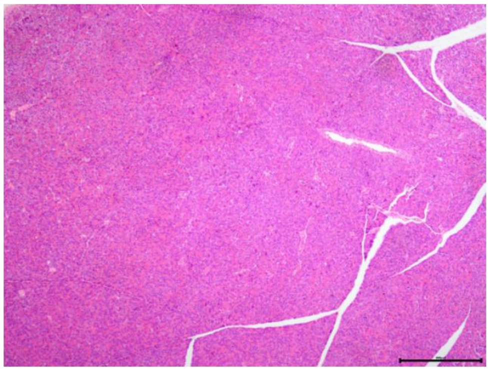

Canine pancreas tissue section

(4×，H.E.)

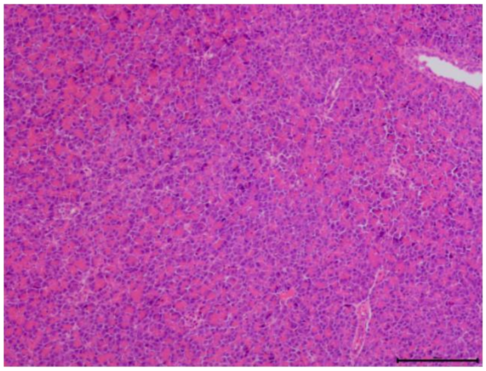

Canine pancreas tissue section

(10×，H.E.)

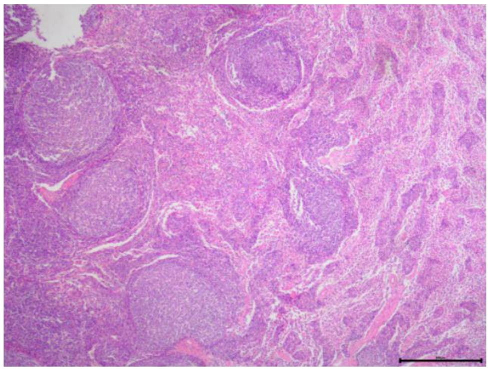

Canine lymph node tissue section

(4×，H.E.)

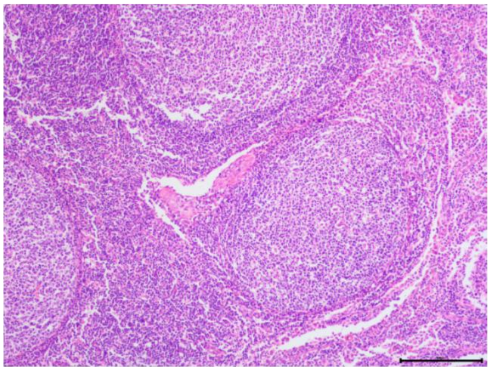

Canine lymph node tissue section

(10×，H.E.)

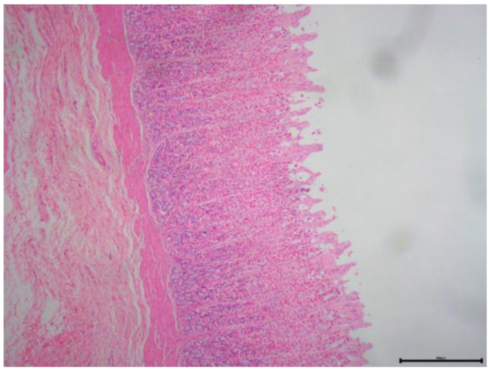

Canine stomach tissue section

(4×，H.E.)

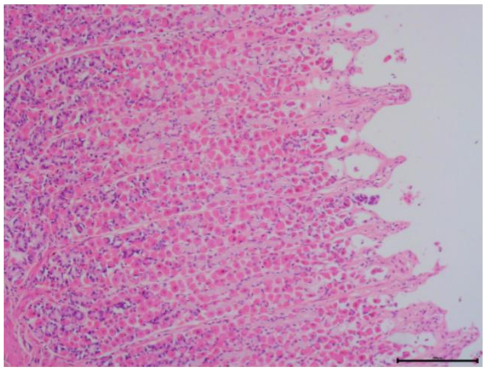

Canine stomach tissue section

(10×，H.E.)

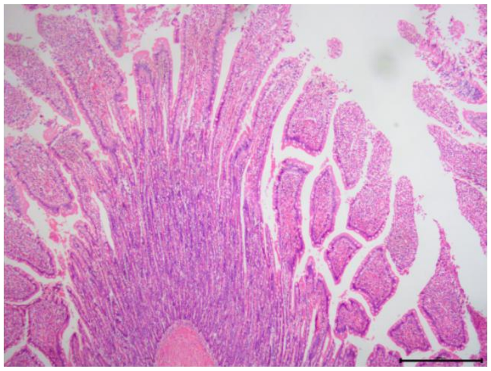

Canine duodenum tissue section

(4×， H.E.)

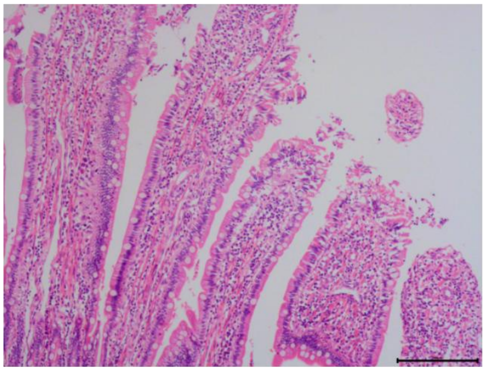

Canine duodenum tissue section

(10×， H.E.)

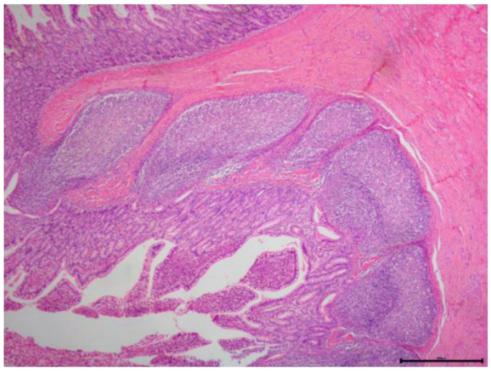

Canine ileum tissue section

(10×， H.E.)

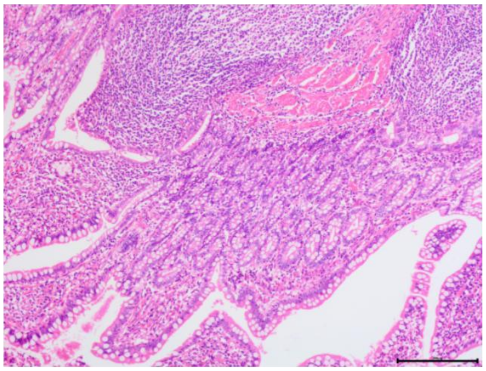

Canine ileum tissue section

(10×， H.E.)

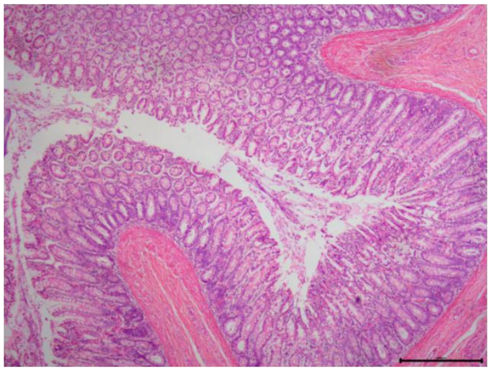

Canine rectum tissue section

(4×， H.E.)

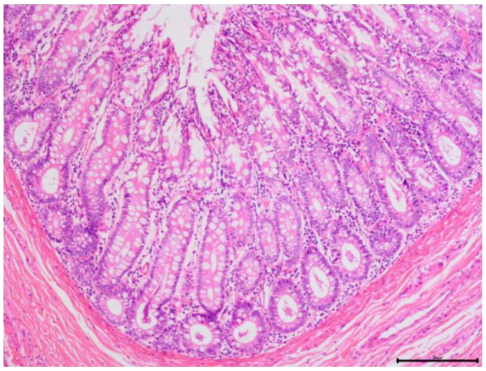

Canine rectum tissue section

(10×， H.E.)

5× dose group——Histopathological sections of Canine No. 16

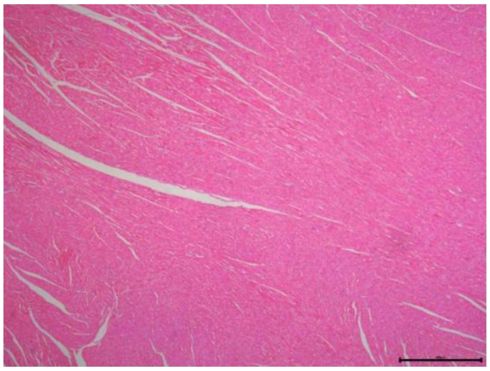

Canine myocardial tissue section(4×，H.E.)

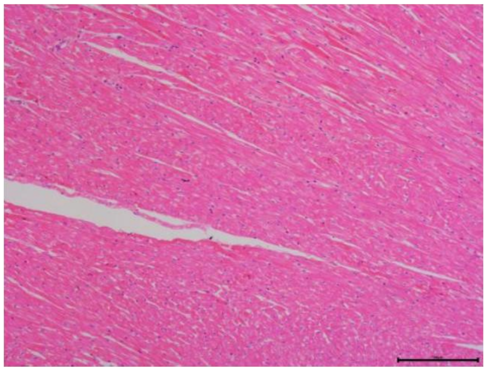

Canine myocardial tissue section(10×，H.E.)

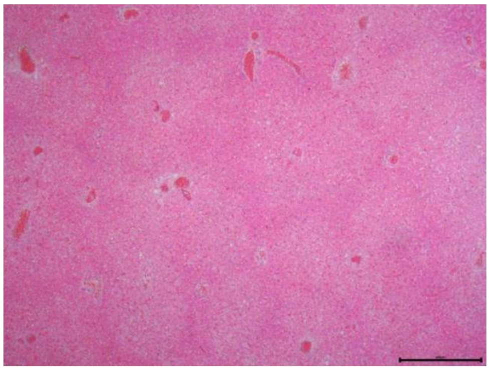

Canine liver tissue section

(4×，H.E.)

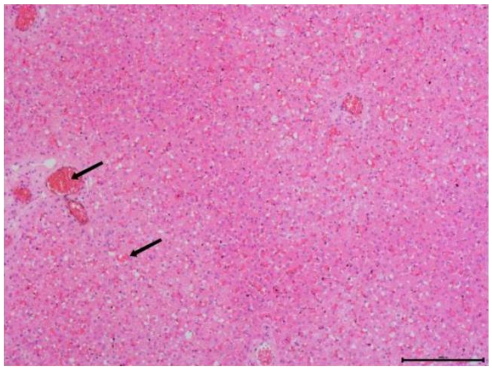

Canine liver tissue section*1

(10×，H.E.)

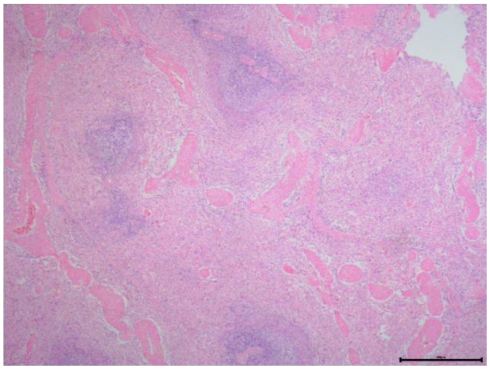

Canine spleen tissue section

(4×，H.E.)

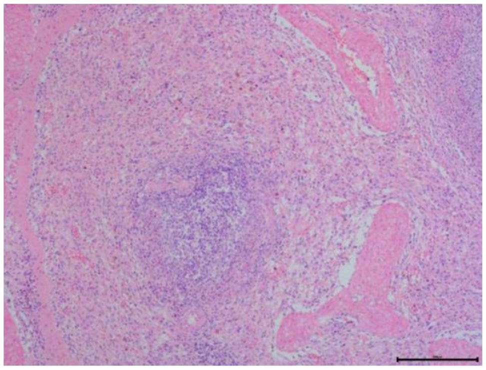

Canine spleen tissue section

(10×，H.E.)

"*1": Congestion of hepatic vessels and sinusoids

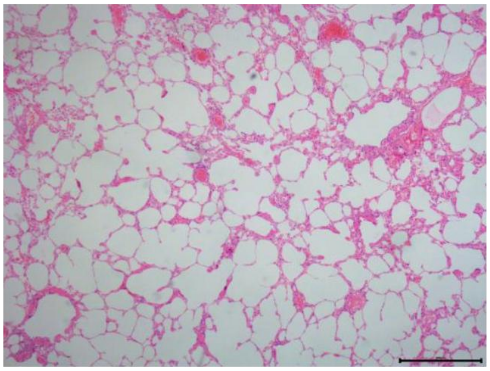

Canine lung tissue section

(4×，H.E.)

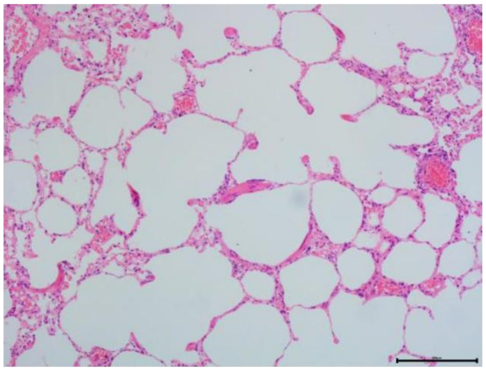

Canine lung tissue section

(10×，H.E.)

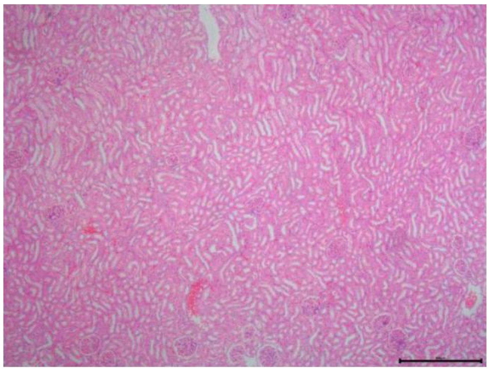

Canine kidney tissue section

(4×，H.E.)

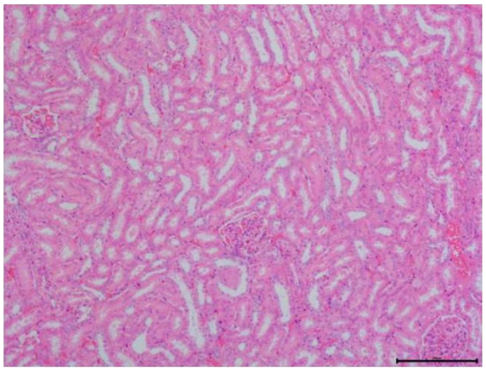

Canine kidney tissue section

(10×，H.E.)

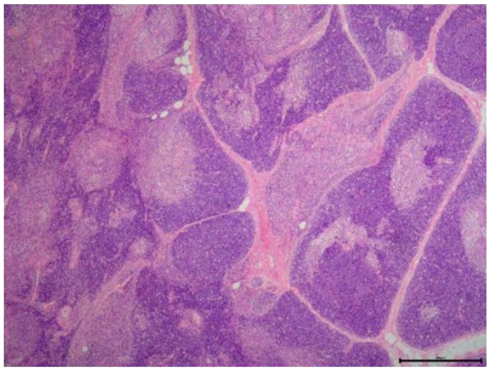

Canine thymus tissue section

(4×，H.E.)

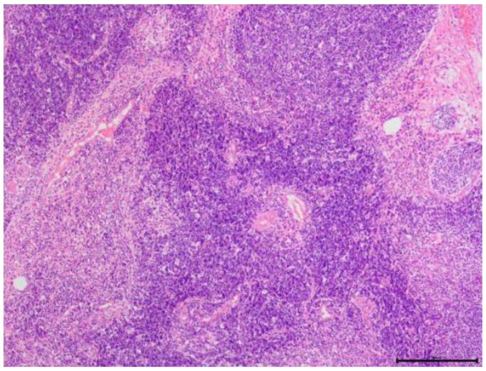

Canine thymus tissue section

(10×，H.E.)

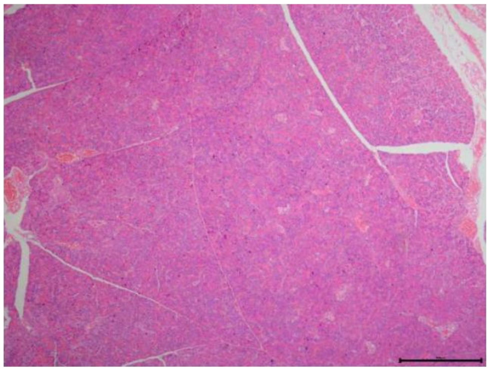

Canine pancreas tissue section

(4×， H.E.)

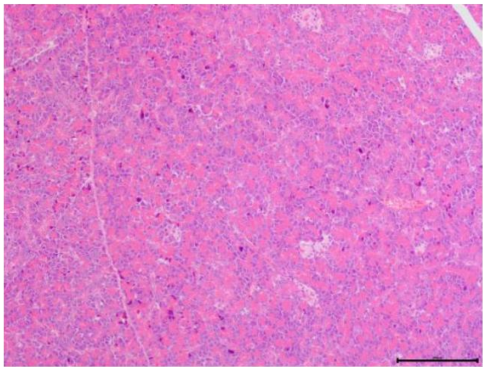

Canine pancreas tissue section

(10×， H.E.)

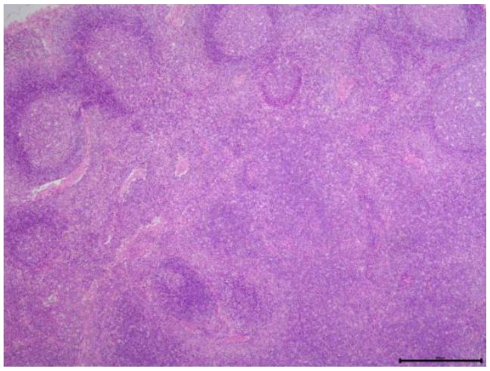

Canine lymph node tissue section

(4×， H.E.)

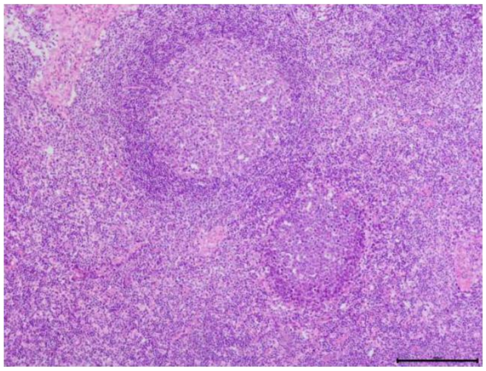

Canine lymph node tissue section

(10×， H.E.)

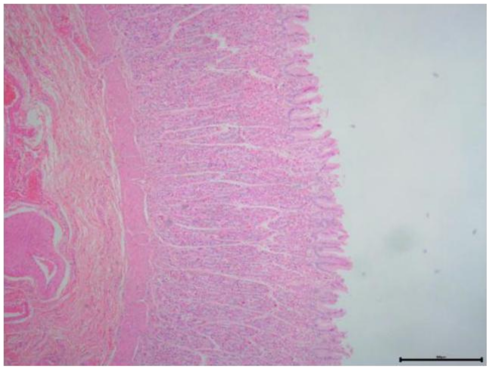

Canine stomach tissue section

(4×， H.E.)

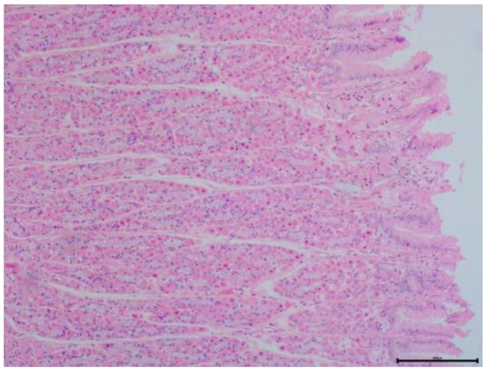

Canine stomach tissue section

(10×， H.E.)

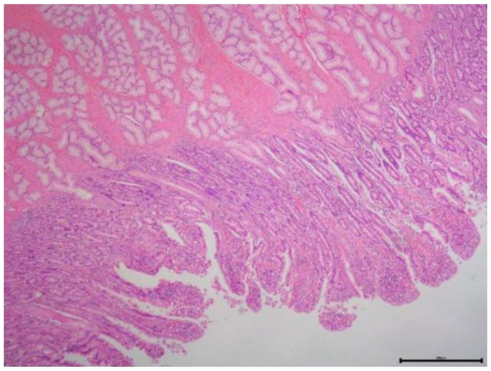

Canine duodenum tissue section

(4×，H.E.)

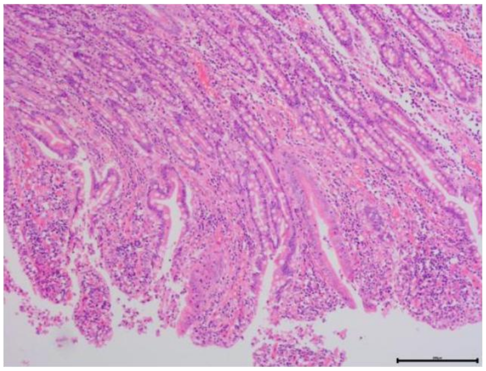

Canine duodenum tissue section

(10×，H.E.)

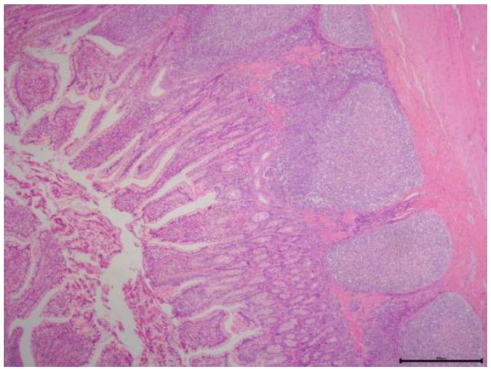

Canine ileum tissue section

(10×，H.E.)

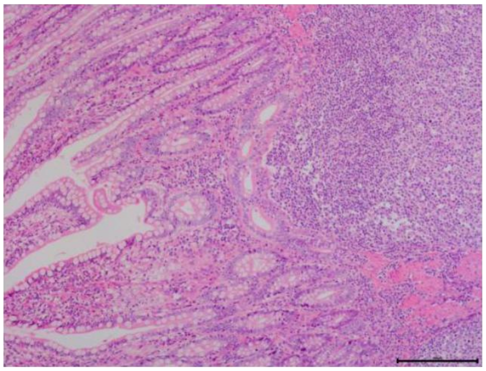

Canine ileum tissue section

(10×，H.E.)

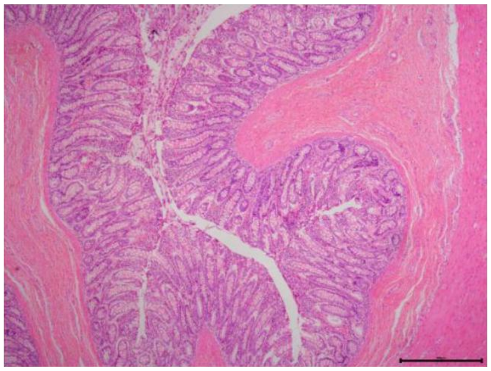

Canine rectum tissue section

(4×，H.E.)

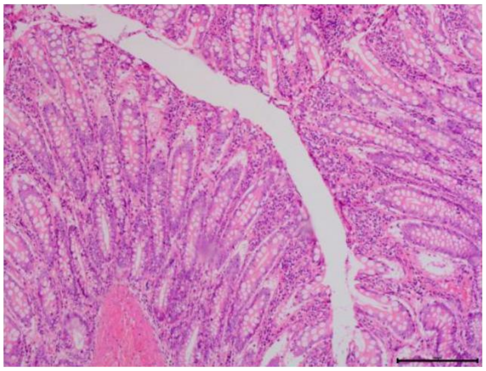

Canine rectum tissue section

(10×，H.E.)

5× dose group——Histopathological sections of Canine No. 21

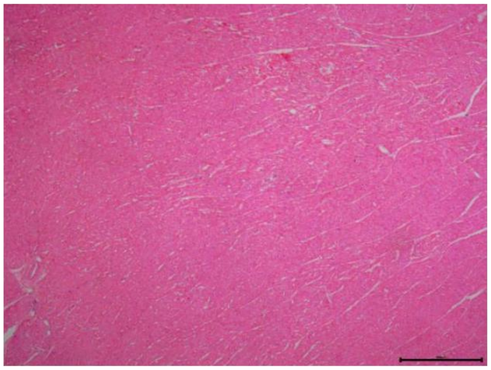

Canine myocardial tissue section(4×，H.E.)

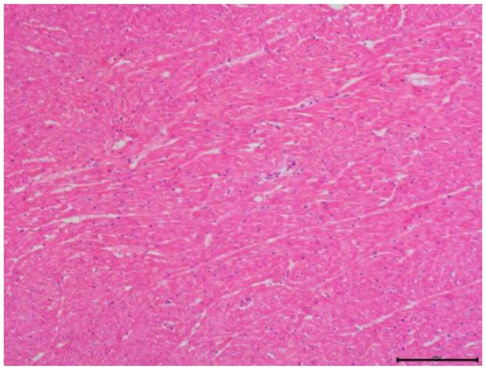

Canine myocardial tissue section(10×，H.E.)

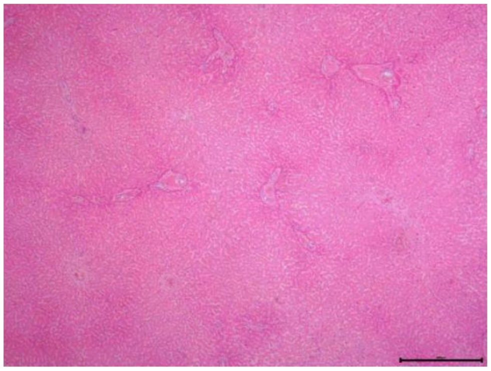

Canine liver tissue section

(4×，H.E.)

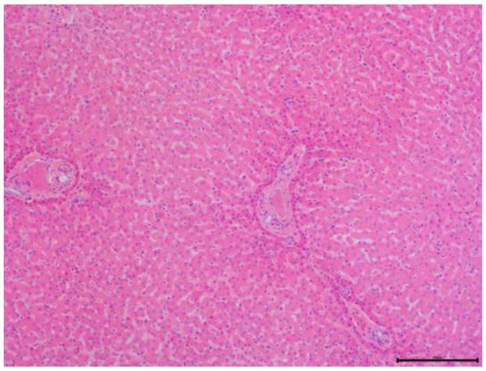

Canine liver tissue section

(10×，H.E.)

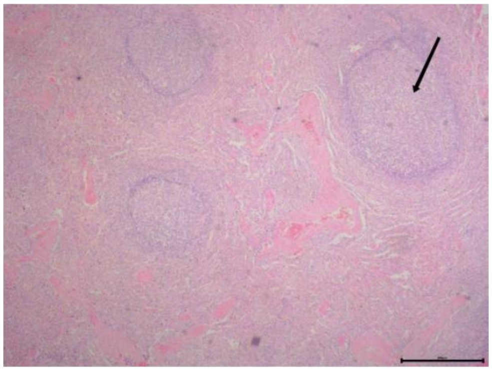

Canine spleen tissue section [*1]

(4×，H.E.)

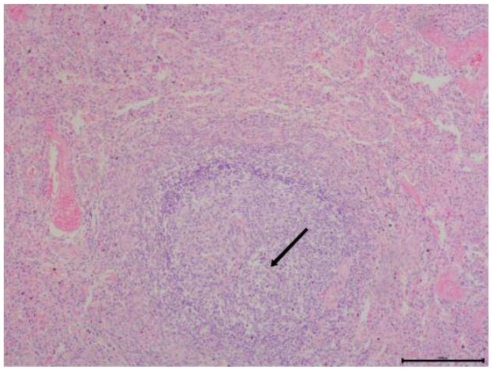

Canine spleen tissue section [*1]

(10×，H.E.)

"*1": Splenic nodule edema with loosely arranged lymphocytes

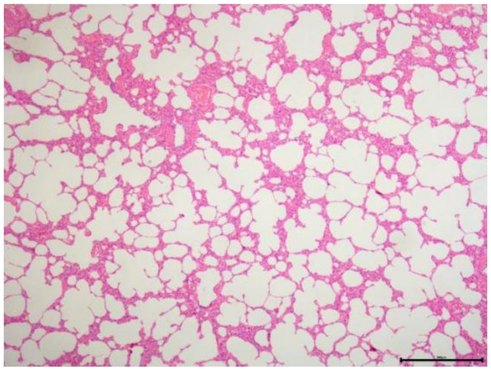

Canine lung tissue section

(4×，H.E.)

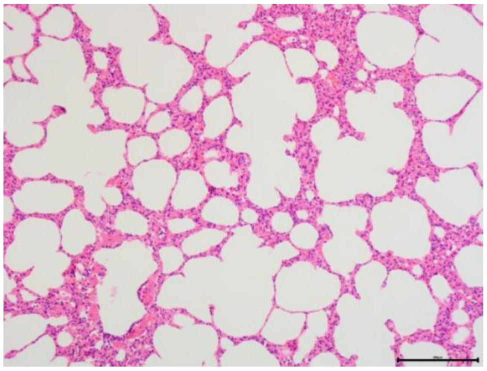

Canine lung tissue section

(10×，H.E.)

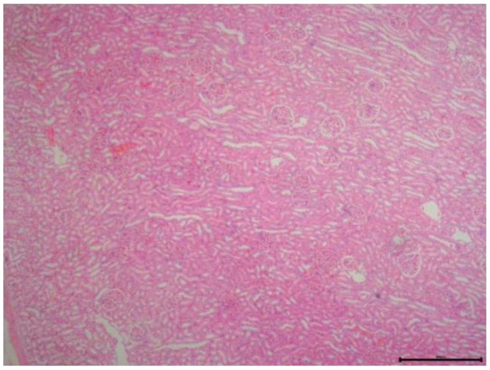

Canine kidney tissue section

(4×，H.E.)

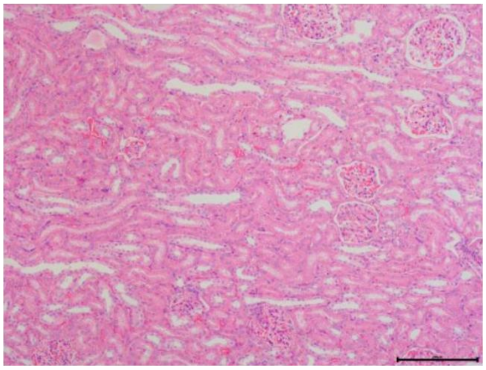

Canine kidney tissue section

(10×，H.E.)

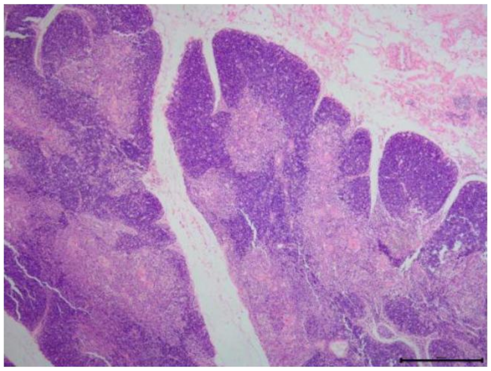

Canine thymus tissue section

(4×，H.E.)

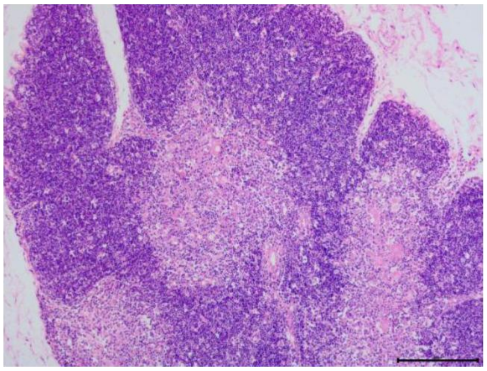

Canine thymus tissue section

(10×，H.E.)

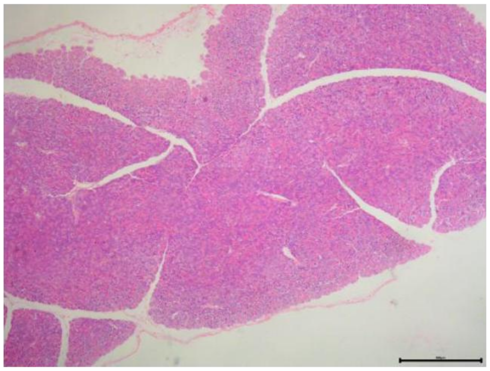

Canine pancreas tissue section

(4×，H.E.)

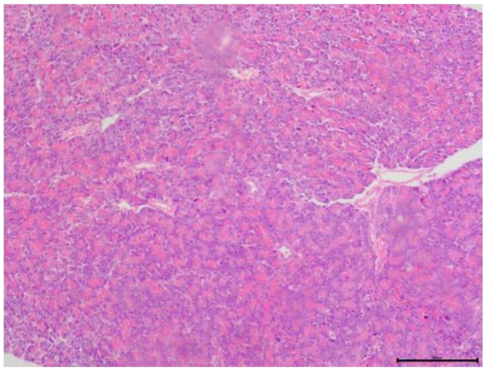

Canine pancreas tissue section

(10×，H.E.)

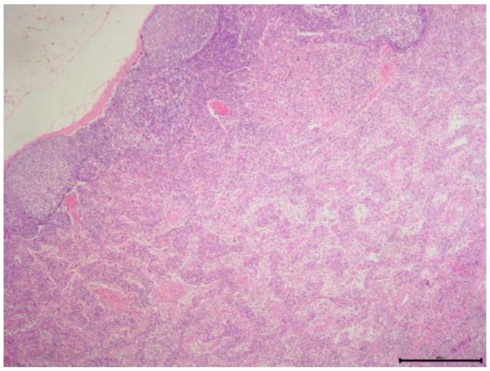

Canine lymph node tissue section

(4×，H.E.)

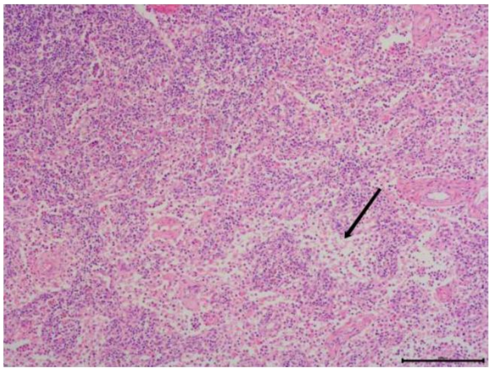

Canine lymph node tissue section*2

(10×，H.E.)

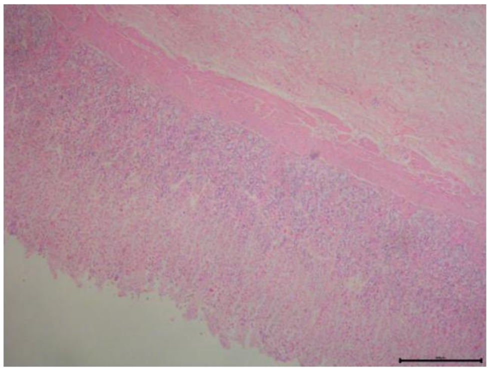

Canine stomach tissue section

(4×，H.E.)

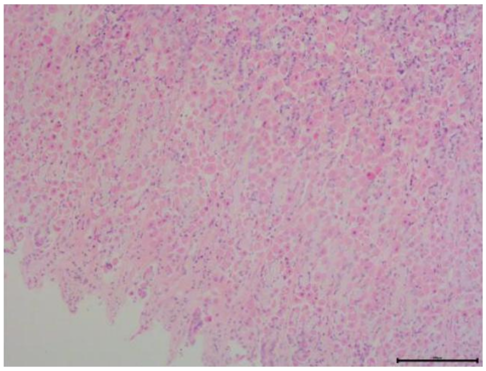

Canine stomach tissue section

(10×，H.E.)

“*2”: Lymph node edema

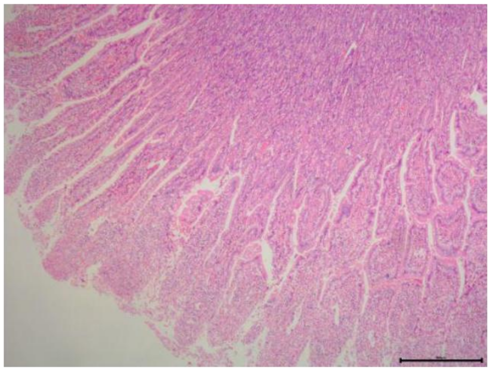

Canine duodenum tissue section

(4×，H.E.)

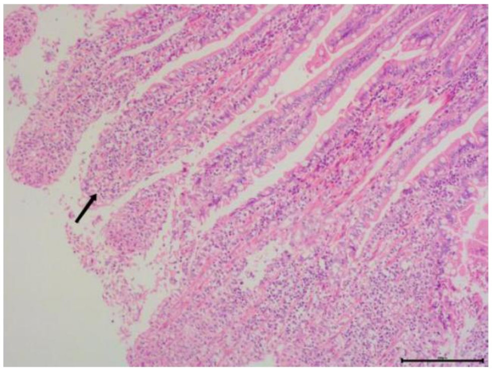

Canine duodenum tissue section*3

(10×，H.E.)

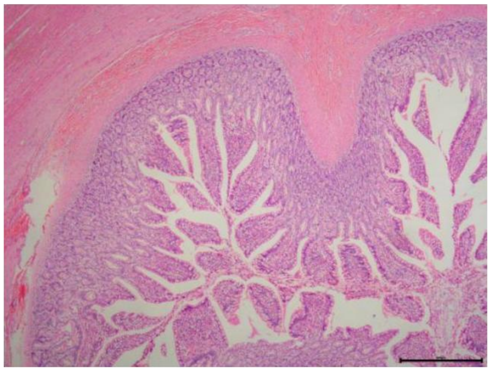

Canine ileum tissue section

(10×，H.E.)

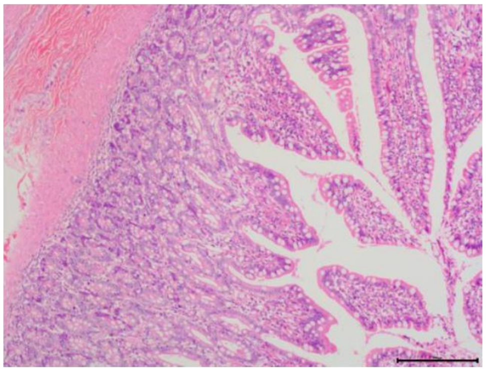

Canine ileum tissue section

(10×，H.E.)

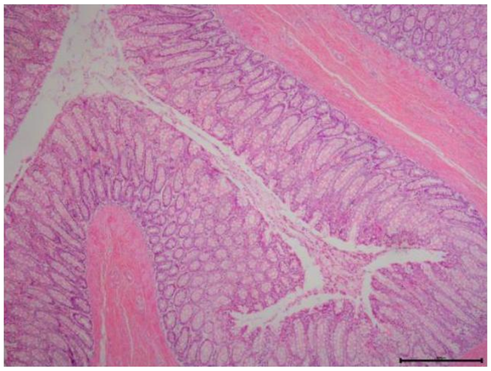

Canine rectum tissue section

(4×，H.E.)

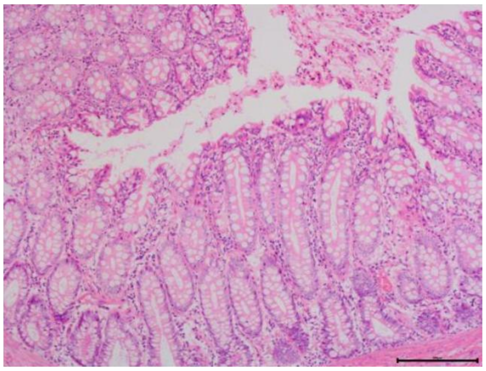

Canine rectum tissue section

(10×，H.E.)

"*3": Intestinal villus edema

5× dose group——Histopathological sections of Canine No. 24

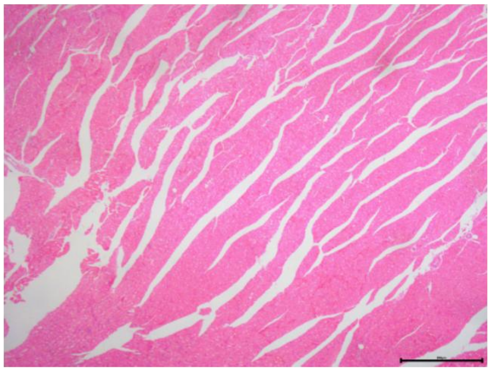

Canine myocardial tissue section(4×，H.E.)

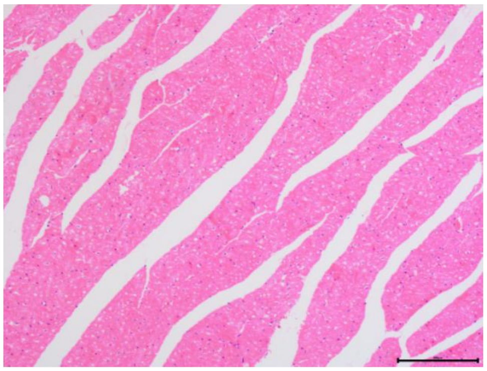

Canine myocardial tissue section(10×，H.E.)

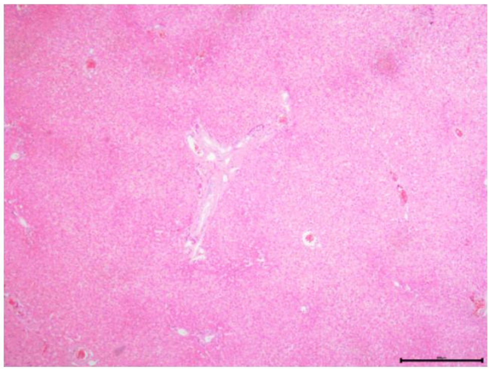

Canine liver tissue section

(4×，H.E.)

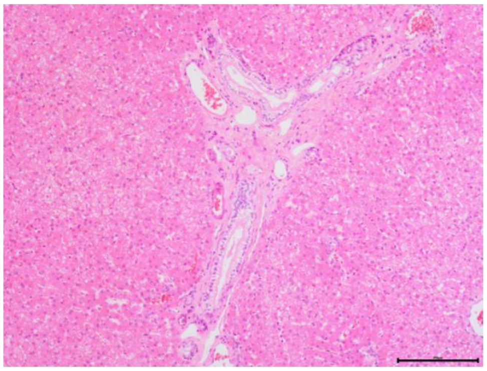

Canine liver tissue section

(10×，H.E.)

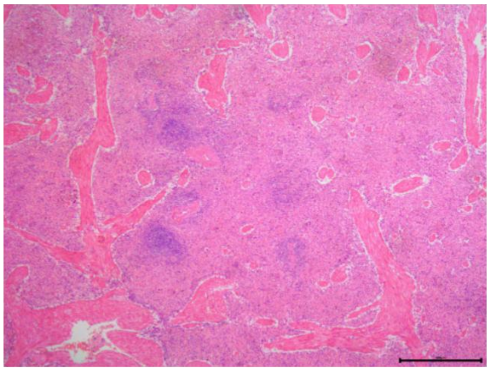

Canine spleen tissue section

(4×，H.E.)

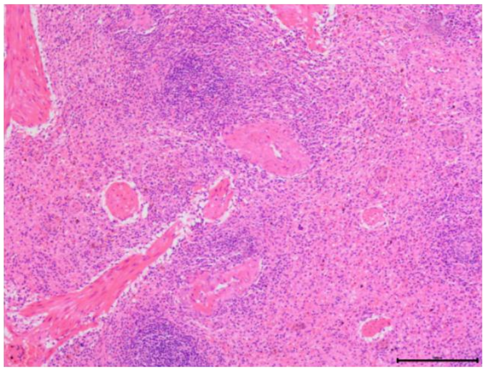

Canine spleen tissue section

(10×，H.E.)

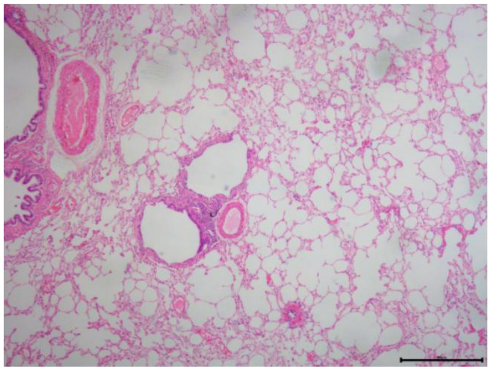

Canine lung tissue section

(4×，H.E.)

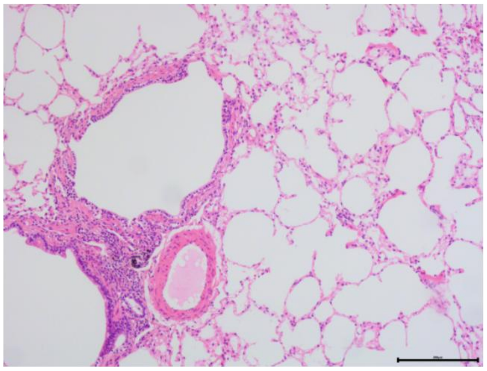

Canine lung tissue section

(10×，H.E.)

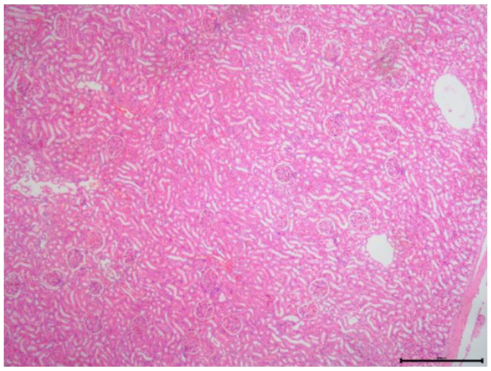

Canine kidney tissue section

(4×，H.E.)

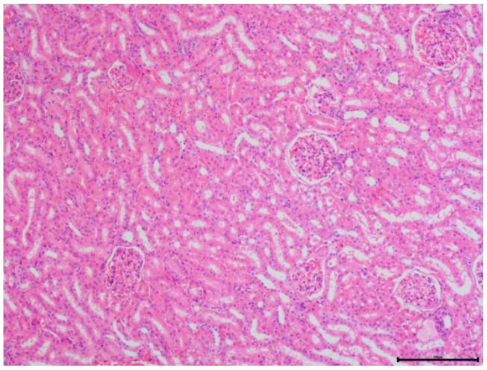

Canine kidney tissue section

(10×，H.E.)

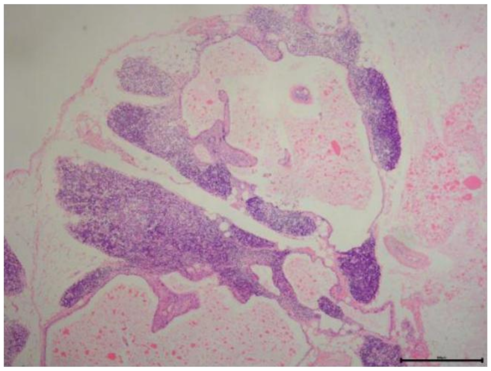

Canine thymus tissue section

(4×，H.E.)

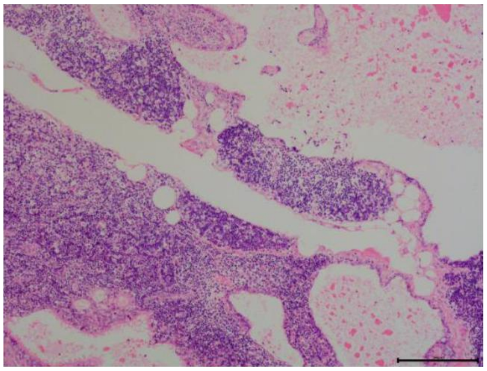

Canine thymus tissue section

(10×，H.E.)

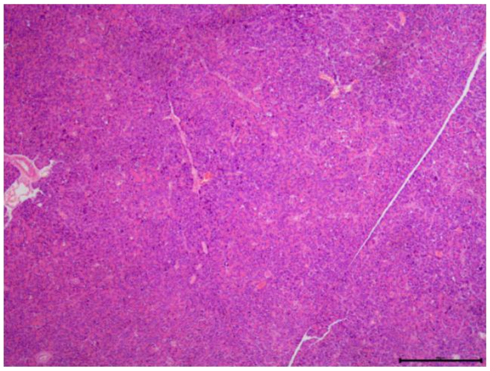

Canine pancreas tissue section

(4×，H.E.)

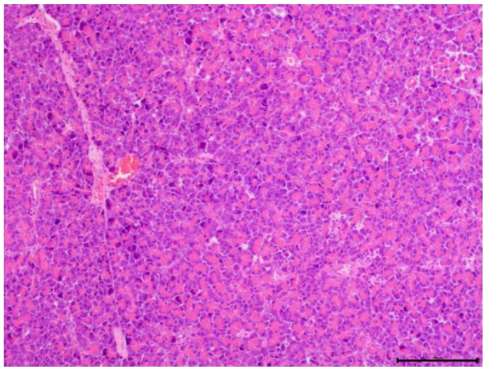

Canine pancreas tissue section

(10×，H.E.)

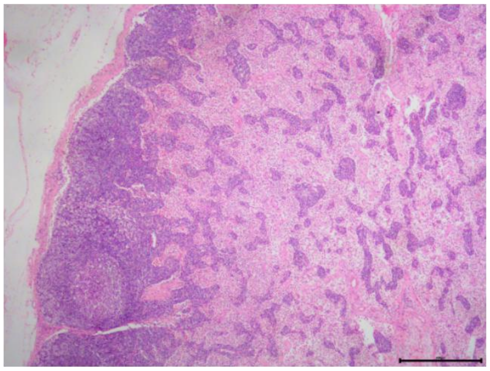

Canine lymph node tissue section

(4×，H.E.)

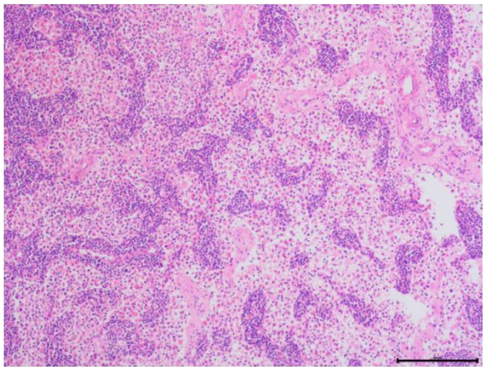

Canine lymph node tissue section

(10×，H.E.)

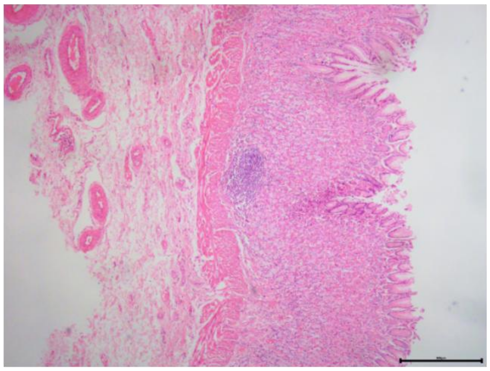

Canine stomach tissue section

(4×，H.E.)

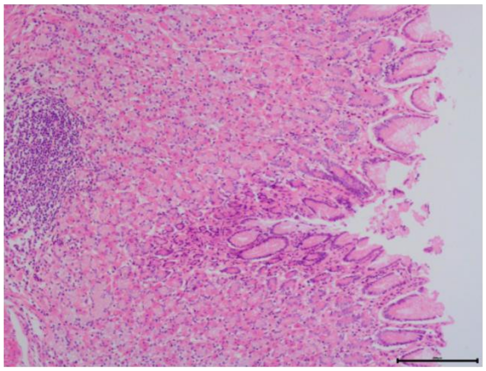

Canine stomach tissue section

(10×，H.E.)

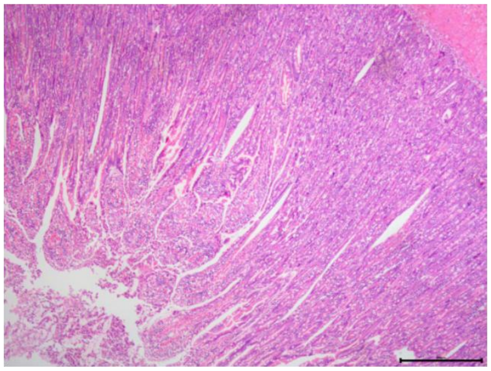

Canine duodenum tissue section

(4×， H.E.)

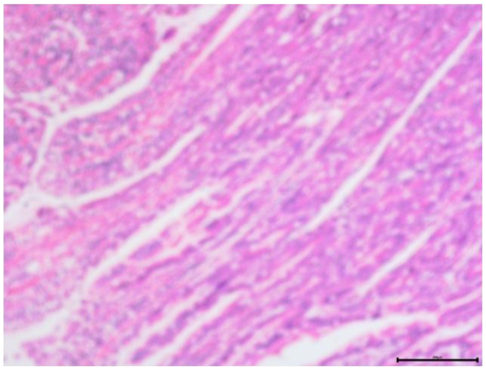

Canine duodenum tissue section

(10×， H.E.)

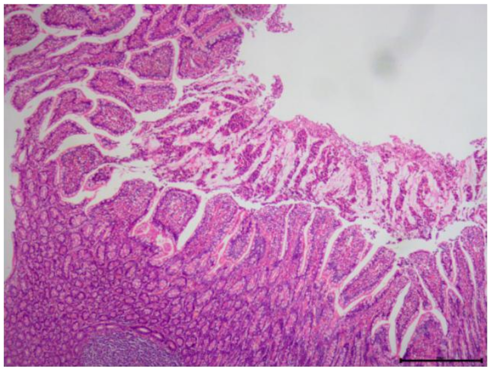

Canine ileum tissue section

(10×， H.E.)

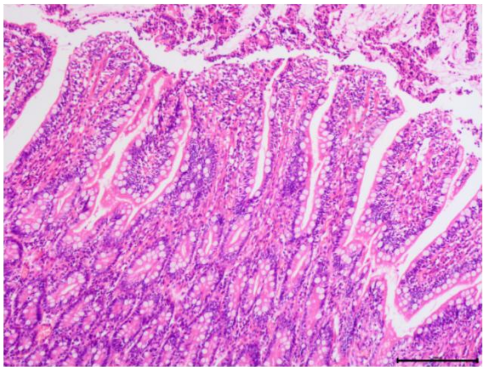

Canine ileum tissue section

(10×， H.E.)

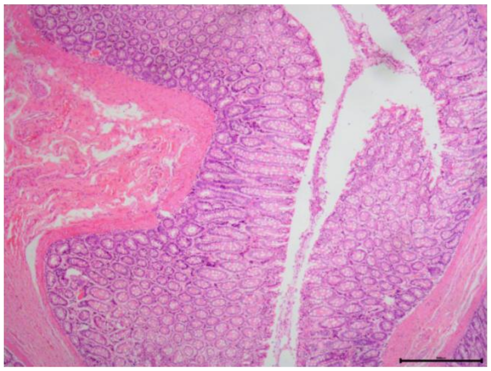

Canine rectum tissue section

(4×， H.E.)

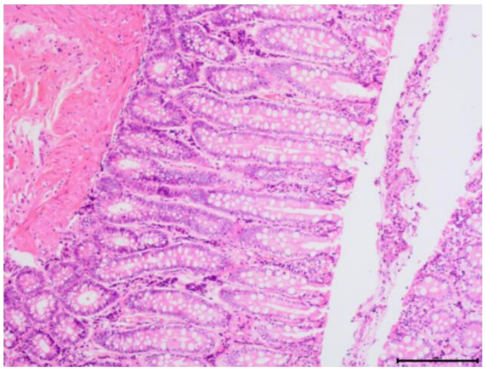

Canine rectum tissue section

(10×， H.E.)

5× dose group——Histopathological sections of Canine No. 25

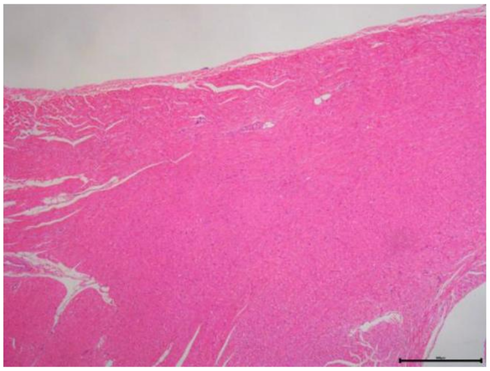

Canine myocardial tissue section(4×，H.E.)

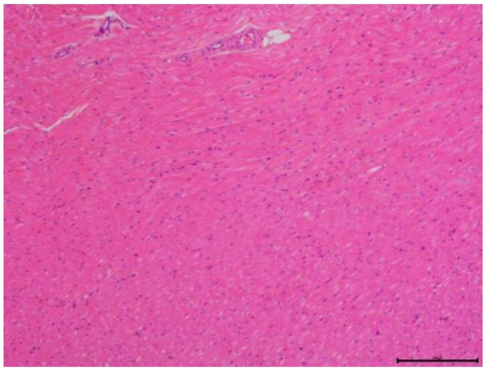

Canine myocardial tissue section(10×，H.E.)

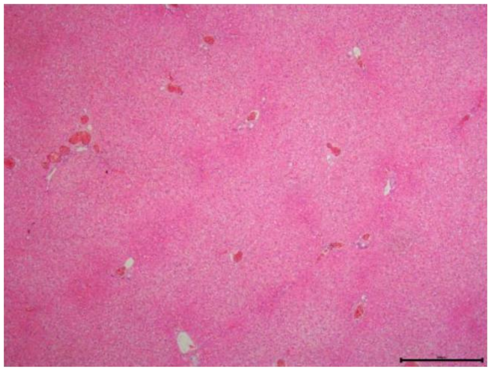

Canine liver tissue section

(4×，H.E.)

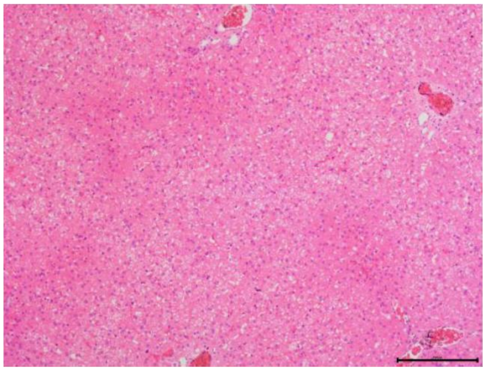

Canine liver tissue section

(10×，H.E.)

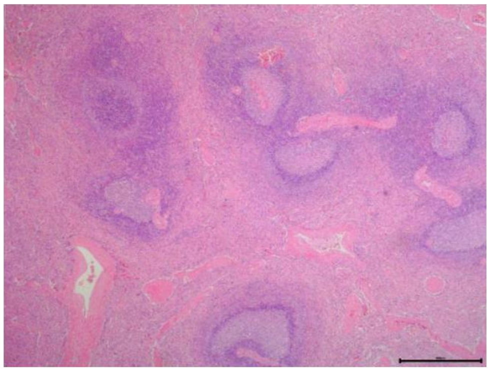

Canine spleen tissue section

(4×，H.E.)

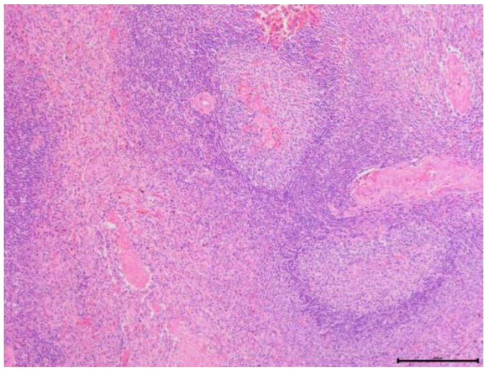

Canine spleen tissue section

(10×，H.E.)

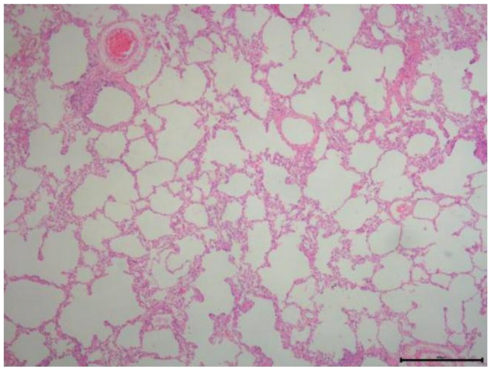

Canine lung tissue section

(4×，H.E.)

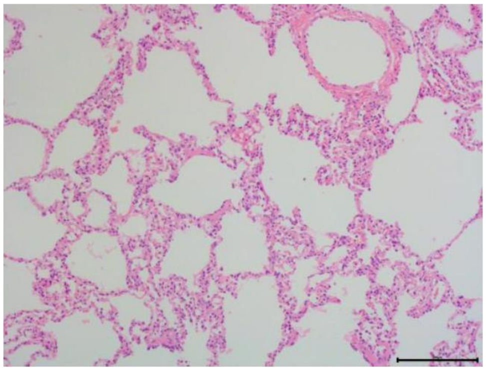

Canine lung tissue section

(10×，H.E.)

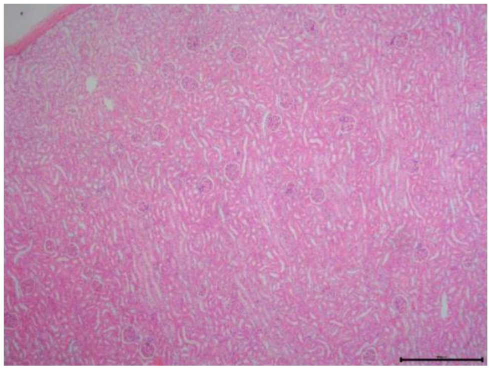

Canine kidney tissue section

(4×，H.E.)

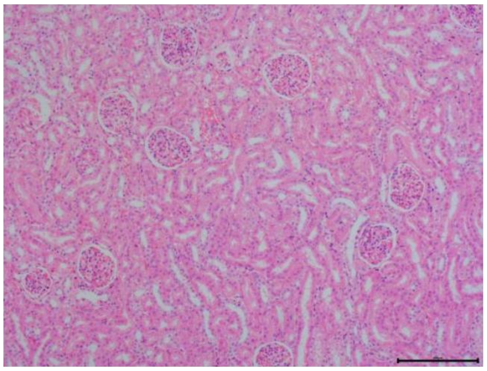

Canine kidney tissue section

(10×，H.E.)

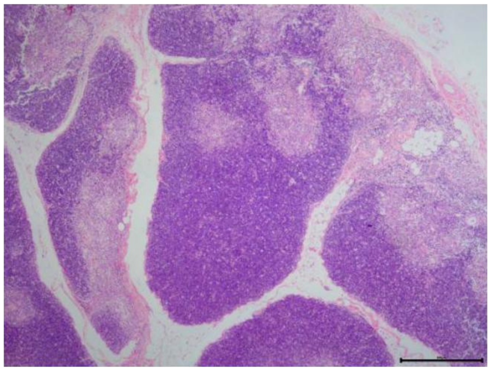

Canine thymus tissue section

(4×，H.E.)

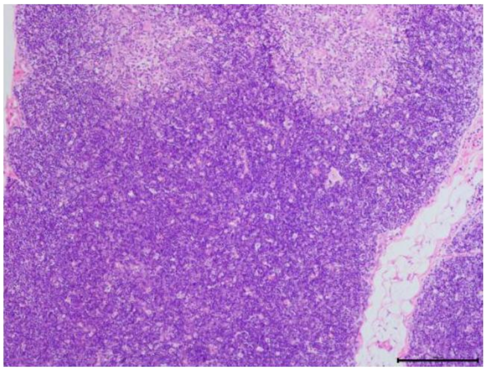

Canine thymus tissue section

(10×，H.E.)

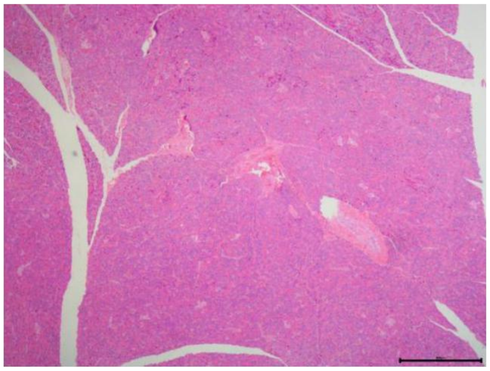

Canine pancreas tissue section

(4×，H.E.)

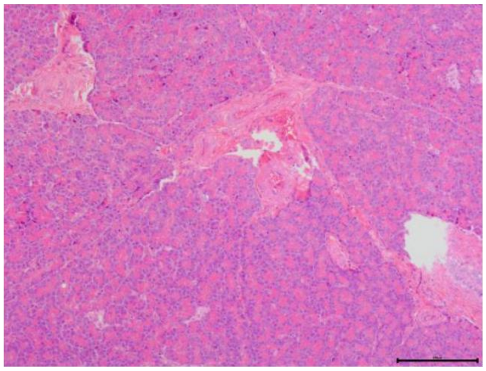

Canine pancreas tissue section

(10×，H.E.)

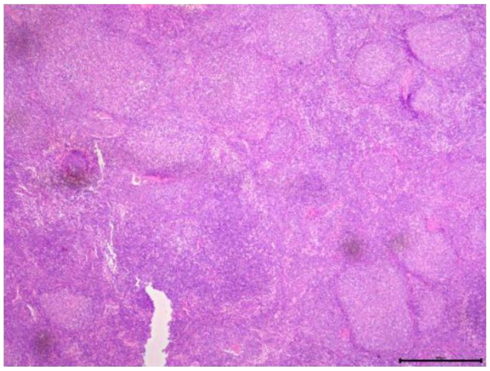

Canine lymph node tissue section

(4×，H.E.)

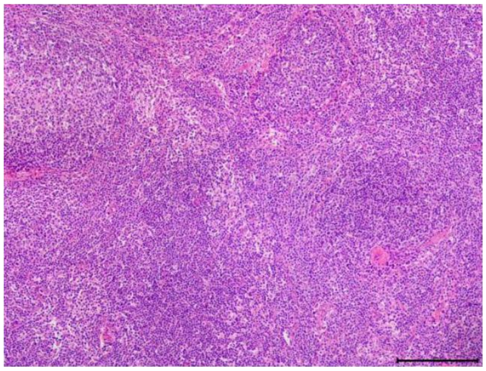

Canine lymph node tissue section

(10×，H.E.)

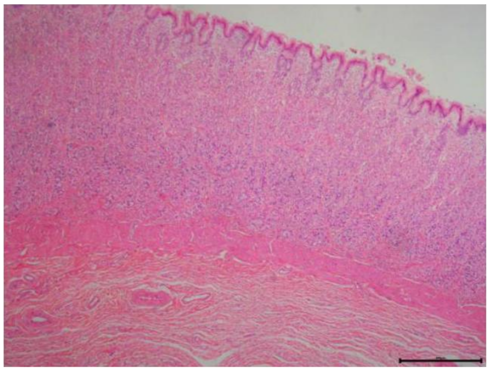

Canine stomach tissue section

(4×，H.E.)

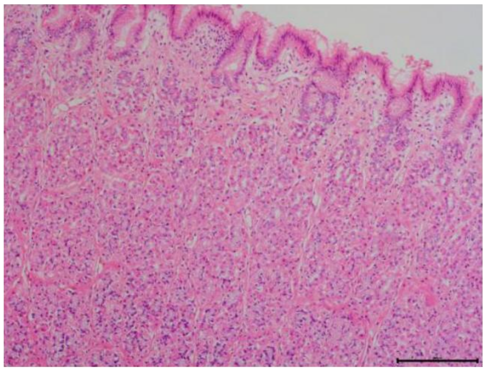

Canine stomach tissue section

(10×，H.E.)

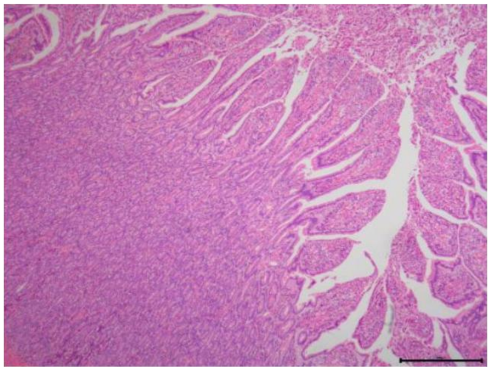

Canine duodenum tissue section

(4×， H.E.)

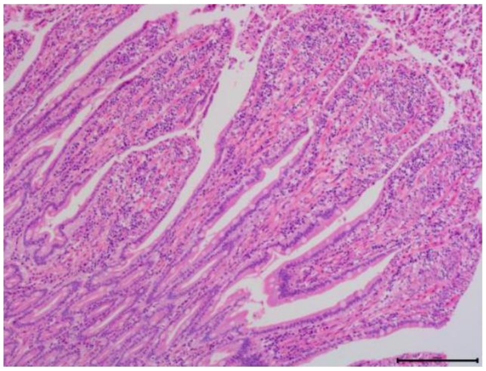

Canine duodenum tissue section

(10×， H.E.)

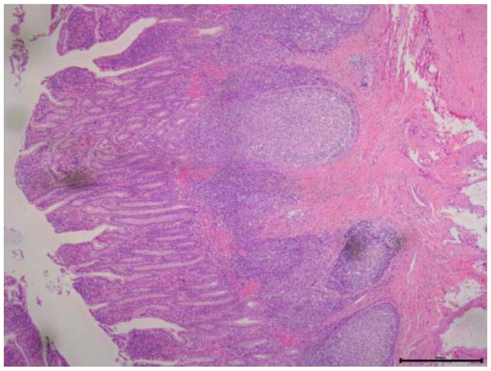

Canine ileum tissue section

(10×， H.E.)

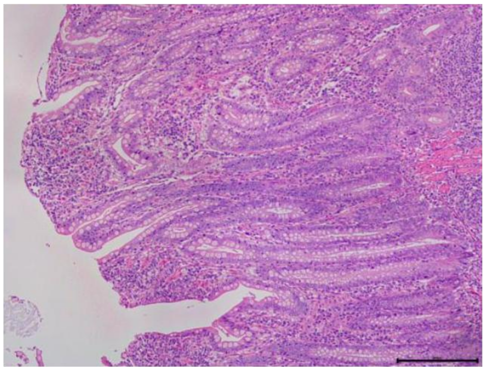

Canine ileum tissue section

(10×， H.E.)

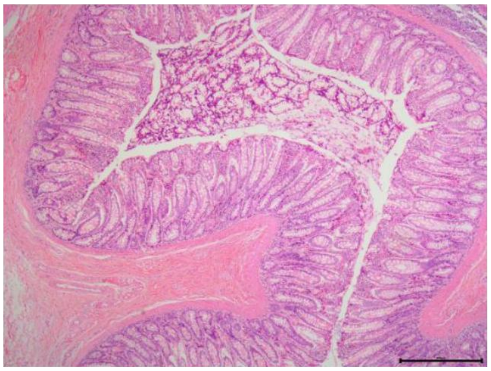

Canine rectum tissue section

(4×， H.E.)

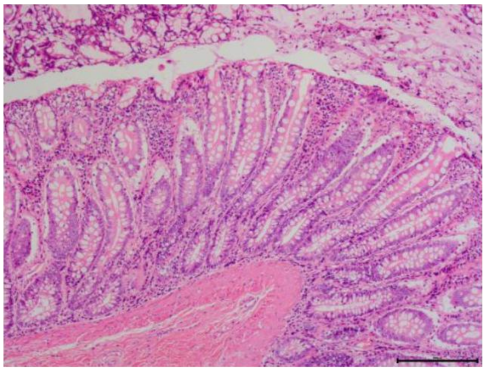

Canine rectum tissue section

(10×， H.E.)

5× dose group——Histopathological sections of Canine No. 26

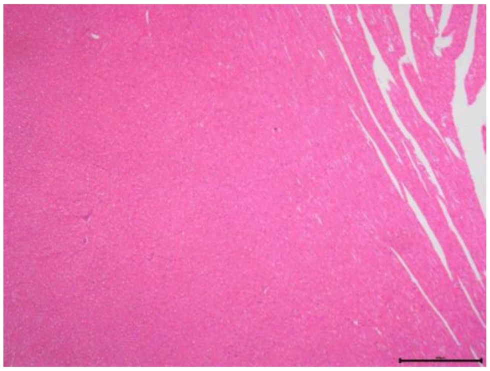

Canine myocardial tissue section(4×，H.E.)

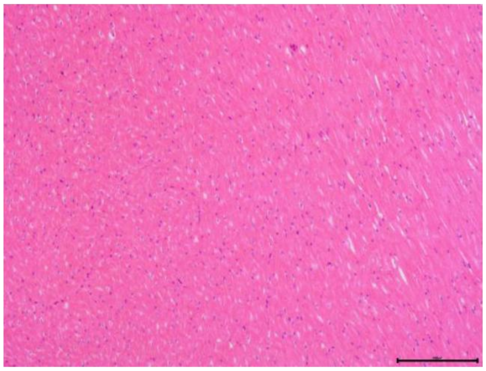

Canine myocardial tissue section(10×，H.E.)

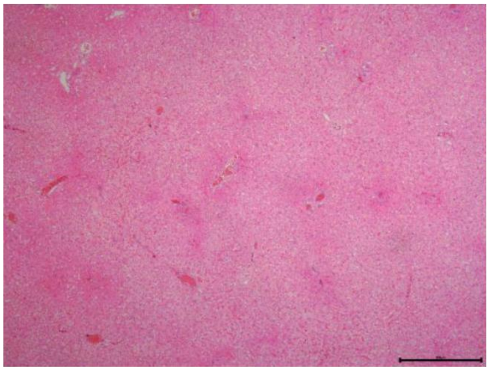

Canine liver tissue section

(4×，H.E.)

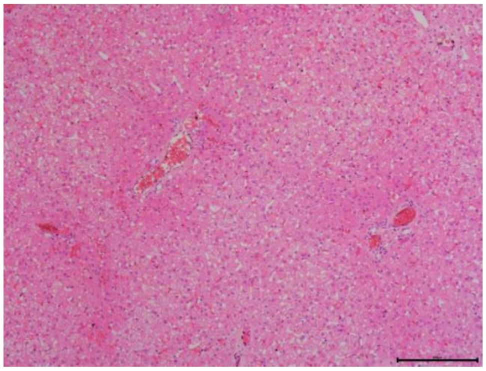

Canine liver tissue section

(10×，H.E.)

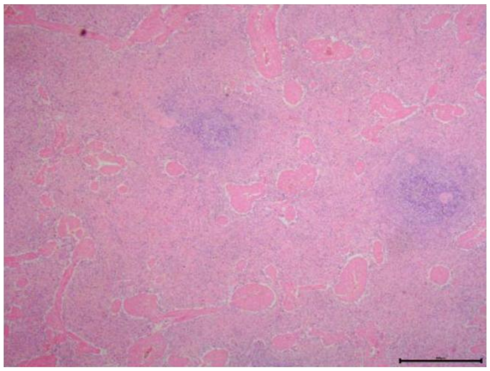

Canine spleen tissue section

(4×，H.E.)

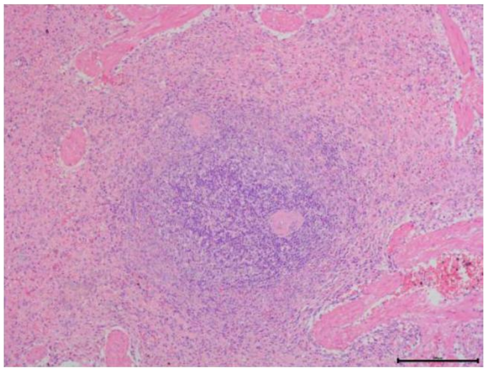

Canine spleen tissue section

(10×，H.E.)

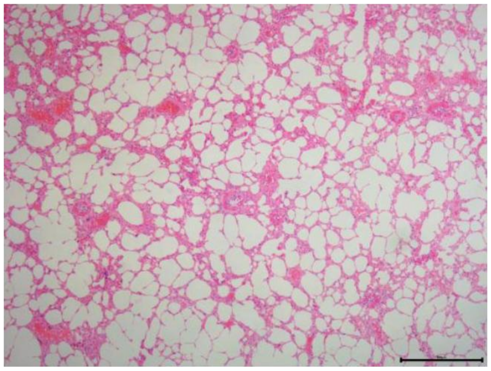

Canine lung tissue section

(4×，H.E.)

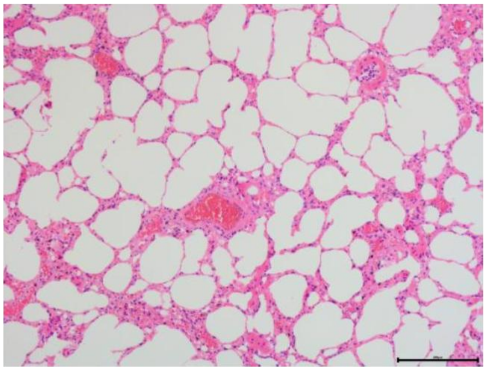

Canine lung tissue section

(10×，H.E.)

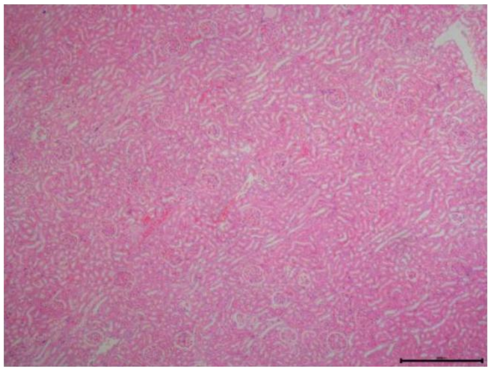

Canine kidney tissue section

(4×，H.E.)

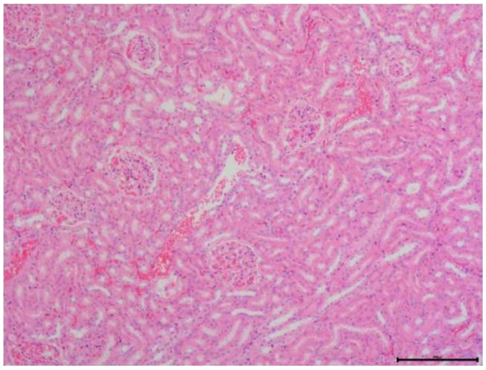

Canine kidney tissue section

(10×，H.E.)

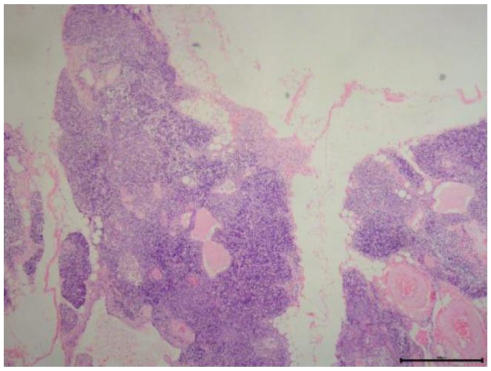

Canine thymus tissue section

(4×，H.E.)

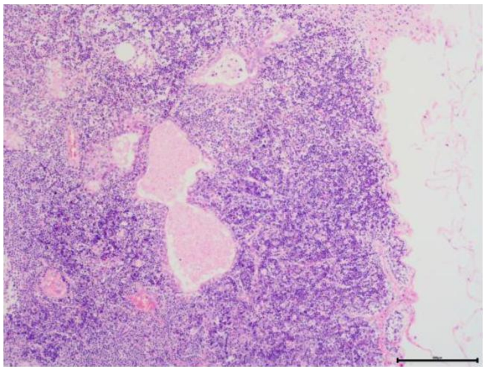

Canine thymus tissue section

(10×，H.E.)

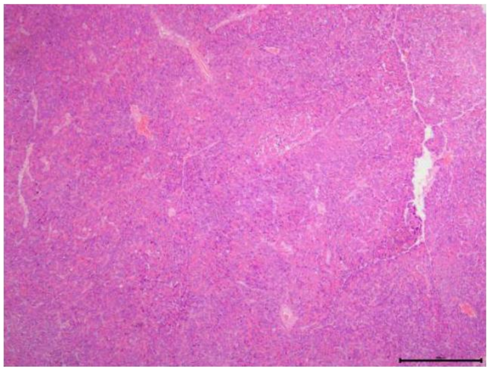

Canine pancreas tissue section

(4×，H.E.)

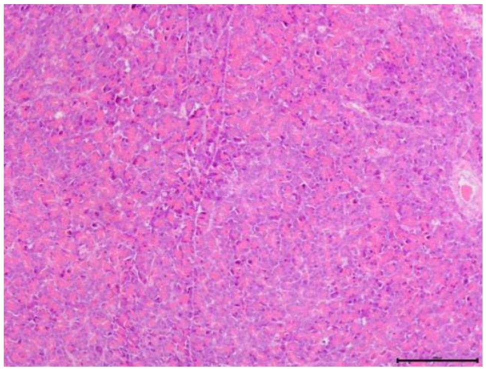

Canine pancreas tissue section

(10×，H.E.)

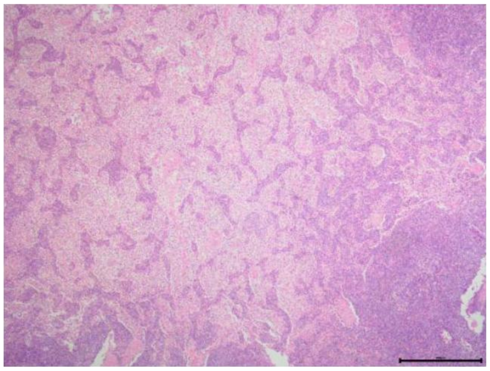

Canine lymph node tissue section

(4×，H.E.)

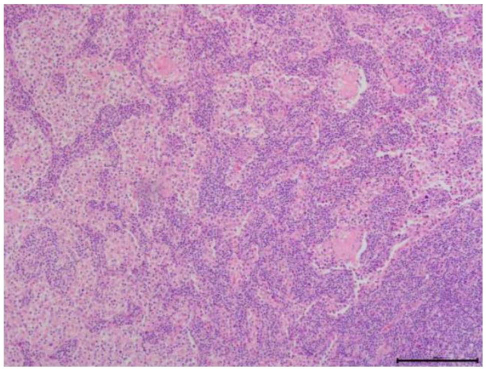

Canine lymph node tissue section

(10×，H.E.)

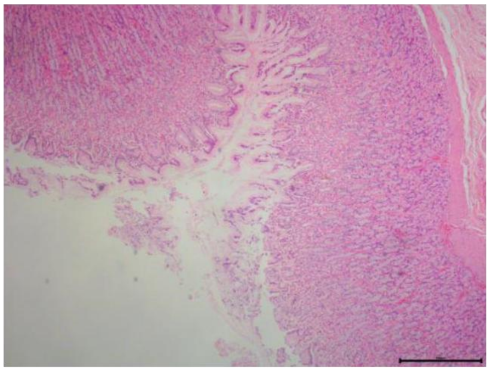

Canine stomach tissue section

(4×，H.E.)

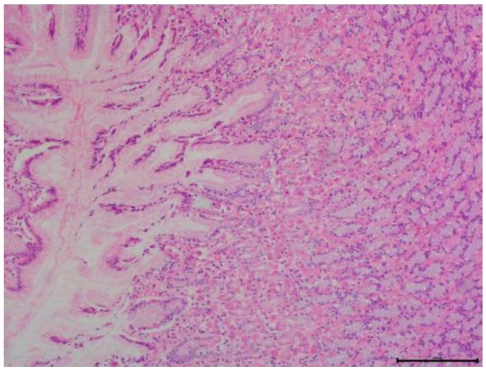

Canine stomach tissue section

(10×，H.E.)

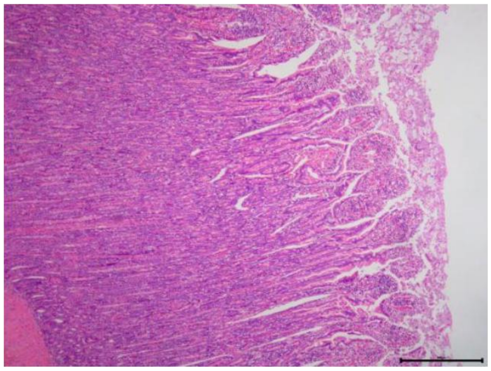

Canine duodenum tissue section

(4×， H.E.)

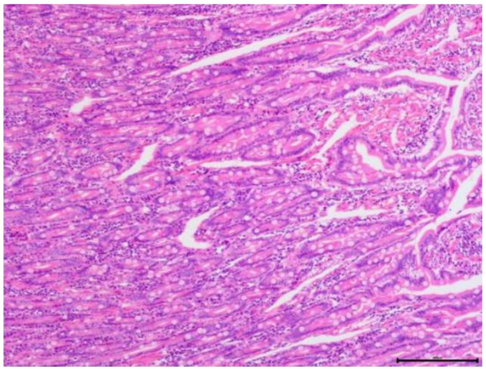

Canine duodenum tissue section

(10×， H.E.)

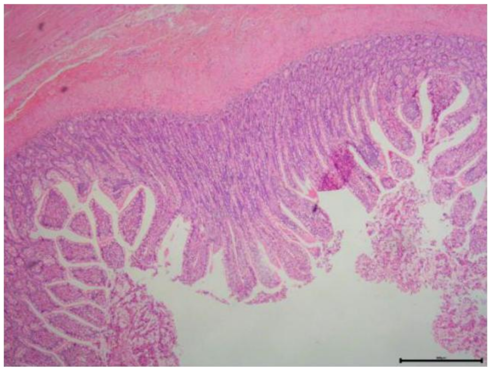

Canine ileum tissue section

(10×， H.E.)

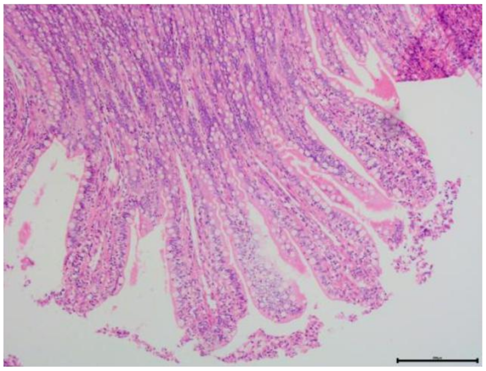

Canine ileum tissue section

(10×， H.E.)

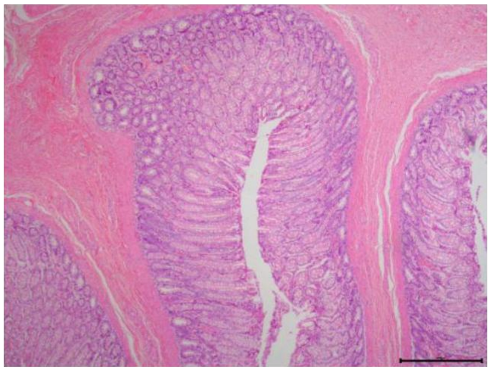

Canine rectum tissue section

(4×， H.E.)

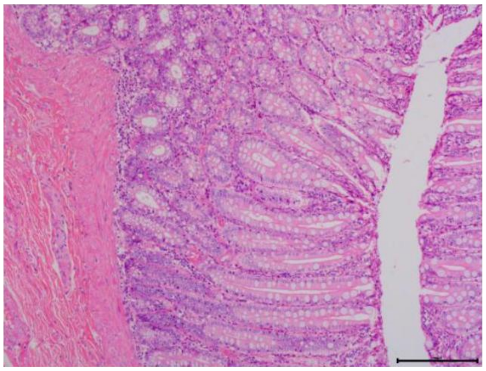

Canine rectum tissue section

(10×， H.E.)

5× dose group——Histopathological sections of Canine No. 31

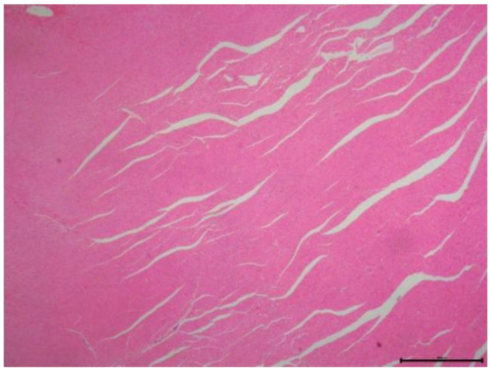

Canine myocardial tissue section(4×，H.E.)

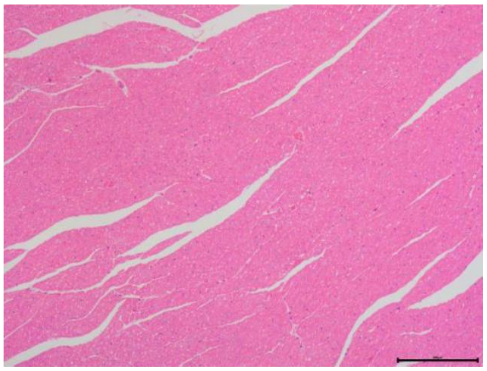

Canine myocardial tissue section(10×，H.E.)

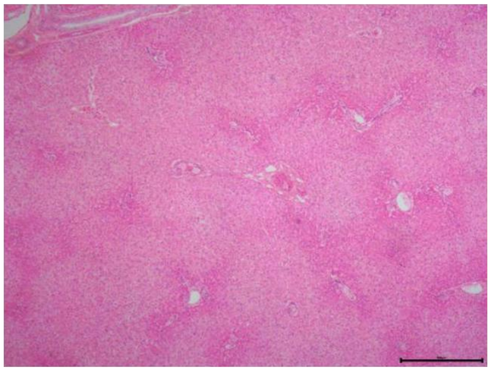

Canine liver tissue section

(4×，H.E.)

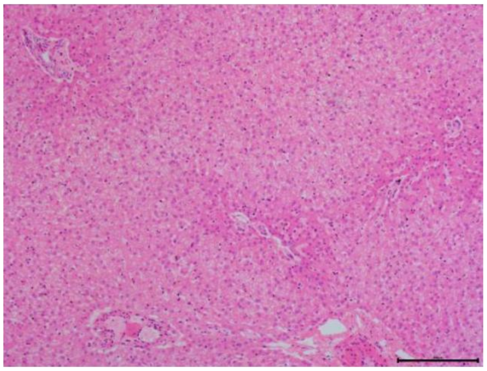

Canine liver tissue section

(10×，H.E.)

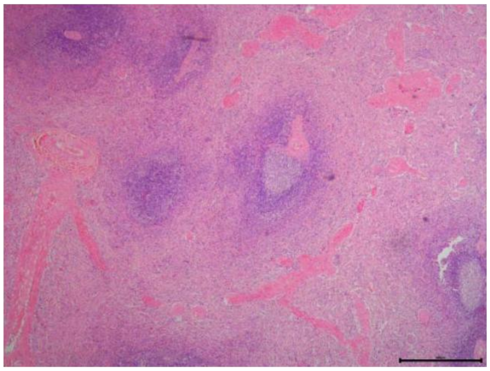

Canine spleen tissue section

(4×，H.E.)

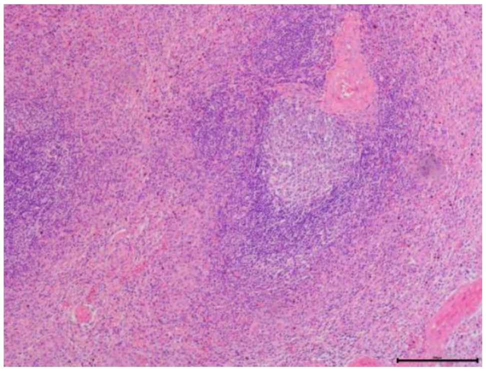

Canine spleen tissue section

(10×，H.E.)

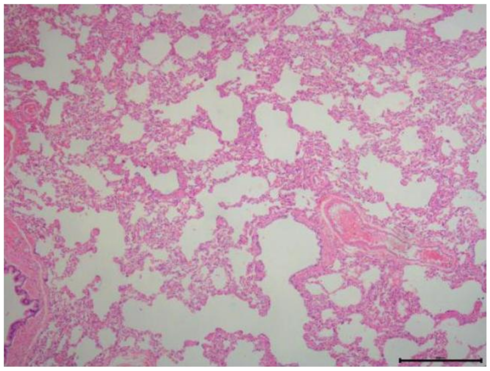

Canine lung tissue section

(4×，H.E.)

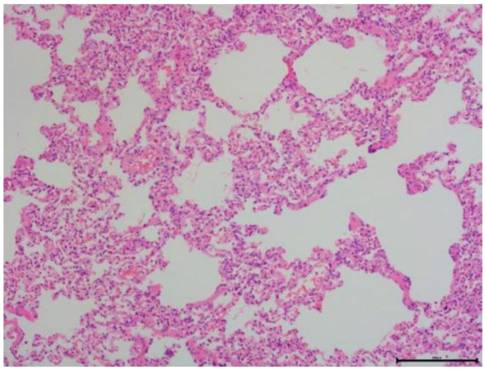

Canine lung tissue section

(10×，H.E.)

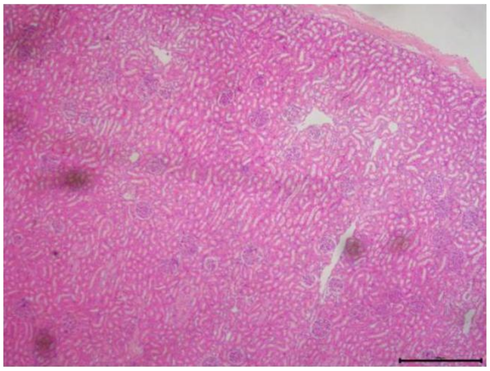

Canine kidney tissue section

(4×，H.E.)

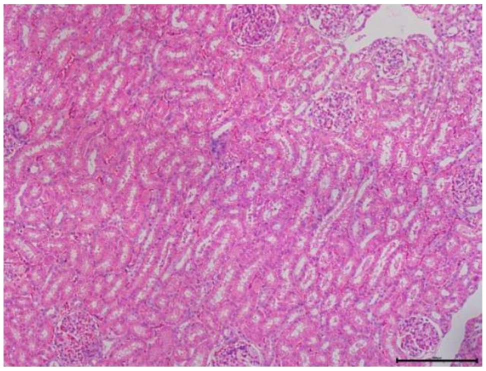

Canine kidney tissue section

(10×，H.E.)

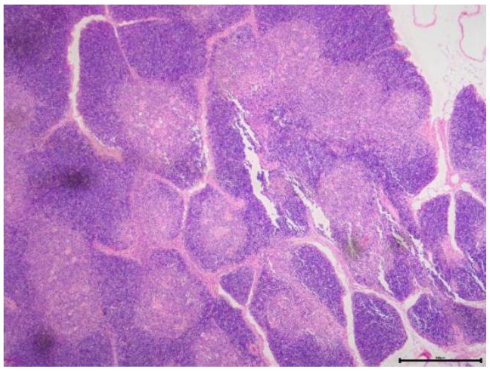

Canine thymus tissue section

(4×，H.E.)

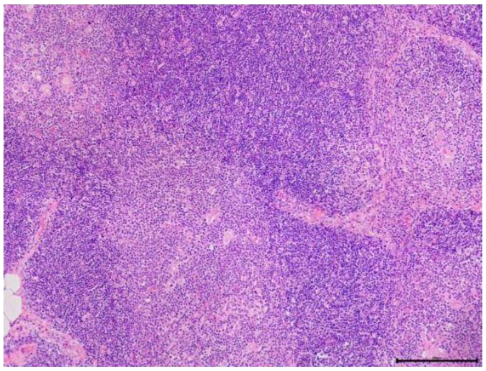

Canine thymus tissue section

(10×，H.E.)

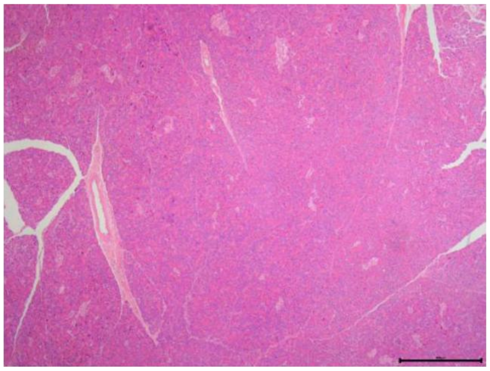

Canine pancreas tissue section

(4×，H.E.)

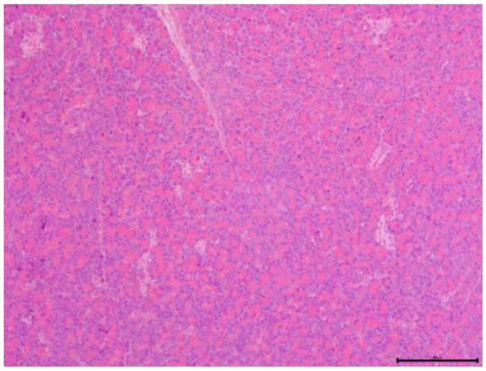

Canine pancreas tissue section

(10×，H.E.)

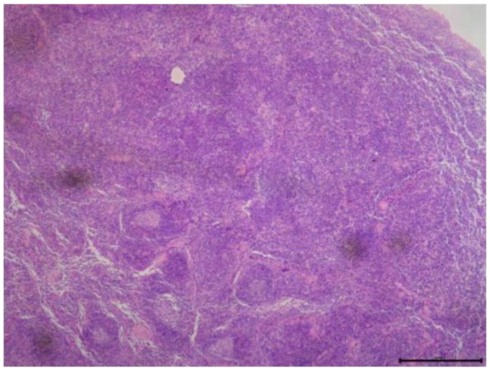

Canine lymph node tissue section

(4×，H.E.)

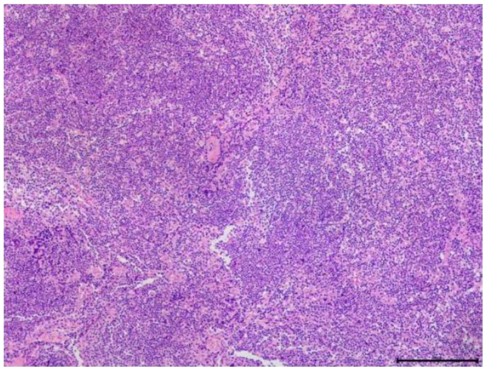

Canine lymph node tissue section

(10×，H.E.)

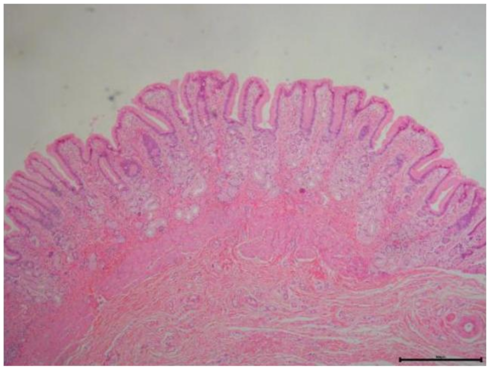

Canine stomach tissue section

(4×，H.E.)

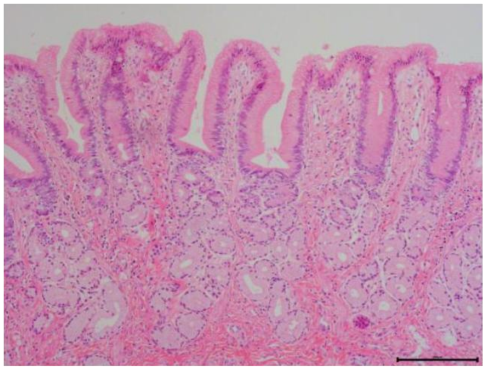

Canine stomach tissue section

(10×，H.E.)

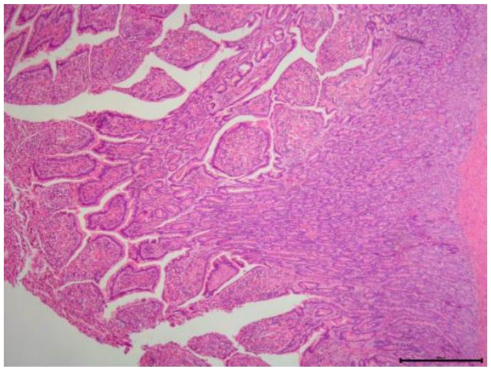

Canine duodenum tissue section

(4×， H.E.)

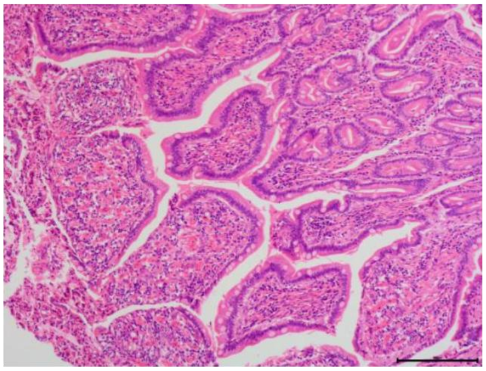

Canine duodenum tissue section

(10×， H.E.)

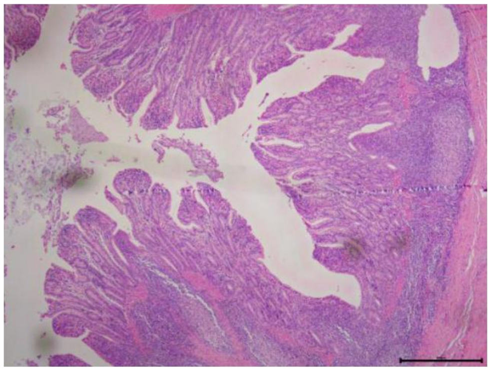

Canine ileum tissue section

(10×， H.E.)

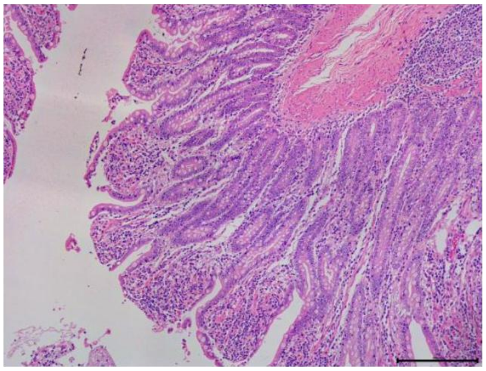

Canine ileum tissue section

(10×， H.E.)

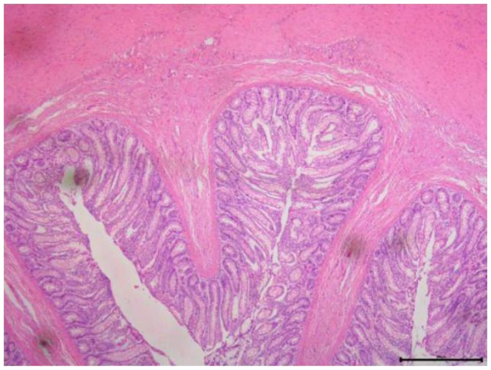

Canine rectum tissue section

(4×， H.E.)

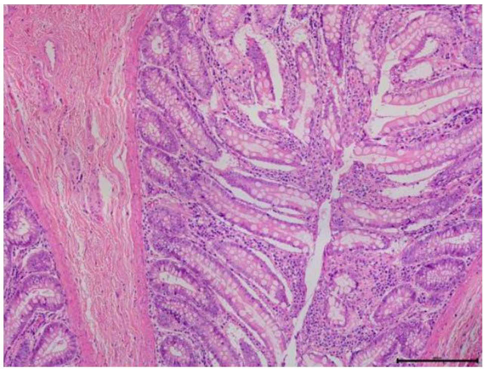

Canine rectum tissue section

(10×， H.E.)

Saline control group——Histopathological sections of Canine No. 02

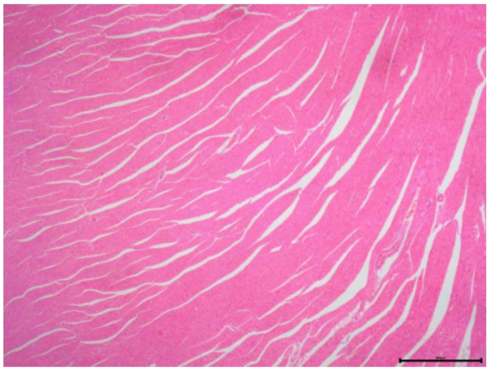

Canine myocardial tissue section(4×，H.E.)

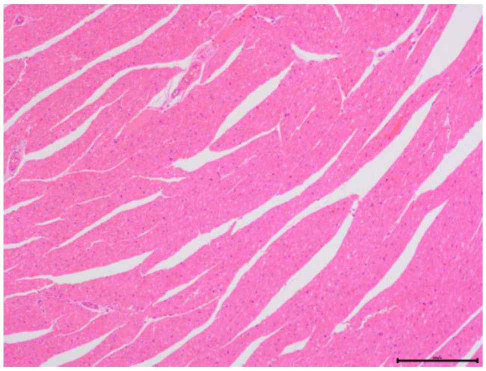

Canine myocardial tissue section(10×，H.E.)

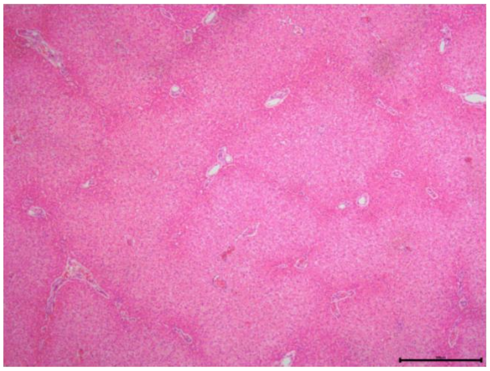

Canine liver tissue section

(4×，H.E.)

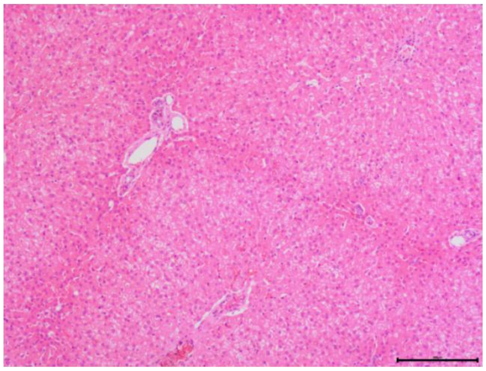

Canine liver tissue section

(10×，H.E.)

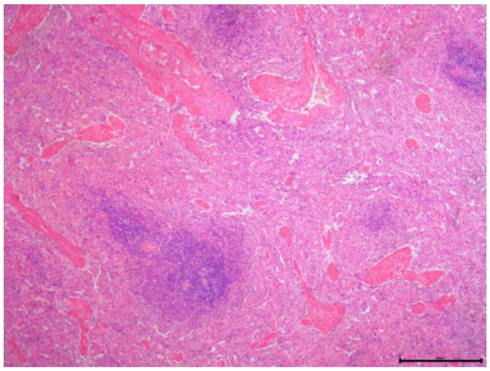

Canine spleen tissue section

(4×，H.E.)

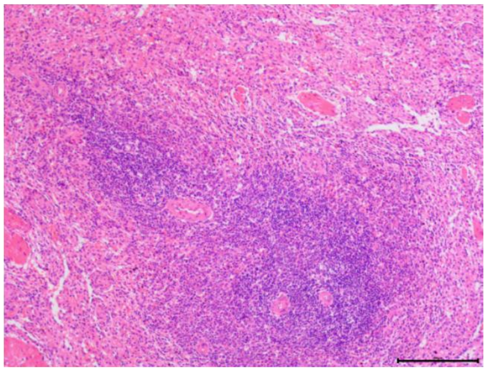

Canine spleen tissue section

(10×，H.E.)

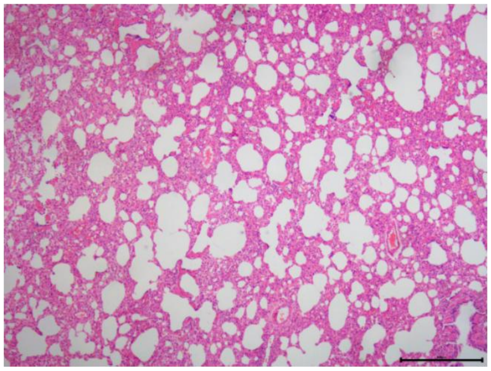

Canine lung tissue section

(4×，H.E.)

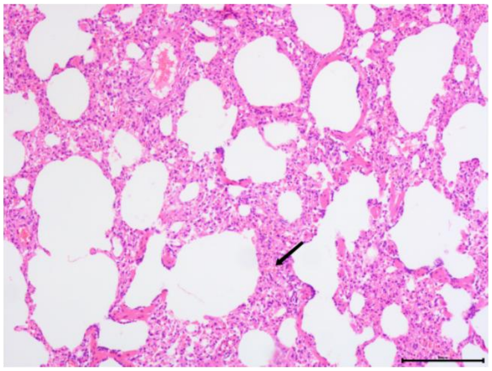

Canine lung tissue section

(10×，H.E.)

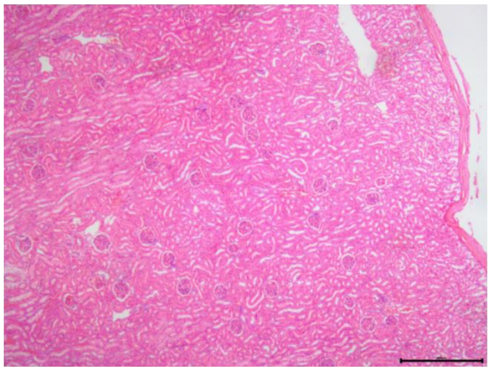

Canine kidney tissue section

(4×，H.E.)

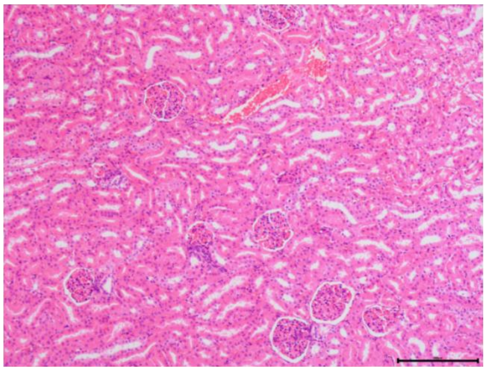

Canine kidney tissue section

(10×，H.E.)

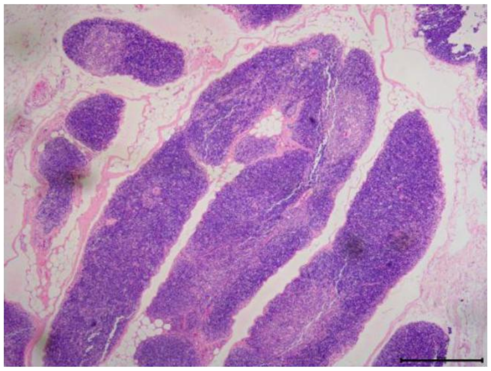

Canine thymus tissue section

(4×，H.E.)

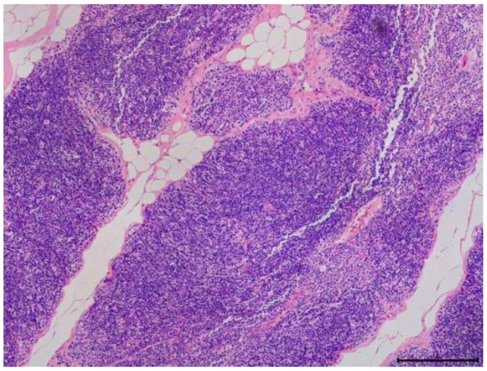

Canine thymus tissue section

(10×，H.E.)

"*1": Focal pulmonary interstitial hyperplasia

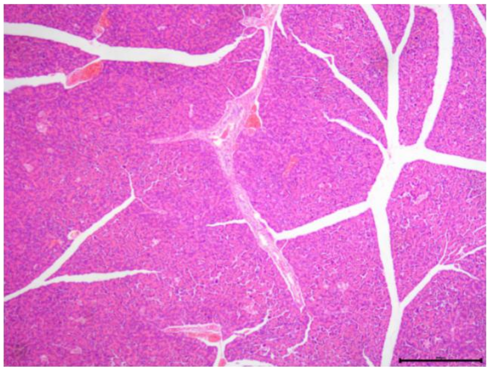

Canine pancreas tissue section

(4×，H.E.)

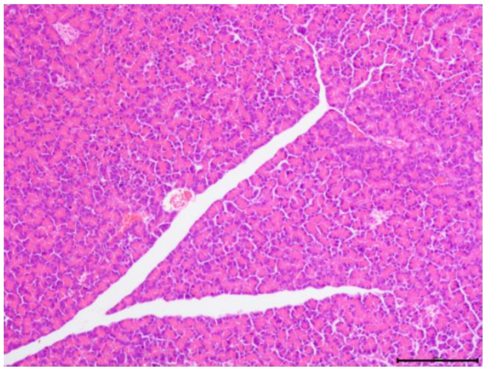

Canine pancreas tissue section

(10×，H.E.)

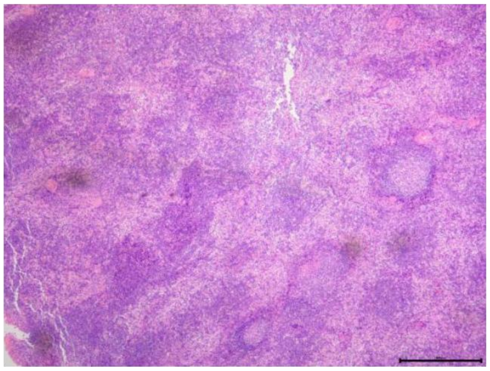

Canine lymph node tissue section

(4×，H.E.)

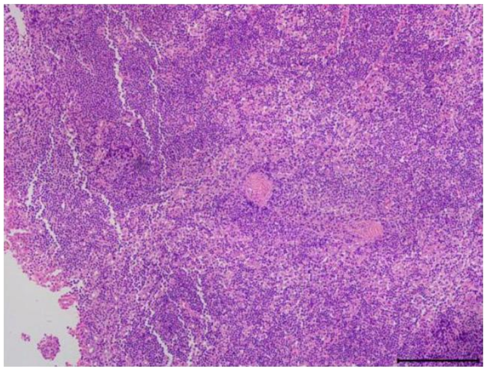

Canine lymph node tissue section

(10×，H.E.)

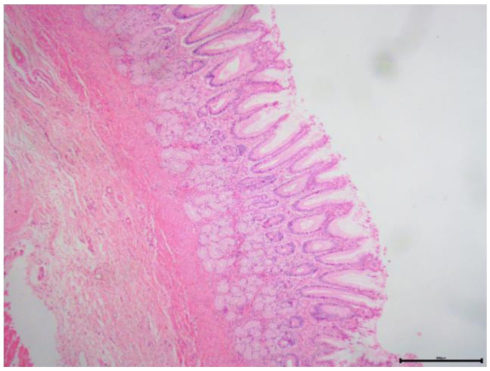

Canine stomach tissue section

(4×，H.E.)

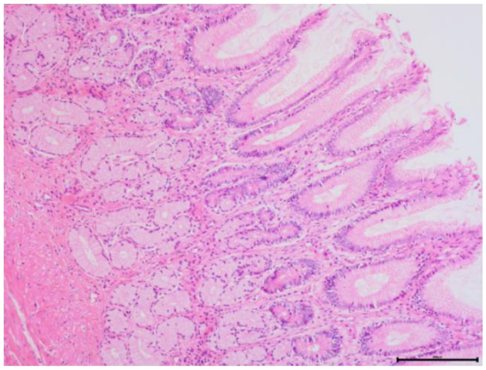

Canine stomach tissue section

(10×，H.E.)

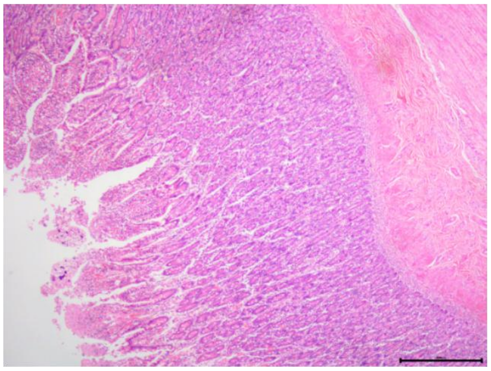

Canine duodenum tissue section

(4×， H.E.)

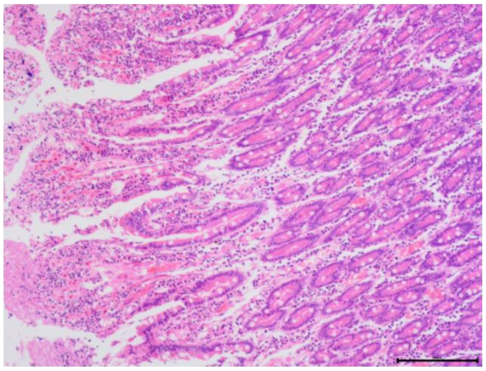

Canine duodenum tissue section

(10×， H.E.)

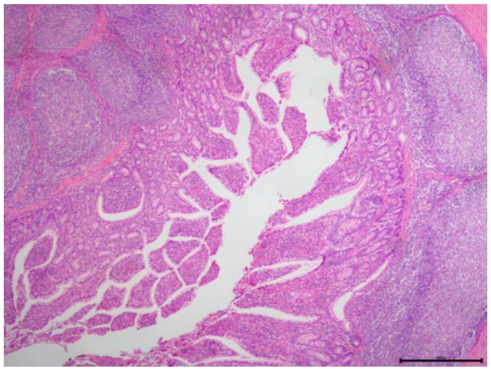

Canine ileum tissue section

(10×， H.E.)

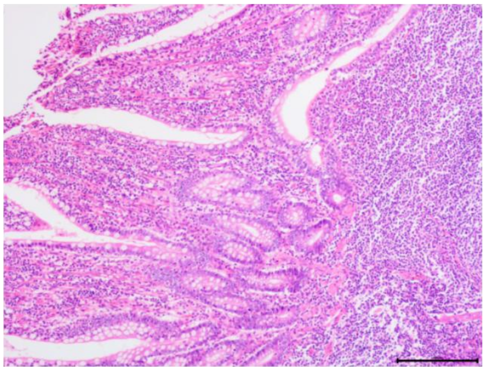

Canine ileum tissue section

(10×， H.E.)

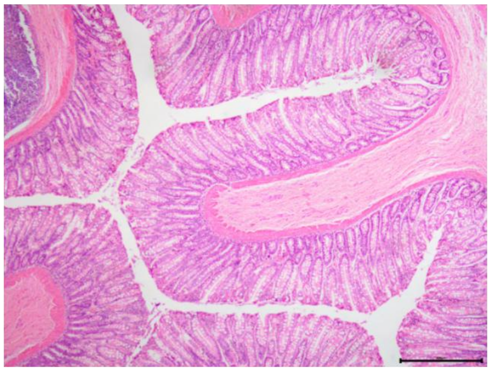

Canine rectum tissue section

(4×， H.E.)

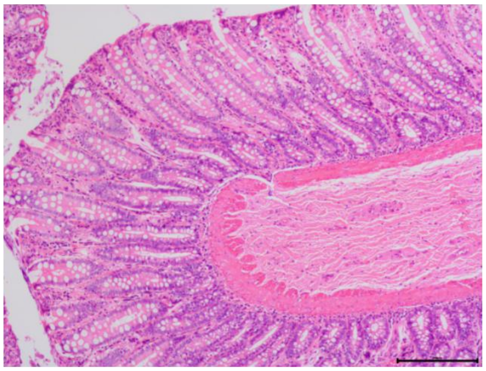

Canine rectum tissue section

(10×， H.E.)

Saline control group——Histopathological sections of Canine No. 05

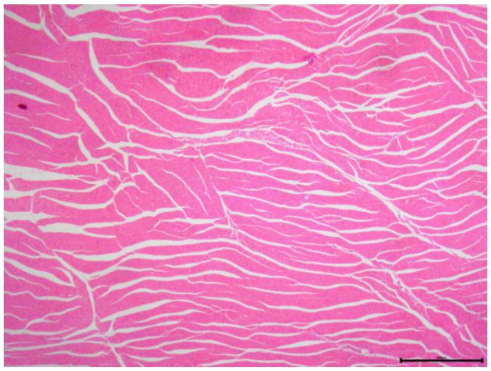

Canine myocardial tissue section(4×， H.E.)

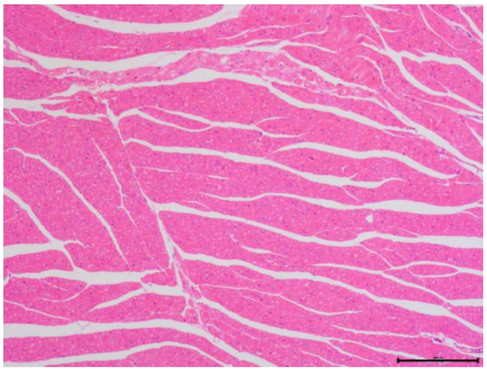

Canine myocardial tissue section(10×， H.E.)

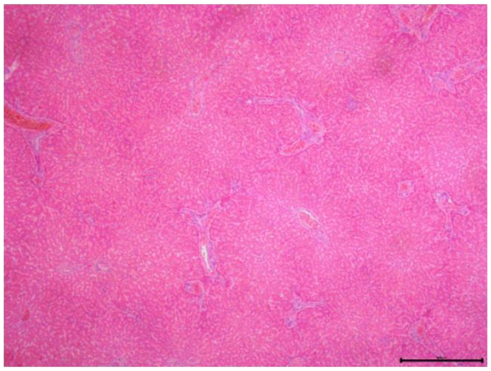

Canine liver tissue section

(4×， H.E.)

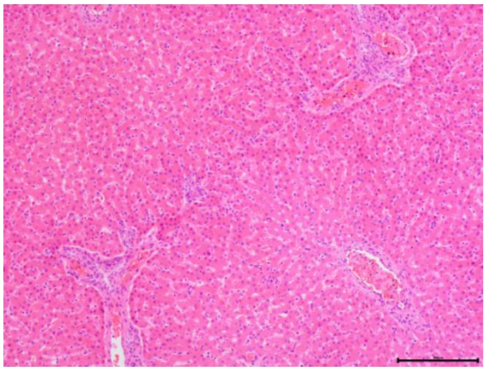

Canine liver tissue section

(10×， H.E.)

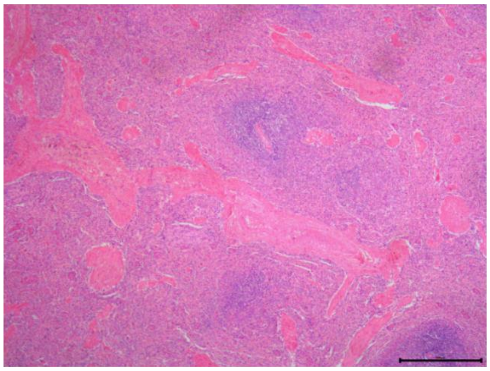

Canine spleen tissue section

(4×， H.E.)

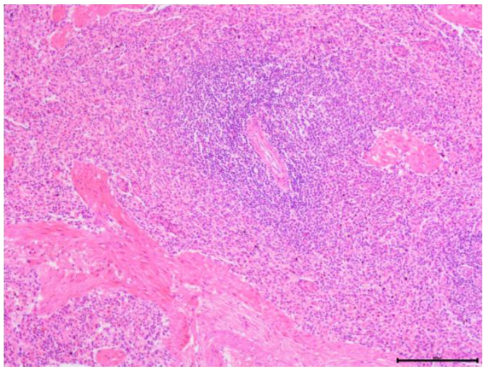

Canine spleen tissue section

(10×， H.E.)

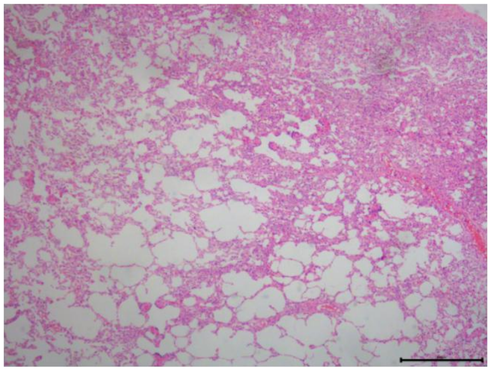

Canine lung tissue section

(4×，H.E.)

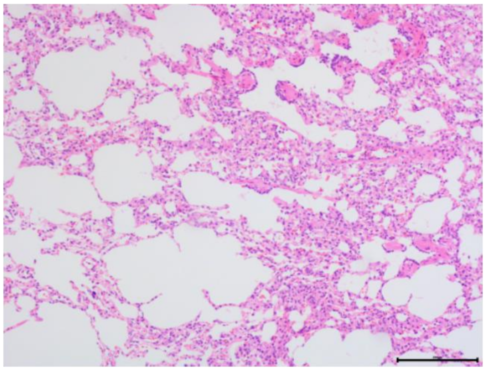

Canine lung tissue section

(10×，H.E.)

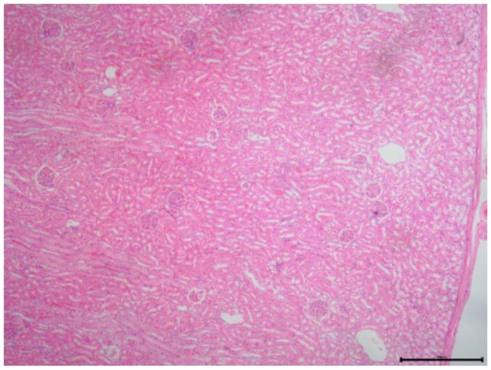

Canine kidney tissue section

(4×，H.E.)

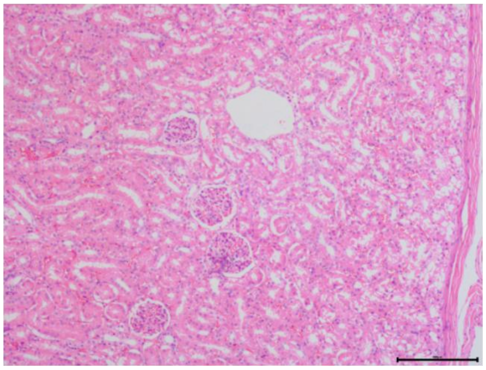

Canine kidney tissue section

(10×，H.E.)

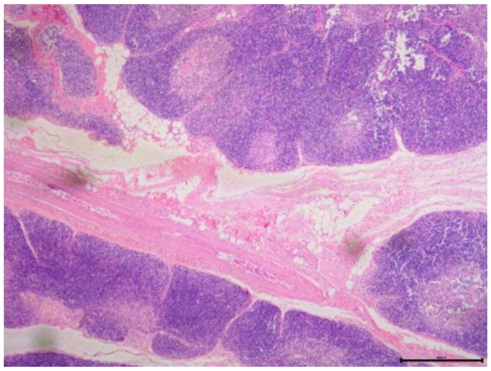

Canine thymus tissue section

(4×，H.E.)

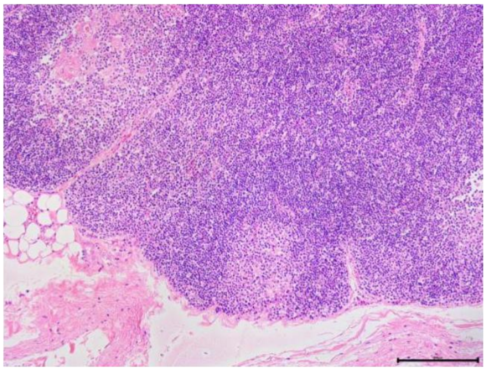

Canine thymus tissue section

(10×，H.E.)

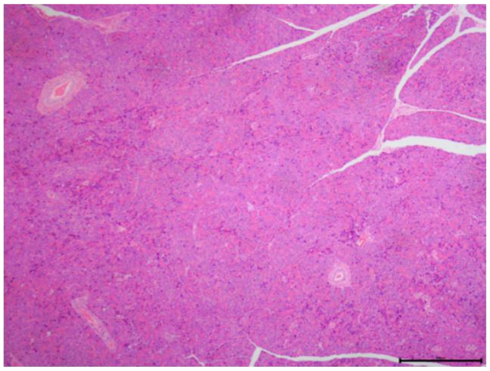

Canine pancreas tissue section

(4×， H.E.)

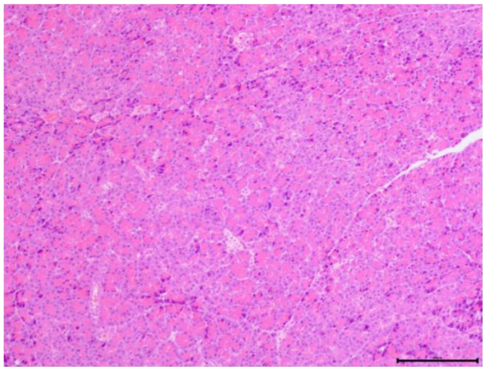

Canine pancreas tissue section

(10×， H.E.)

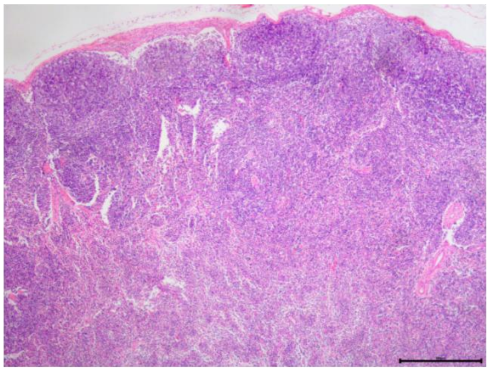

Canine lymph node tissue section

(4×， H.E.)

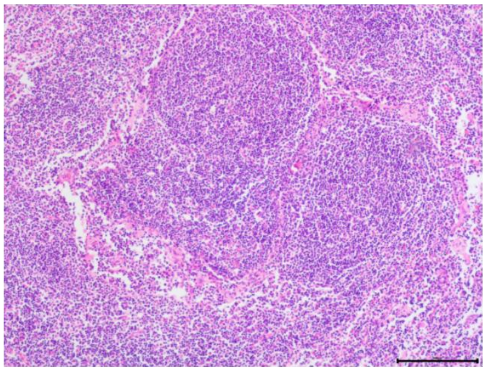

Canine lymph node tissue section

(10×， H.E.)

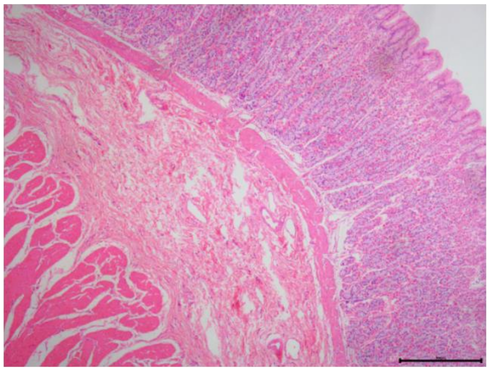

Canine stomach tissue section

(4×， H.E.)

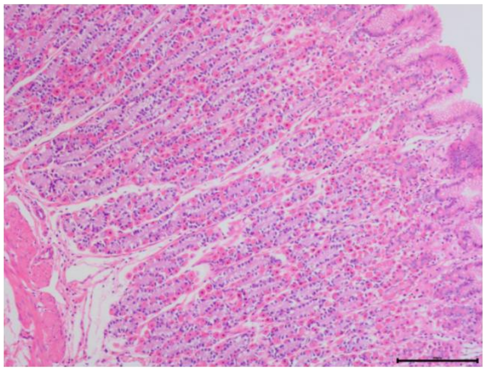

Canine stomach tissue section

(10×， H.E.)

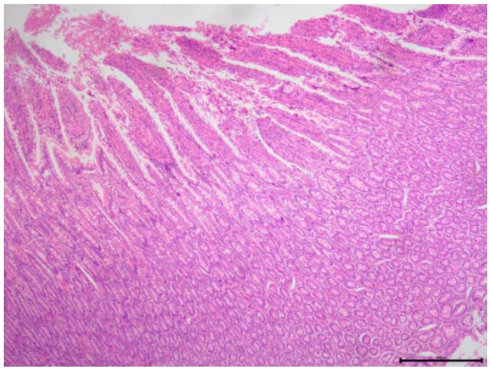

Canine duodenum tissue section

(4×，H.E.)

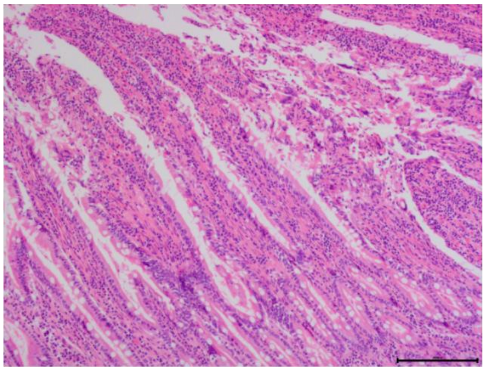

Canine duodenum tissue section

(10×，H.E.)

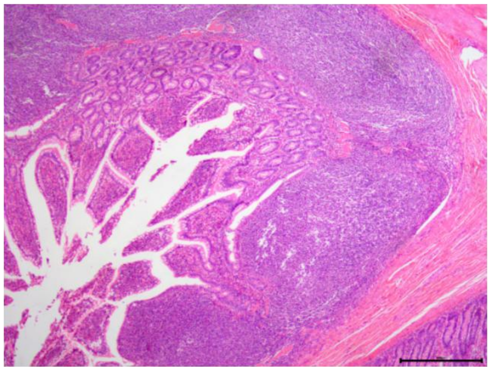

Canine ileum tissue section

(10×，H.E.)

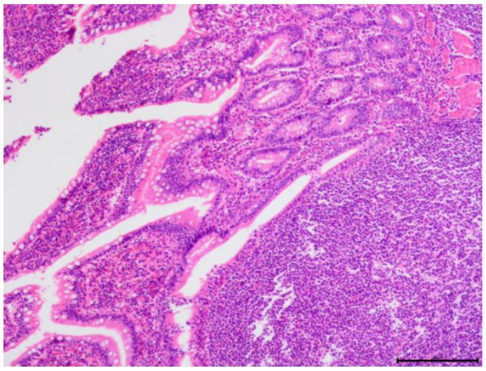

Canine ileum tissue section

(10×，H.E.)

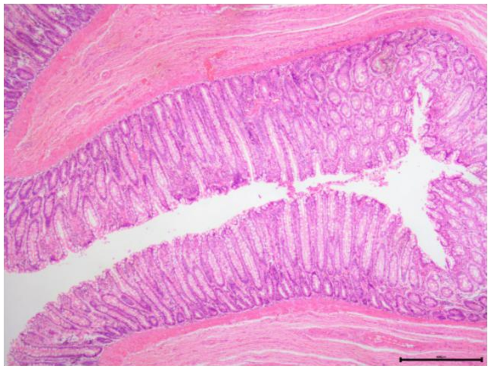

Canine rectum tissue section

(4×，H.E.)

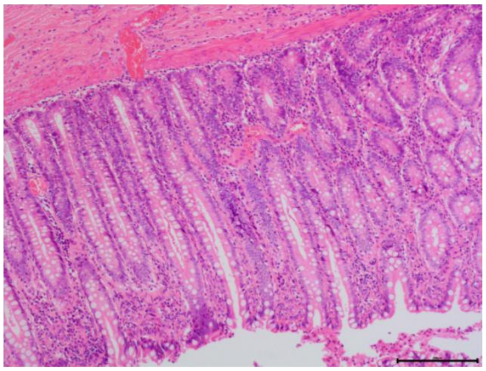

Canine rectum tissue section

(10×，H.E.)

Saline control group——Histopathological sections of Canine No. 12

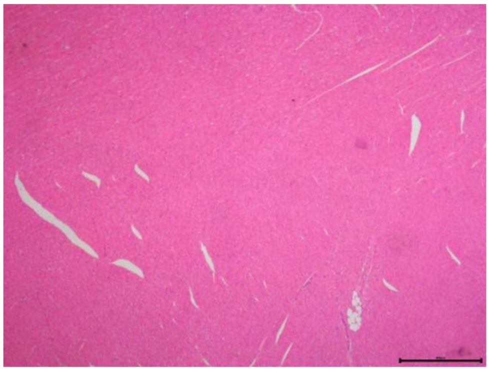

Canine myocardial tissue section(4×，H.E.)

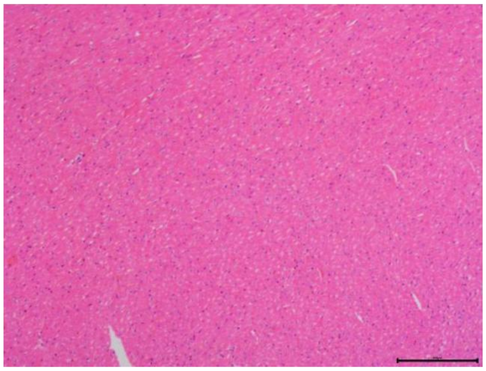

Canine myocardial tissue section(10×，H.E.)

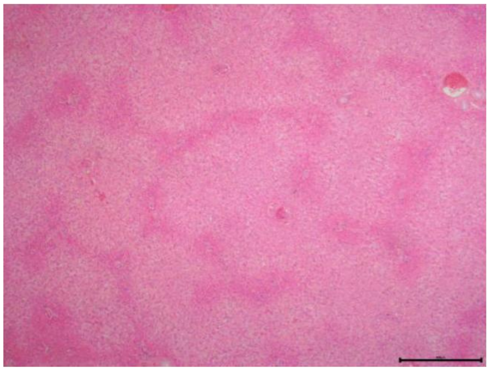

Canine liver tissue section

(4×，H.E.)

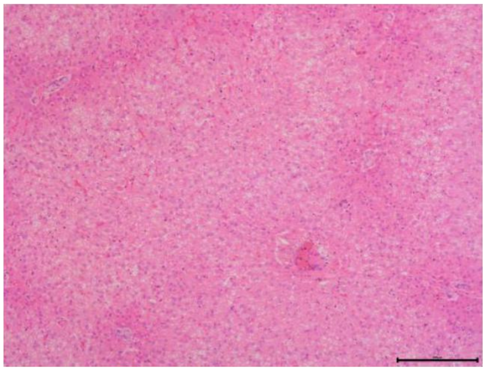

Canine liver tissue section

(10×，H.E.)

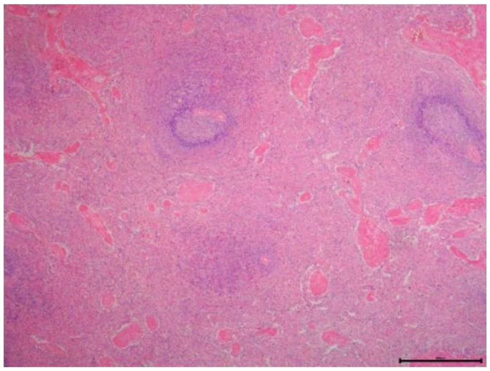

Canine spleen tissue section

(4×，H.E.)

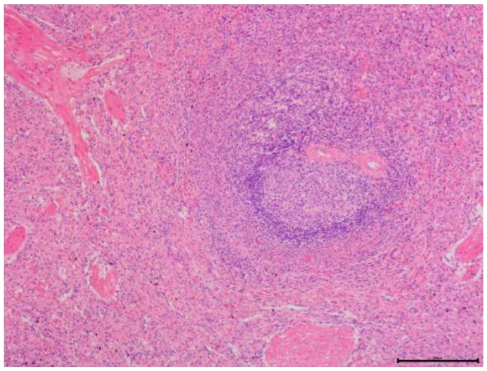

Canine spleen tissue section

(10×，H.E.)

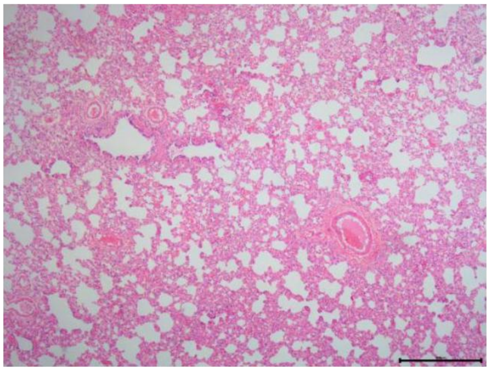

Canine lung tissue section

(4×，H.E.)

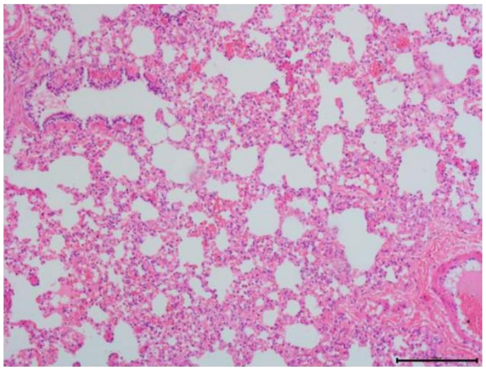

Canine lung tissue section

(10×，H.E.)

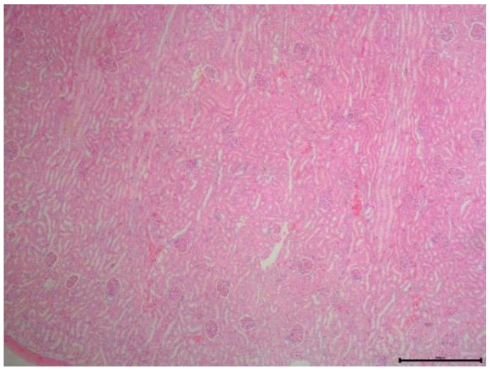

Canine kidney tissue section

(4×，H.E.)

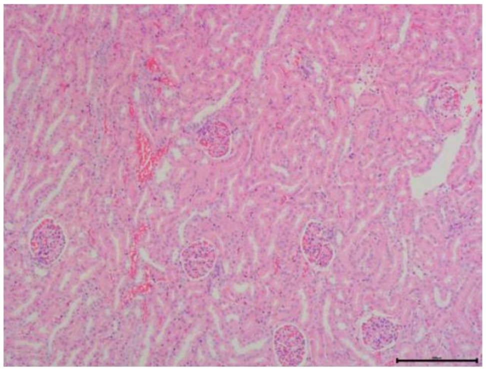

Canine kidney tissue section

(10×，H.E.)

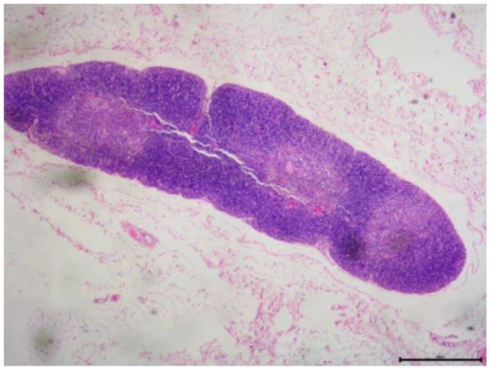

Canine thymus tissue section

(4×，H.E.)

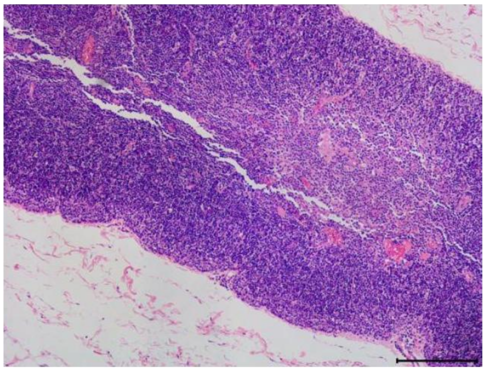

Canine thymus tissue section

(10×，H.E.)

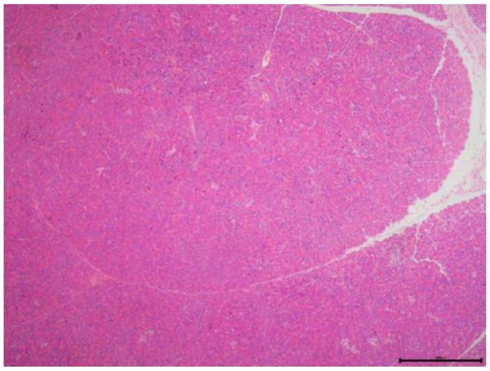

Canine pancreas tissue section

(4×，H.E.)

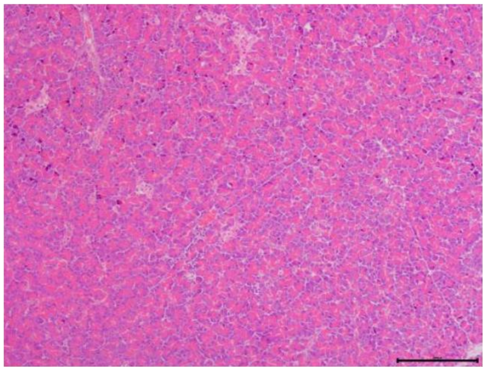

Canine pancreas tissue section

(10×，H.E.)

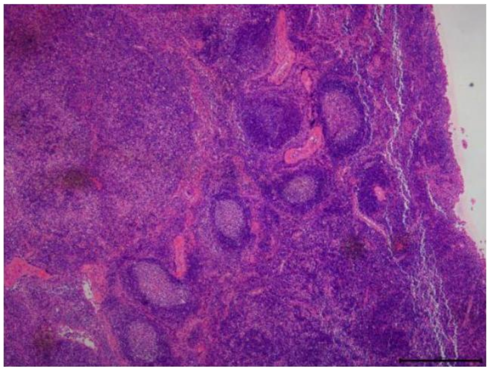

Canine lymph node tissue section

(4×，H.E.)

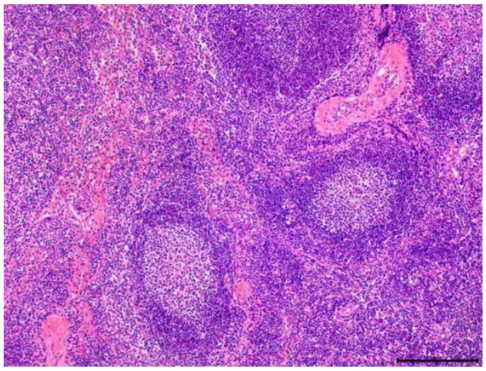

Canine lymph node tissue section

(10×，H.E.)

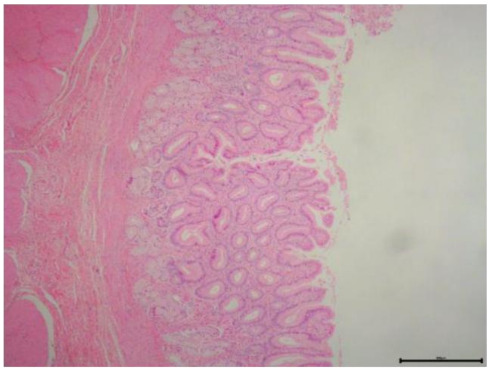

Canine stomach tissue section

(4×，H.E.)

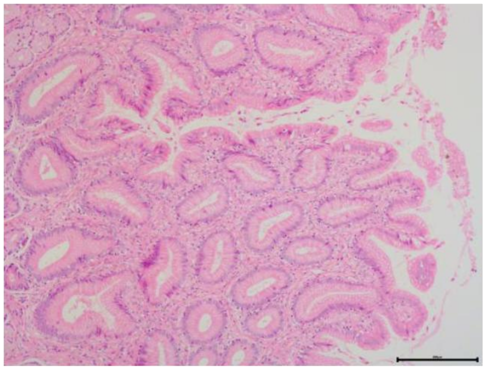

Canine stomach tissue section

(10×，H.E.)

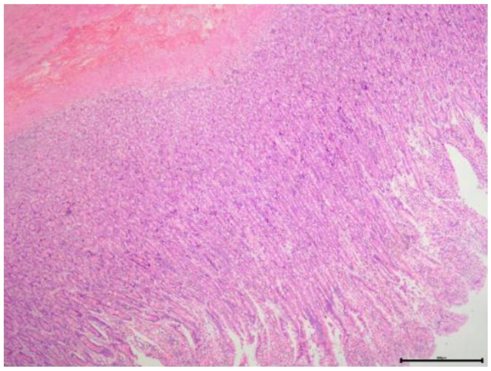

Canine duodenum tissue section

(4×， H.E.)

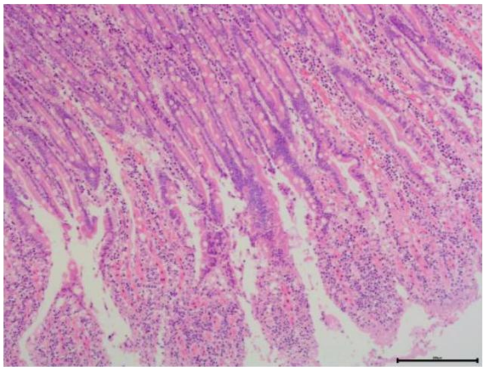

Canine duodenum tissue section

(10×， H.E.)

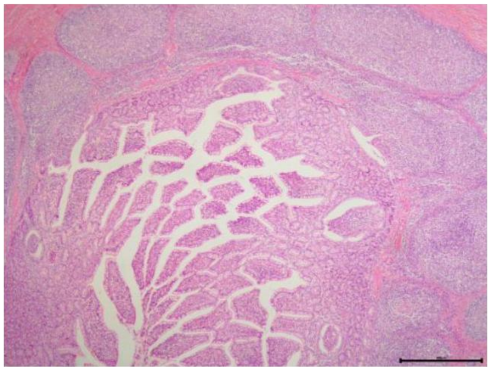

Canine ileum tissue section

(10×， H.E.)

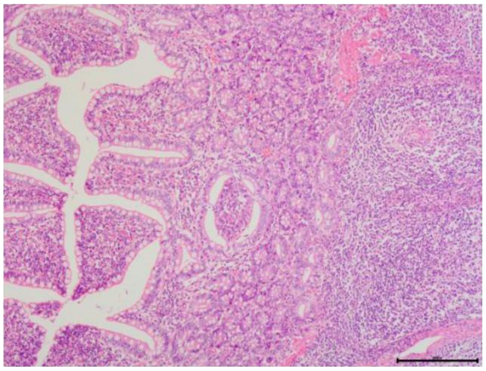

Canine ileum tissue section

(10×， H.E.)

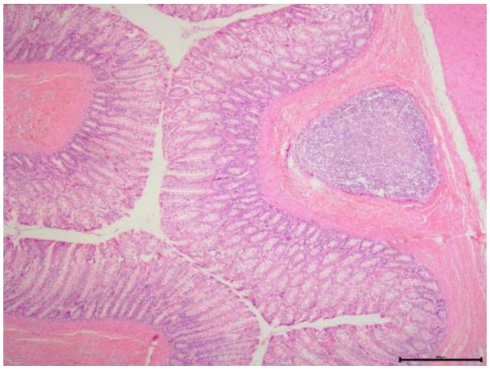

Canine rectum tissue section

(4×， H.E.)

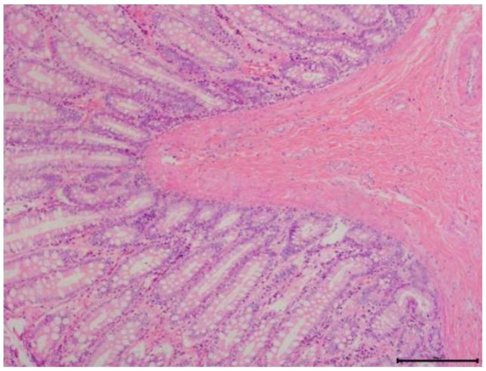

Canine rectum tissue section

(10×， H.E.)

Saline control group——Histopathological sections of Canine No. 19

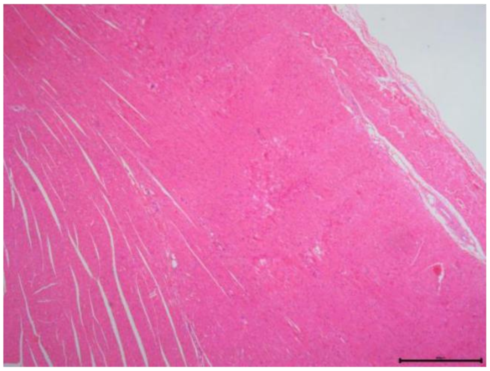

Canine myocardial tissue section(4×，H.E.)

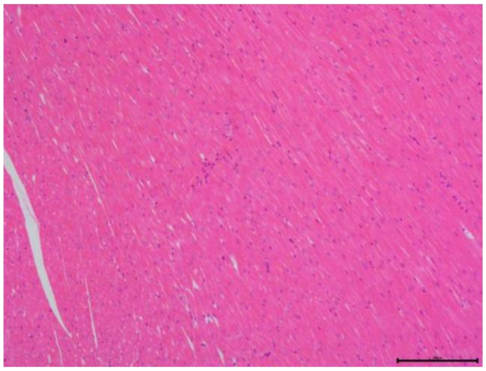

Canine myocardial tissue section(10×，H.E.)

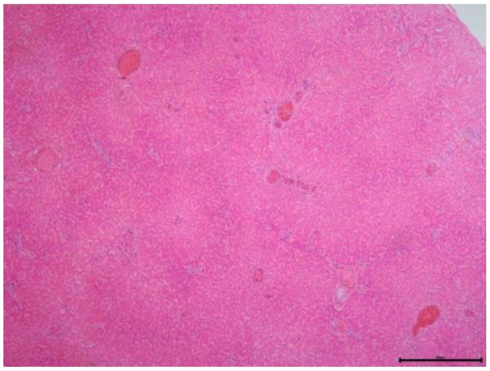

Canine liver tissue section

(4×，H.E.)

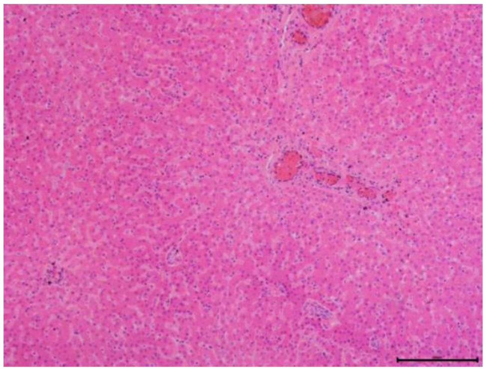

Canine liver tissue section

(10×，H.E.)

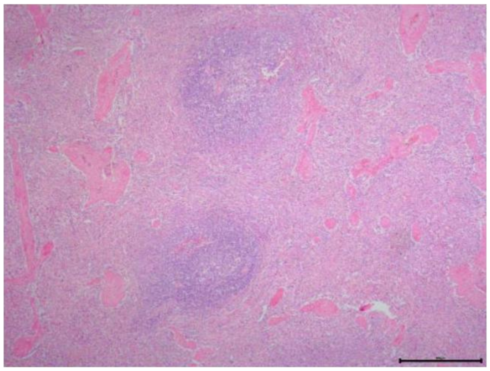

Canine spleen tissue section

(4×，H.E.)

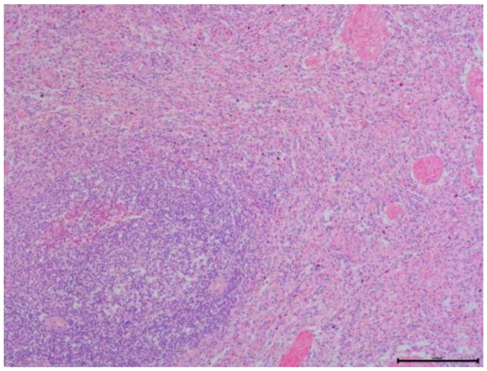

Canine spleen tissue section

(10×，H.E.)

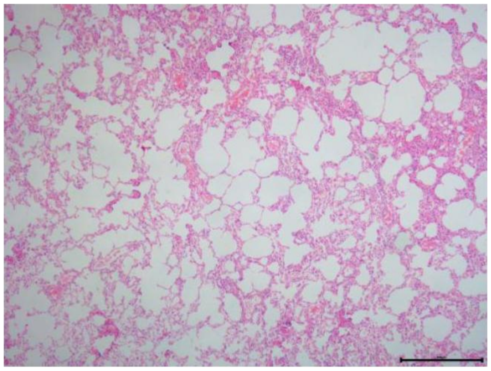

Canine lung tissue section

(4×，H.E.)

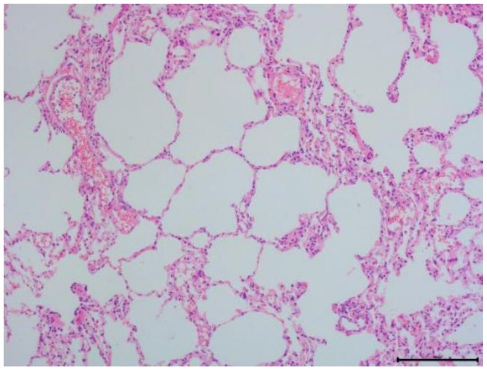

Canine lung tissue section

(10×，H.E.)

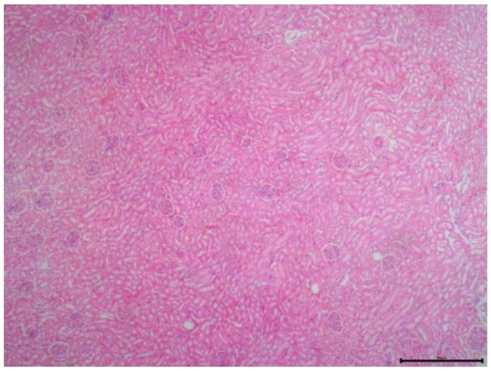

Canine kidney tissue section

(4×，H.E.)

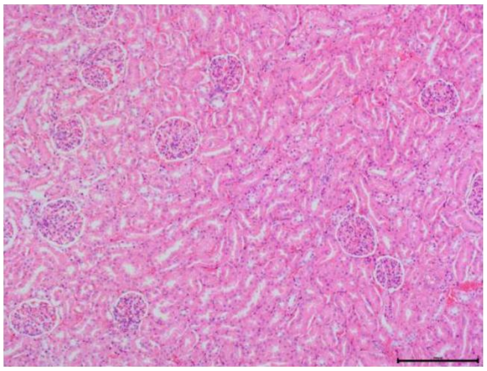

Canine kidney tissue section

(10×，H.E.)

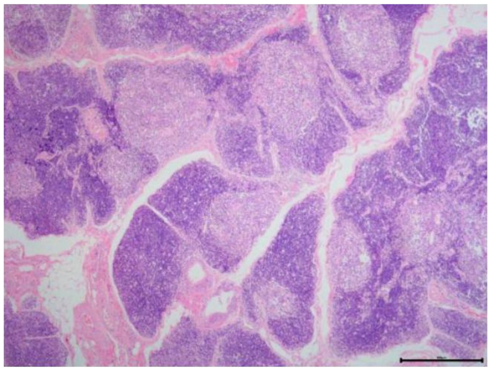

Canine thymus tissue section

(4×，H.E.)

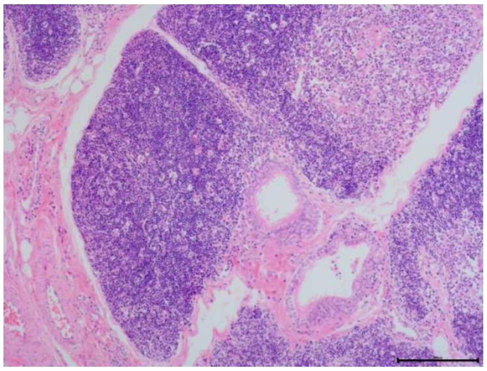

Canine thymus tissue section

(10×，H.E.)

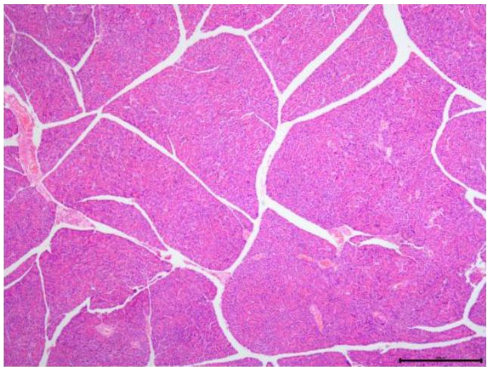

Canine pancreas tissue section

(4×，H.E.)

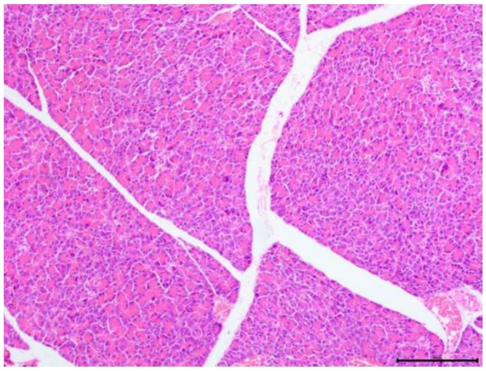

Canine pancreas tissue section

(10×，H.E.)

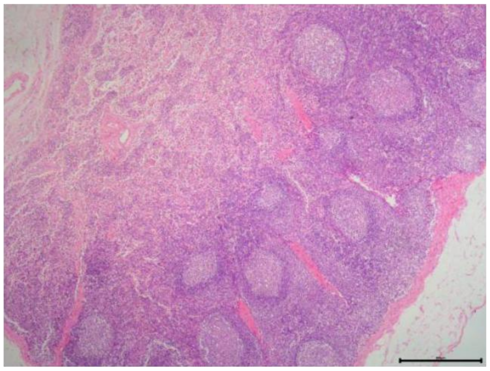

Canine lymph node tissue section

(4×，H.E.)

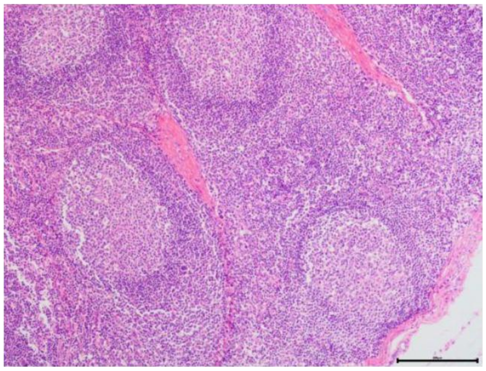

Canine lymph node tissue section

(10×，H.E.)

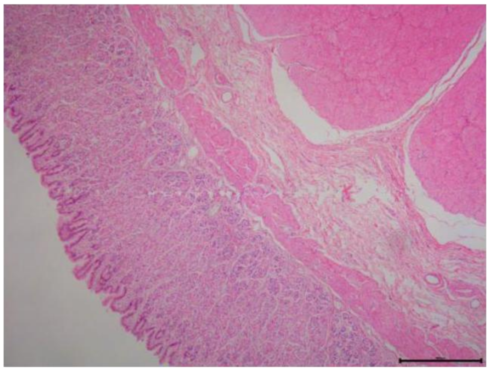

Canine stomach tissue section

(4×，H.E.)

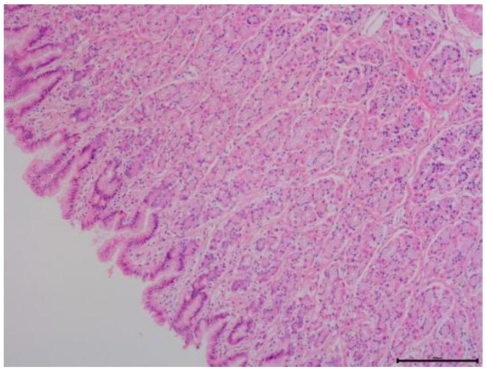

Canine stomach tissue section

(10×，H.E.)

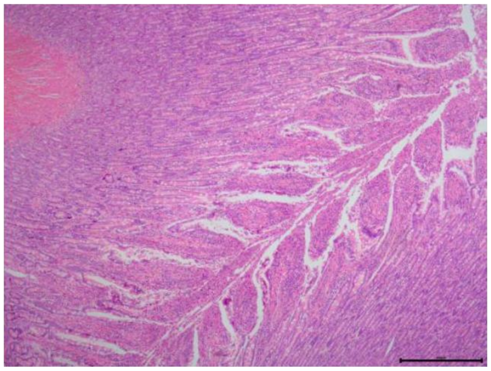

Canine duodenum tissue section

(4×，H.E.)

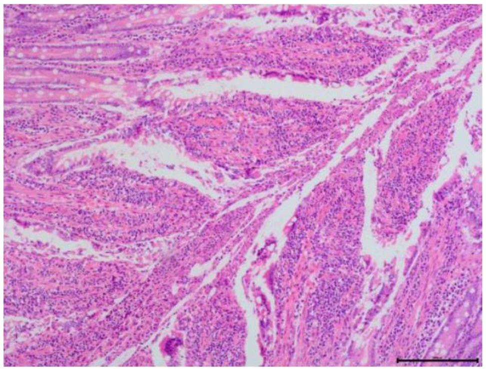

Canine duodenum tissue section

(10×，H.E.)

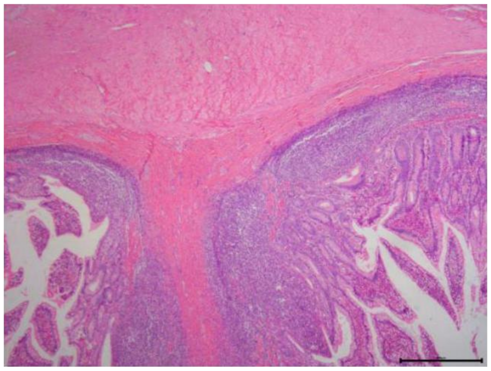

Canine ileum tissue section

(10×，H.E.)

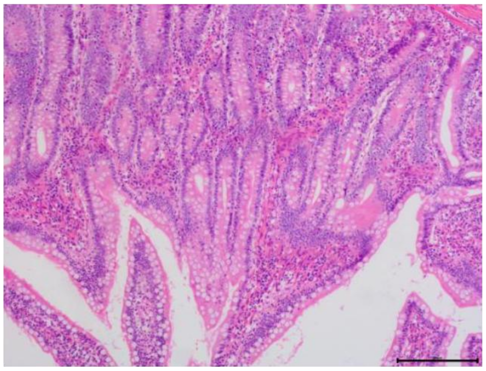

Canine ileum tissue section

(10×，H.E.)

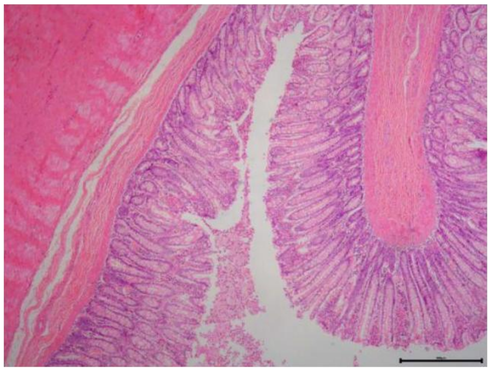

Canine rectum tissue section

(4×，H.E.)

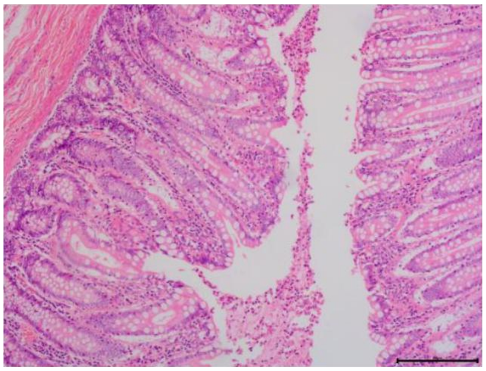

Canine rectum tissue section

(10×，H.E.)

Saline control group——Histopathological sections of Canine No. 20

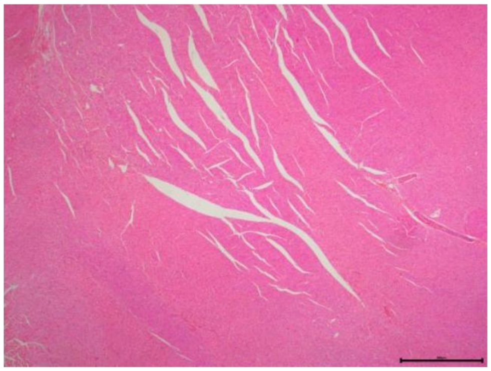

Canine myocardial tissue section(4×，H.E.)

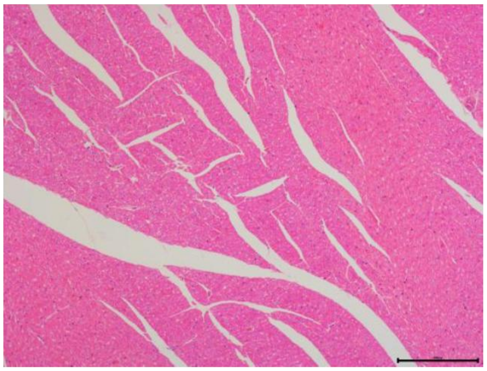

Canine myocardial tissue section(10×，H.E.)

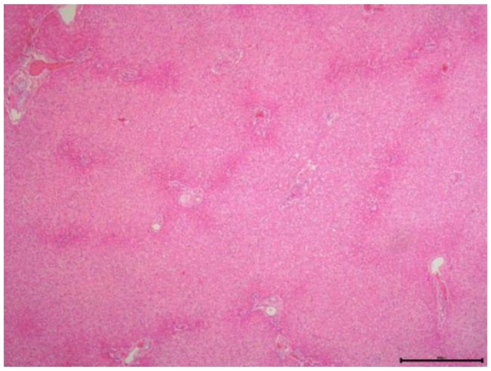

Canine liver tissue section

(4×，H.E.)

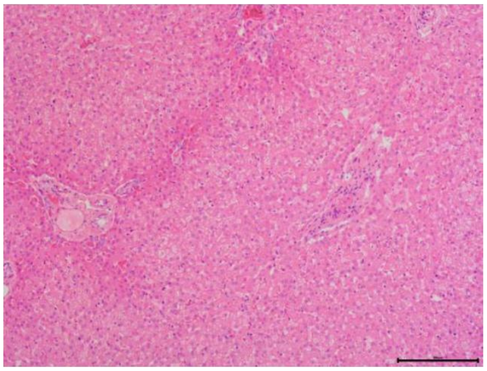

Canine liver tissue section

(10×，H.E.)

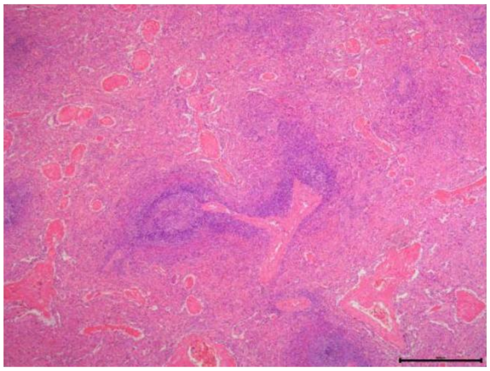

Canine spleen tissue section

(4×，H.E.)

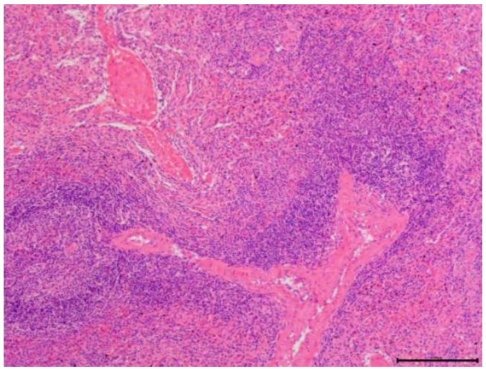

Canine spleen tissue section

(10×，H.E.)

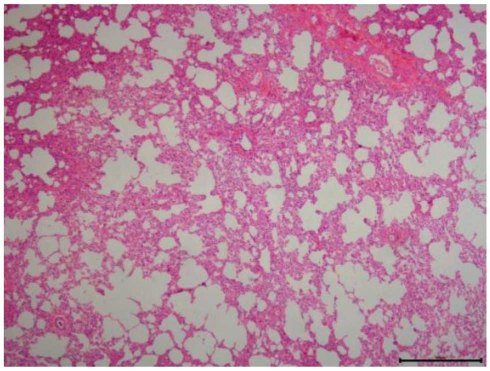

Canine lung tissue section

(4×，H.E.)

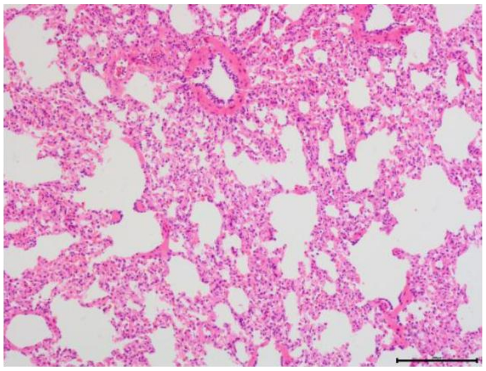

Canine lung tissue section

(10×，H.E.)

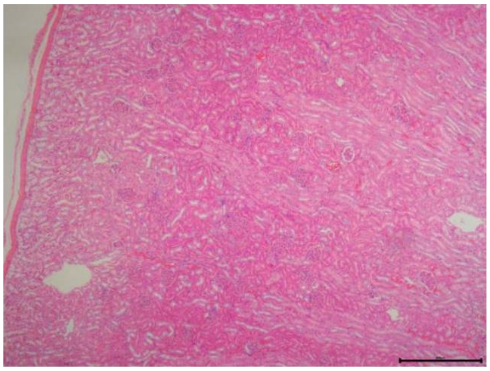

Canine kidney tissue section

(4×，H.E.)

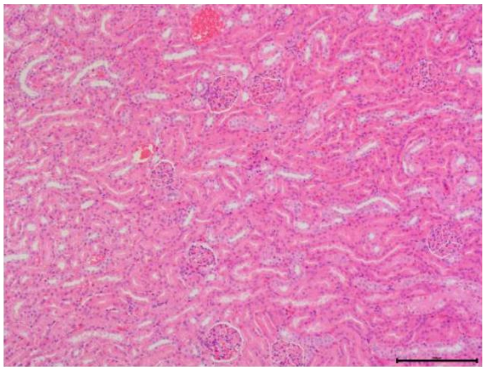

Canine kidney tissue section

(10×，H.E.)

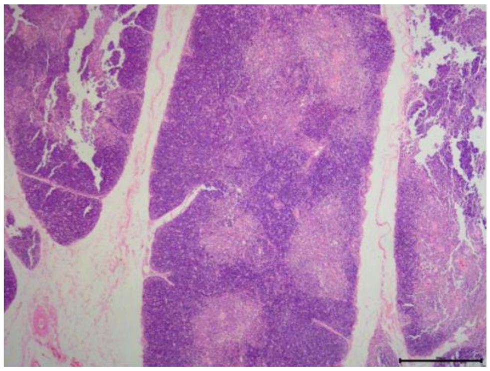

Canine thymus tissue section

(4×，H.E.)

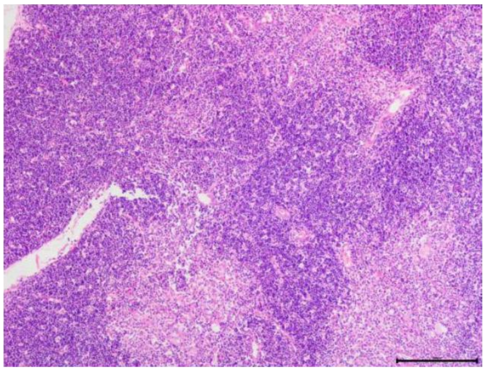

Canine thymus tissue section

(10×，H.E.)

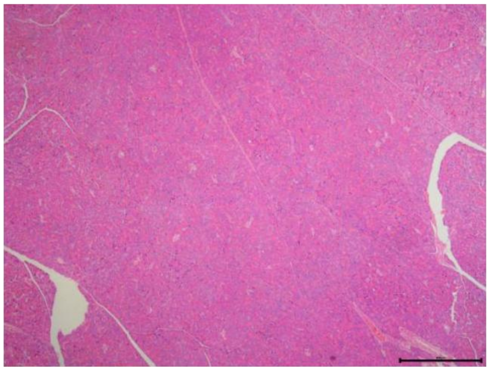

Canine pancreas tissue section

(4×，H.E.)

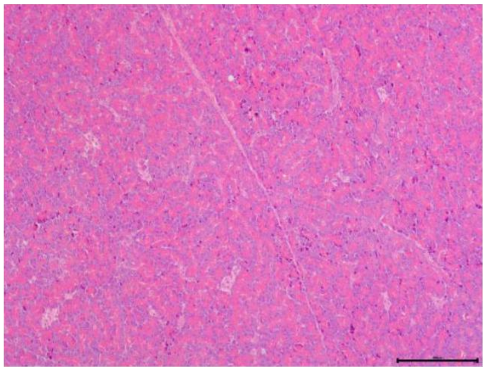

Canine pancreas tissue section

(10×，H.E.)

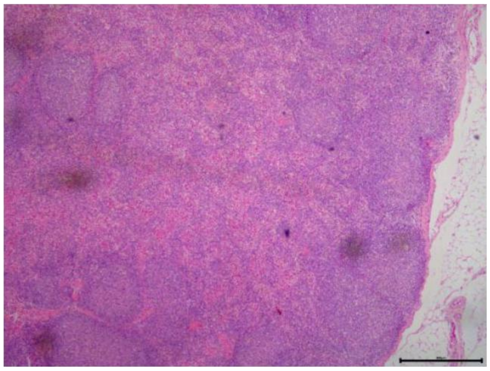

Canine lymph node tissue section

(4×，H.E.)

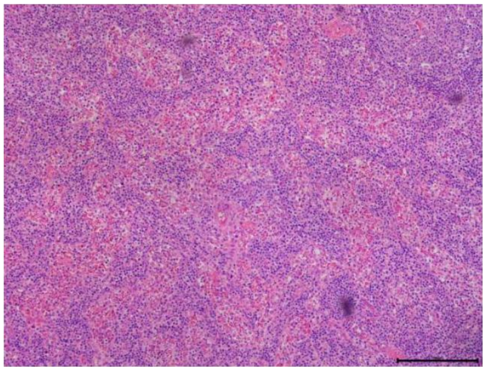

Canine lymph node tissue section

(10×，H.E.)

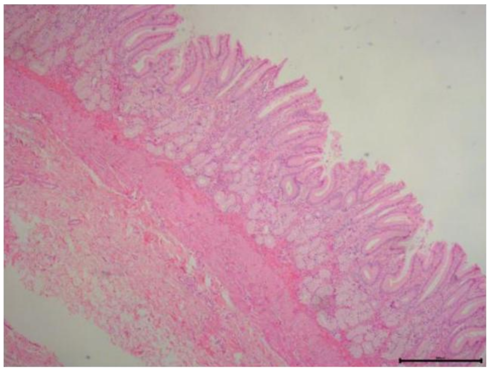

Canine stomach tissue section

(4×，H.E.)

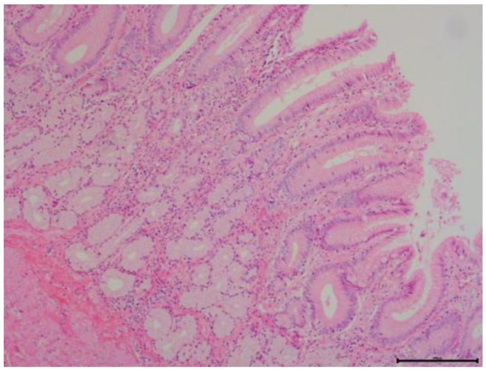

Canine stomach tissue section

(10×，H.E.)

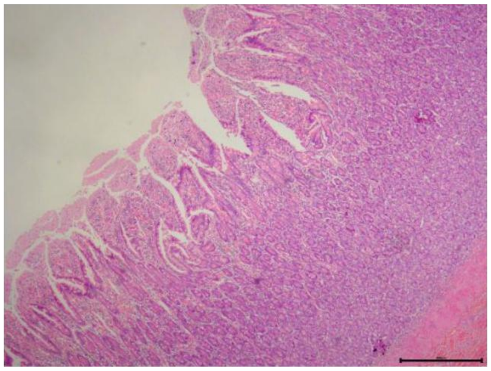

Canine duodenum tissue section

(4×， H.E.)

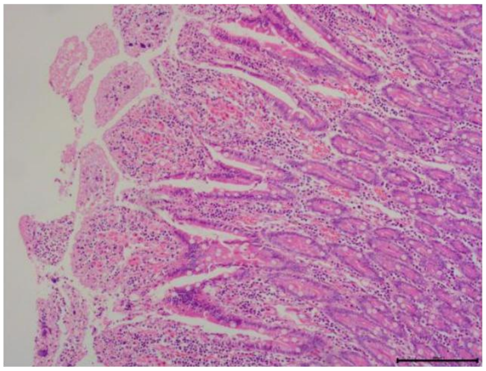

Canine duodenum tissue section

(10×， H.E.)

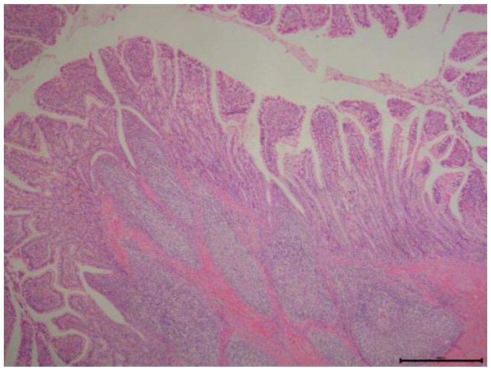

Canine ileum tissue section

(10×， H.E.)

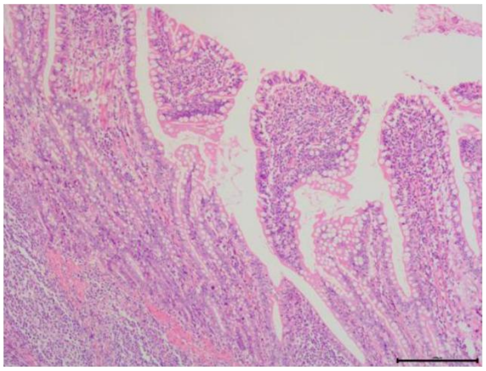

Canine ileum tissue section

(10×， H.E.)

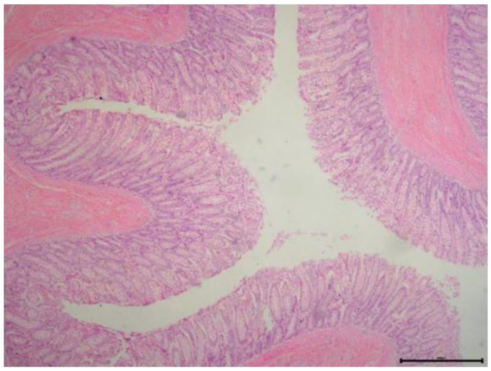

Canine rectum tissue section

(4×， H.E.)

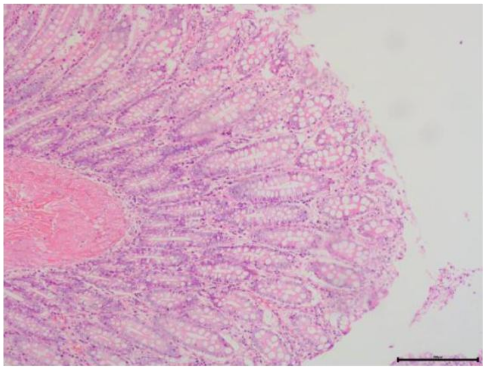

Canine rectum tissue section

(10×， H.E.)

Saline control group——Histopathological sections of Canine No. 23

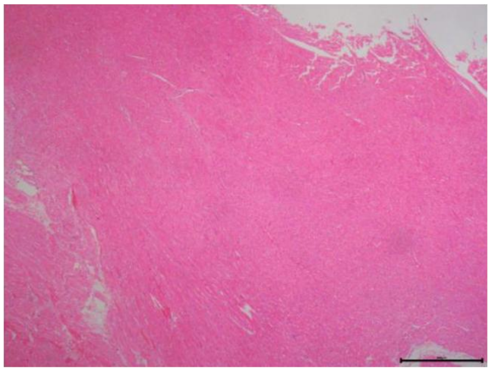

Canine myocardial tissue section(4×， H.E.)

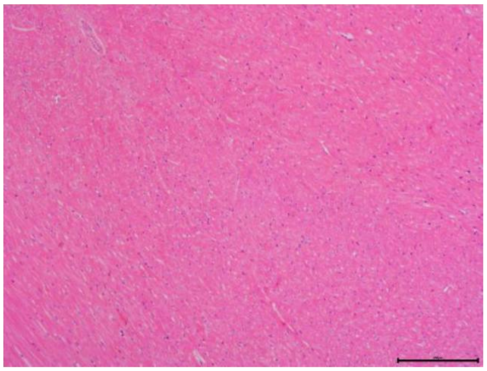

Canine myocardial tissue section(10×， H.E.)

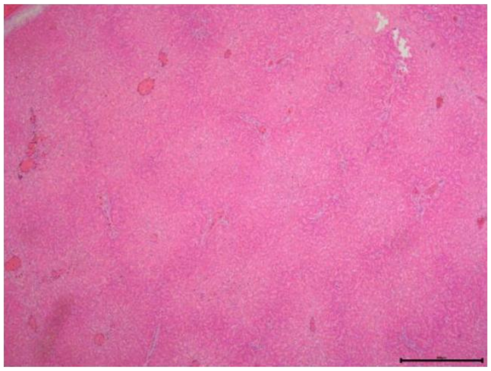

Canine liver tissue section

(4×， H.E.)

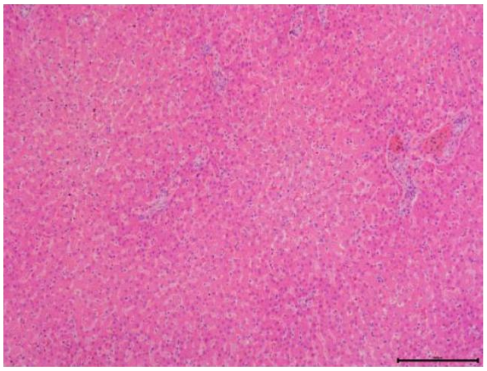

Canine liver tissue section

(10×， H.E.)

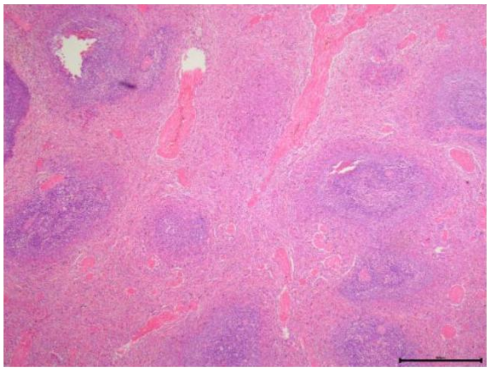

Canine spleen tissue section

(4×， H.E.)

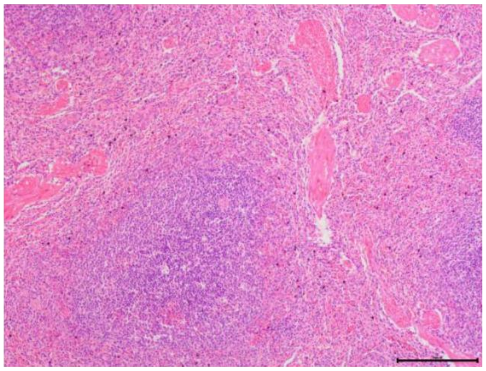

Canine spleen tissue section

(10×， H.E.)

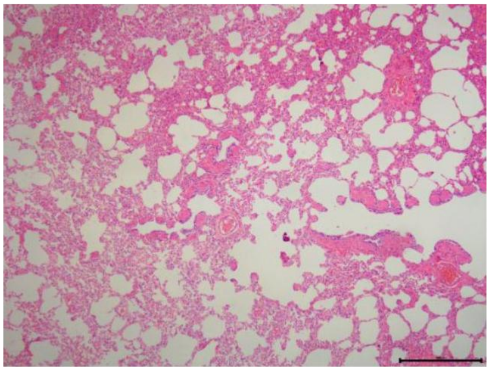

Canine lung tissue section

(4×，H.E.)

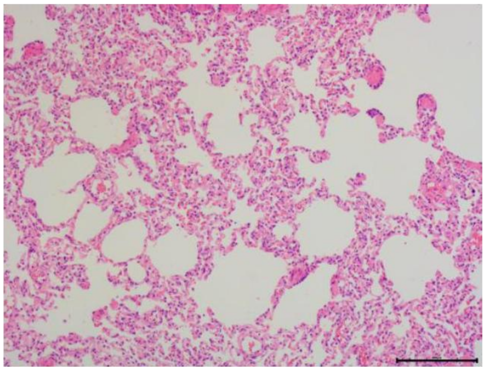

Canine lung tissue section

(10×，H.E.)

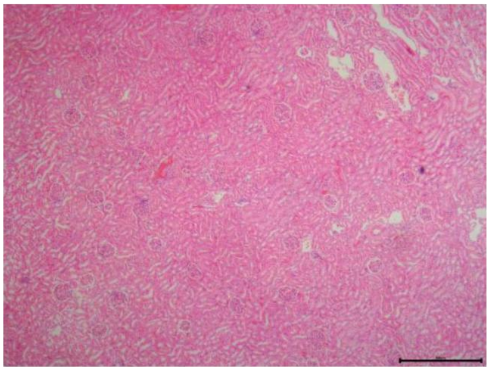

Canine kidney tissue section

(4×，H.E.)

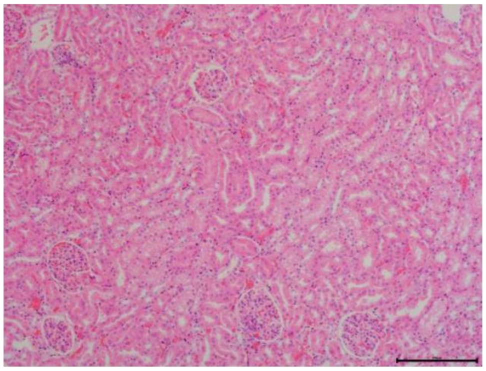

Canine kidney tissue section

(10×，H.E.)

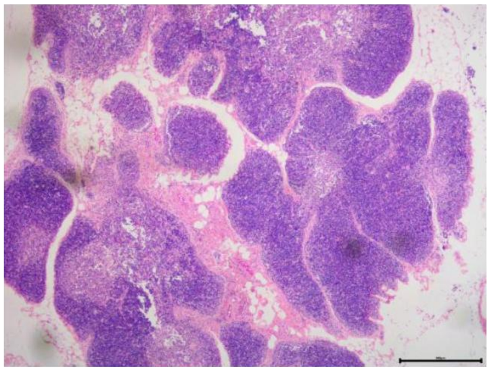

Canine thymus tissue section

(4×，H.E.)

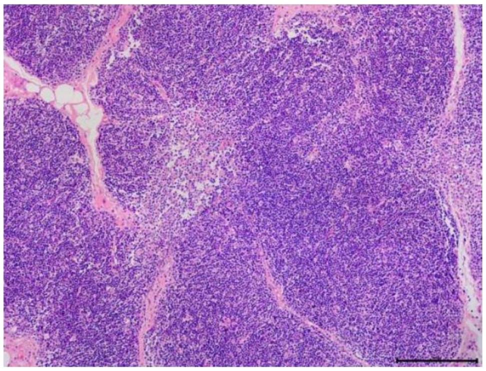

Canine thymus tissue section

(10×，H.E.)

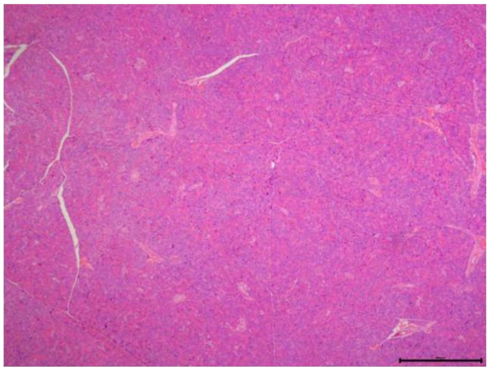

Canine pancreas tissue section

(4×，H.E.)

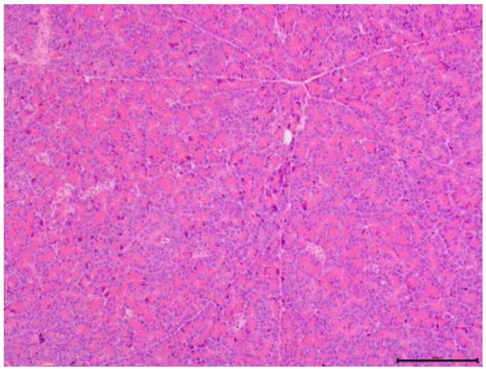

Canine pancreas tissue section

(10×，H.E.)

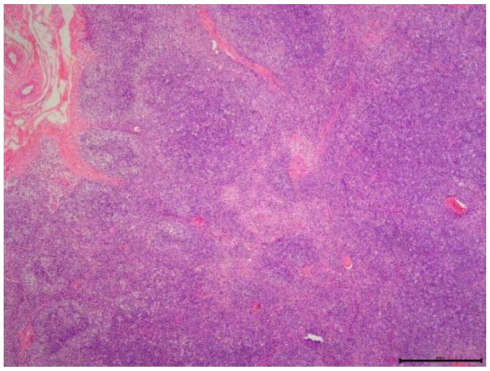

Canine lymph node tissue section

(4×，H.E.)

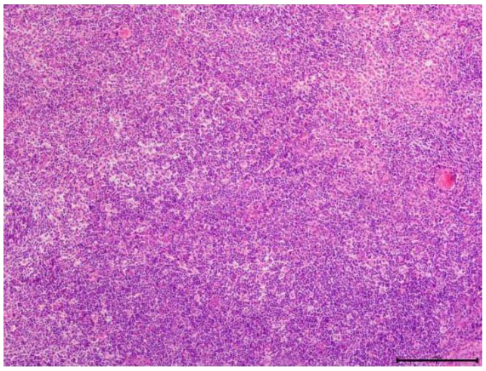

Canine lymph node tissue section

(10×，H.E.)

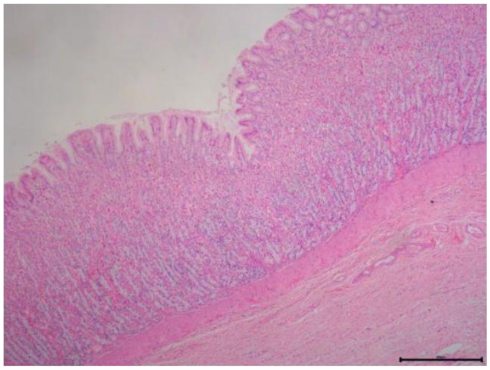

Canine stomach tissue section

(4×，H.E.)

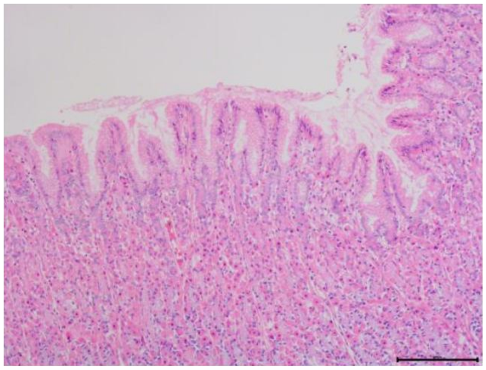

Canine stomach tissue section

(10×，H.E.)

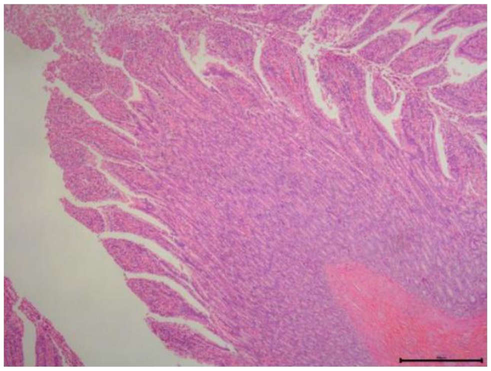

Canine duodenum tissue section

(4×， H.E.)

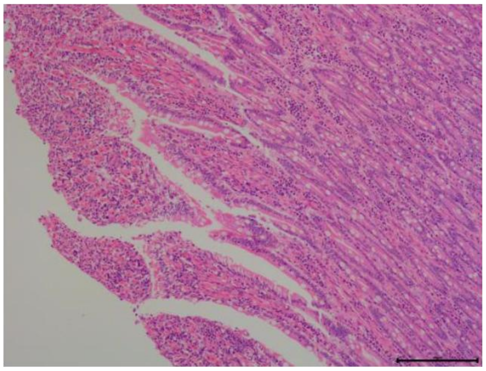

Canine duodenum tissue section

(10×， H.E.)

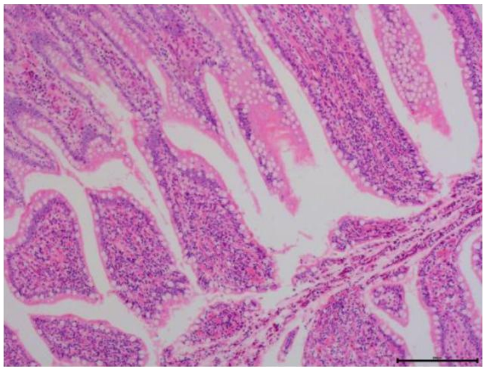

Canine ileum tissue section

(10×， H.E.)

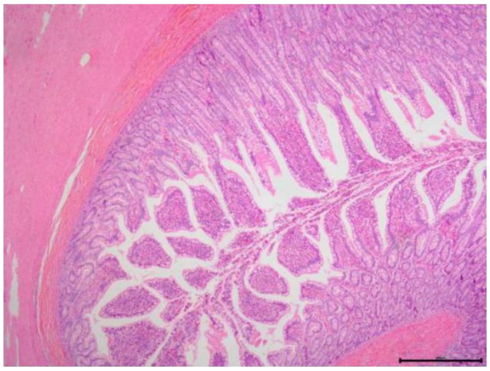

Canine ileum tissue section

(10×， H.E.)

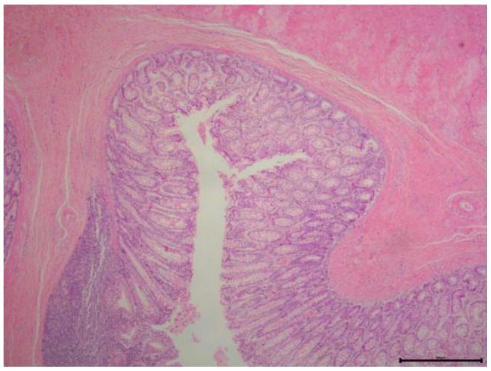

Canine rectum tissue section

(4×， H.E.)

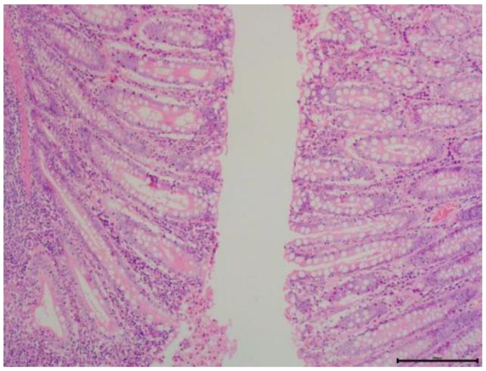

Canine rectum tissue section

(10×， H.E.)

Saline control group——Histopathological sections of Canine No. 27

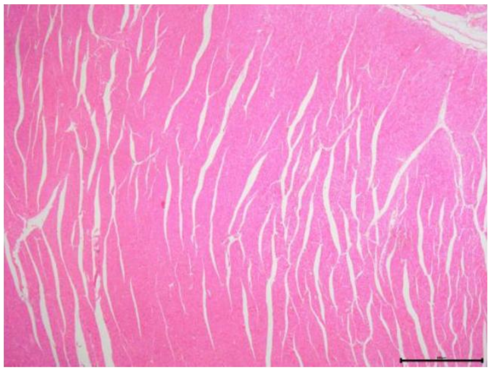

Canine myocardial tissue section(4×，H.E.)

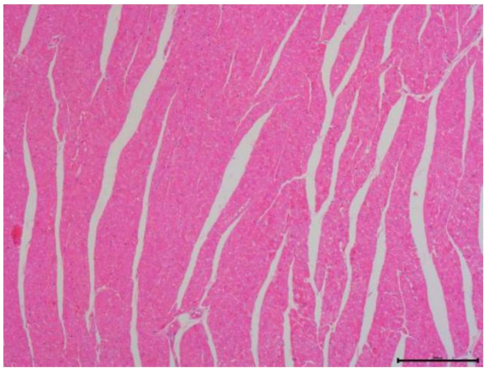

Canine myocardial tissue section(10×，H.E.)

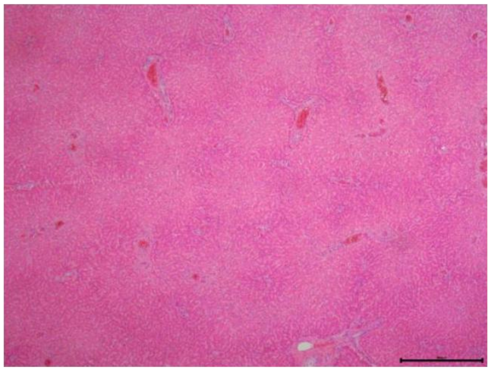

Canine liver tissue section

(4×，H.E.)

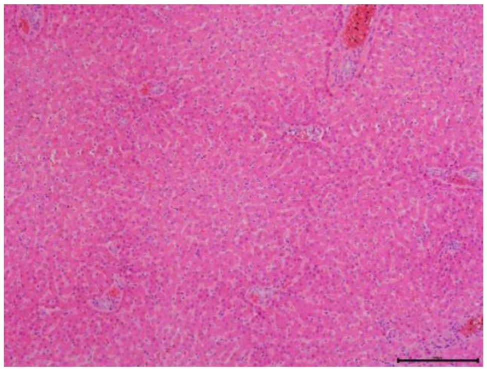

Canine liver tissue section

(10×，H.E.)

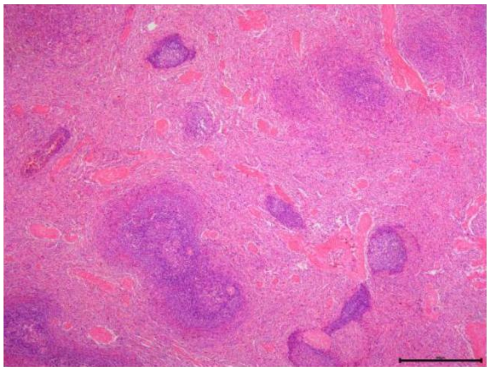

Canine spleen tissue section

(4×，H.E.)

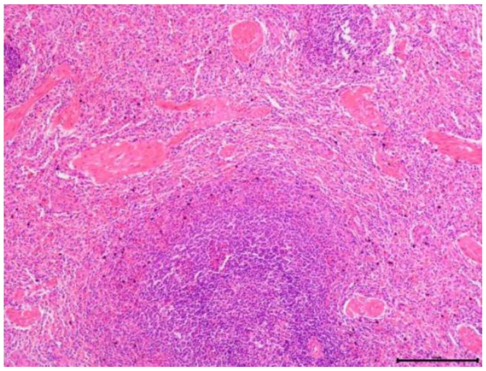

Canine spleen tissue section

(10×，H.E.)

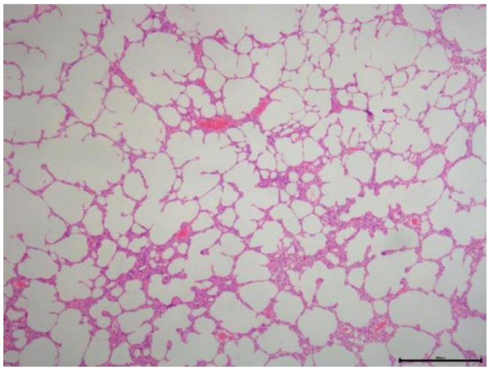

Canine lung tissue section

(4×，H.E.)

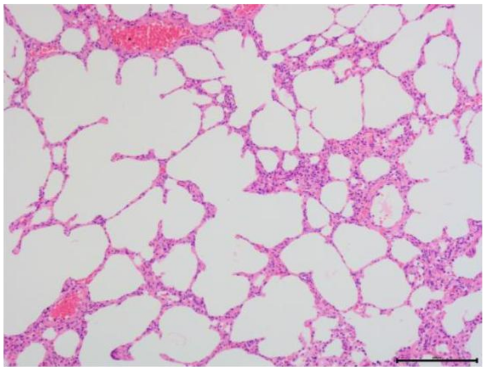

Canine lung tissue section

(10×，H.E.)

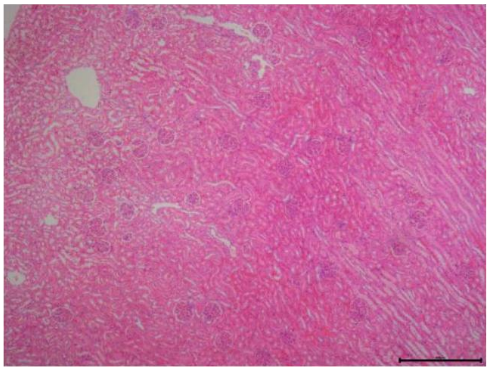

Canine kidney tissue section

(4×，H.E.)

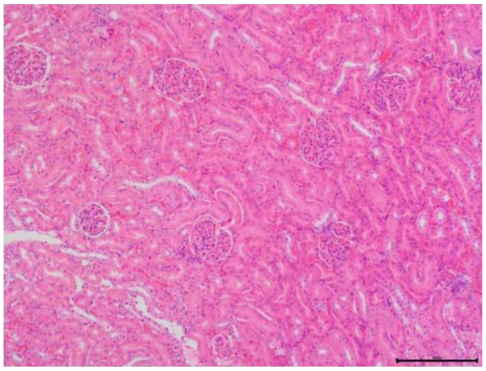

Canine kidney tissue section

(10×，H.E.)

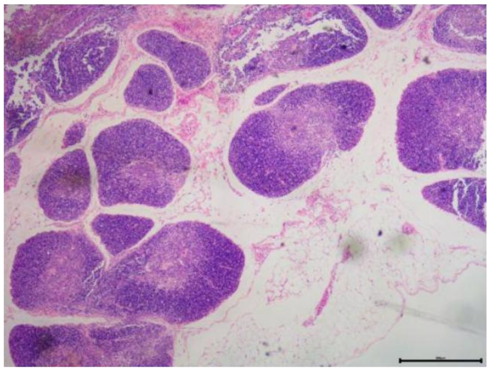

Canine thymus tissue section

(4×，H.E.)

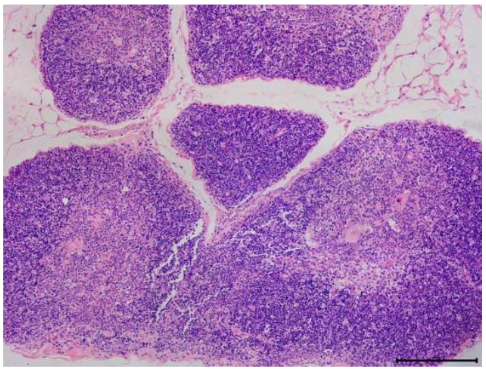

Canine thymus tissue section

(10×，H.E.)

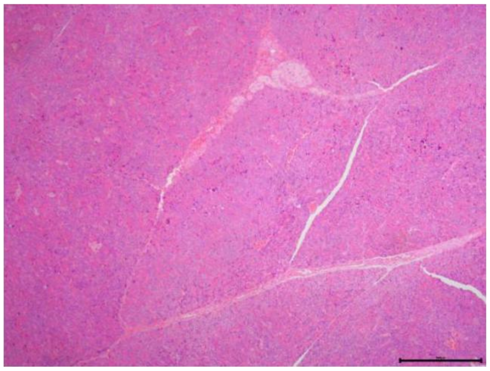

Canine pancreas tissue section

(4×，H.E.)

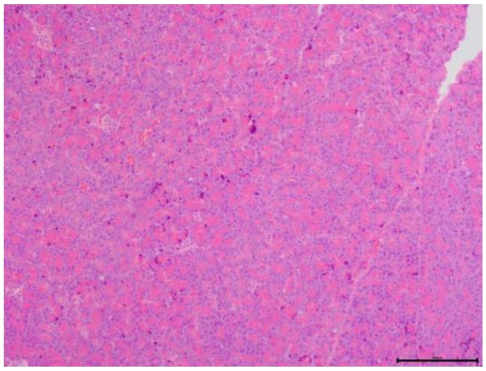

Canine pancreas tissue section

(10×，H.E.)

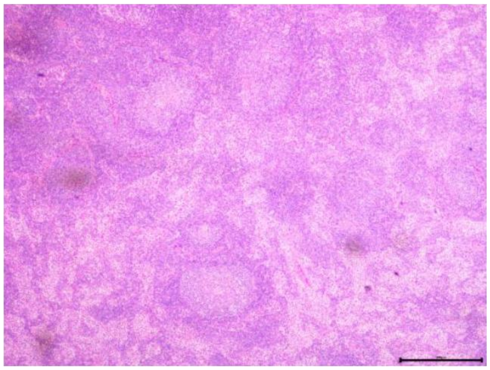

Canine lymph node tissue section

(4×，H.E.)

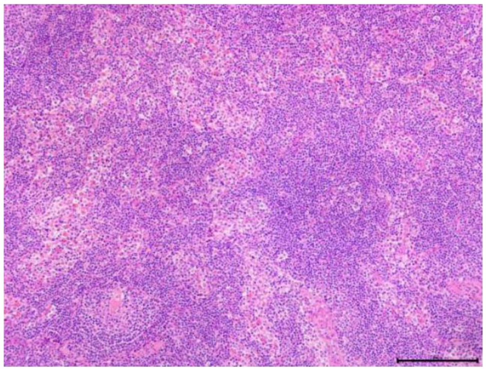

Canine lymph node tissue section

(10×，H.E.)

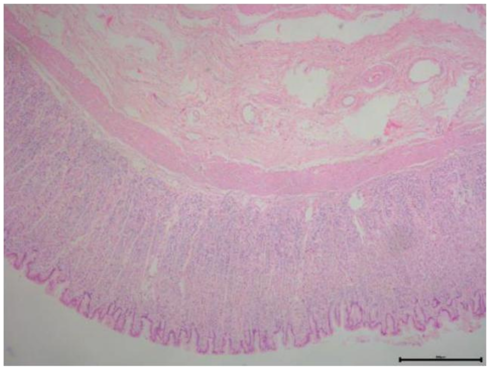

Canine stomach tissue section

(4×，H.E.)

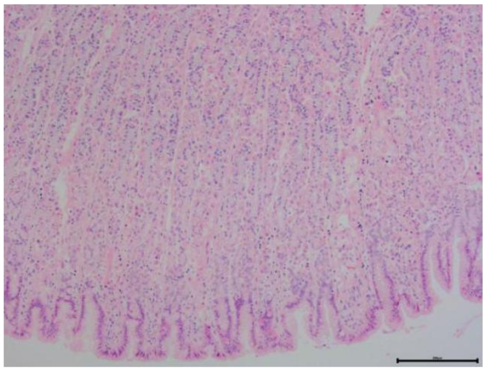

Canine stomach tissue section

(10×，H.E.)

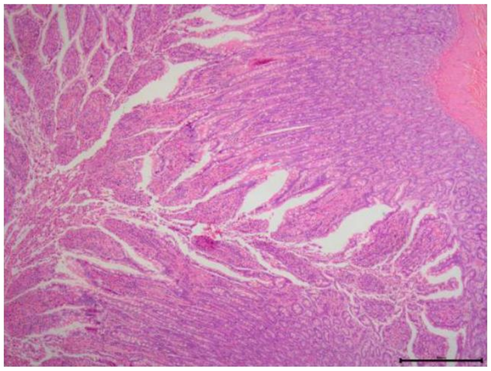

Canine duodenum tissue section

(4×，H.E.)

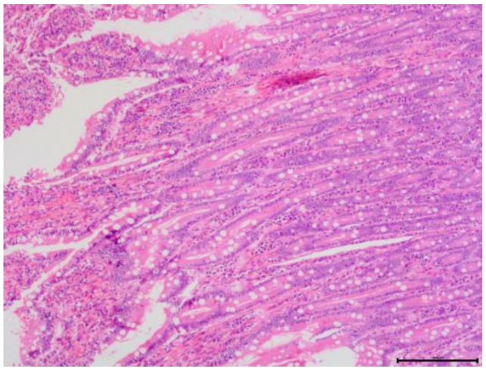

Canine duodenum tissue section

(10×，H.E.)

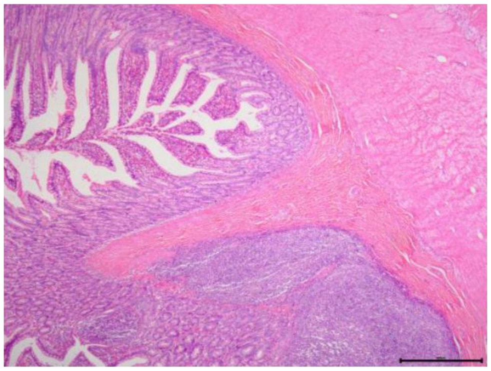

Canine ileum tissue section

(10×，H.E.)

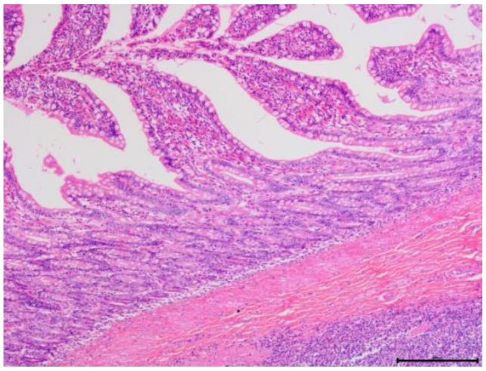

Canine ileum tissue section

(10×，H.E.)

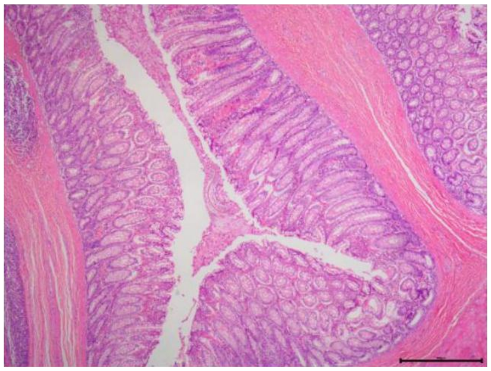

Canine rectum tissue section

(4×，H.E.)

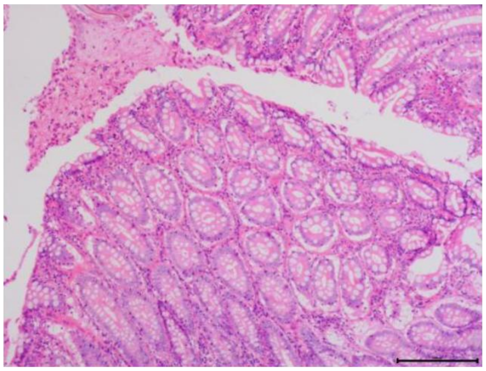

Canine rectum tissue section

(10×，H.E.)

Saline control group——Histopathological sections of Canine No. 30

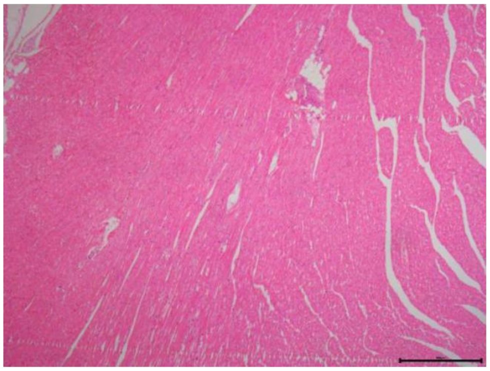

Canine myocardial tissue section(4×，H.E.)

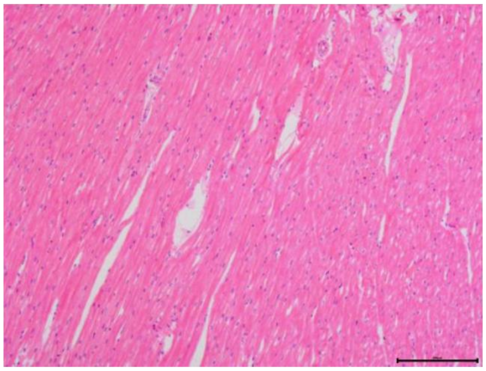

Canine myocardial tissue section(10×，H.E.)

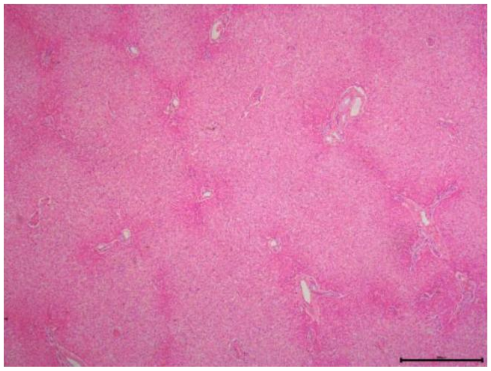

Canine liver tissue section

(4×，H.E.)

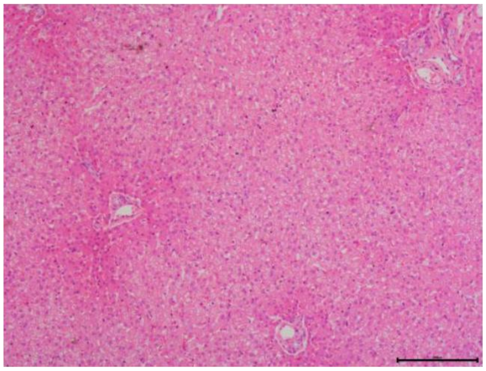

Canine liver tissue section

(10×，H.E.)

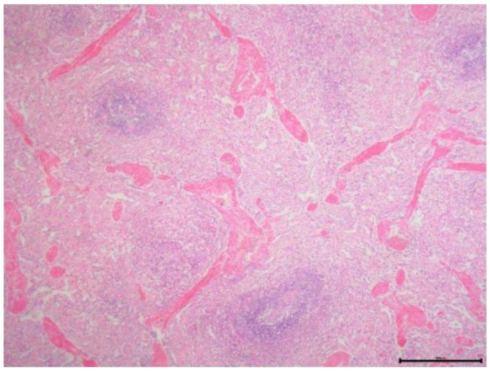

Canine spleen tissue section

(4×，H.E.)

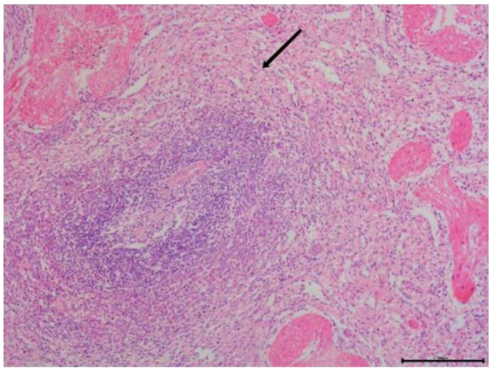

Canine spleen tissue section*1

(10×，H.E.)

"*1": Decreased lymphocytes in the splenic red pulp

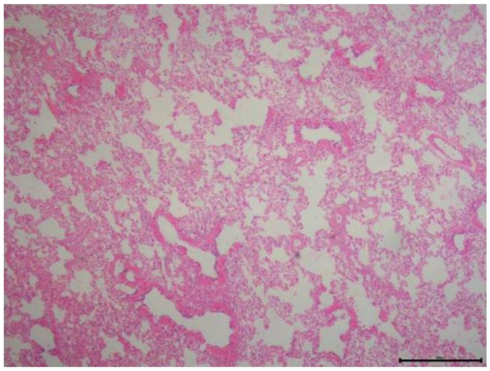

Canine lung tissue section

(4×， H.E.)

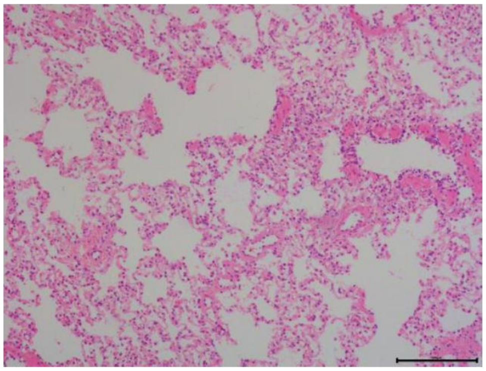

Canine lung tissue section

(10×， H.E.)

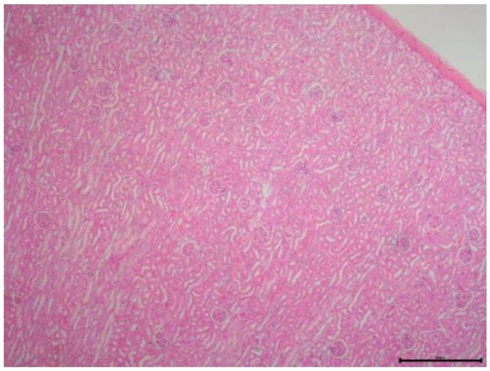

Canine kidney tissue section

(4×， H.E.)

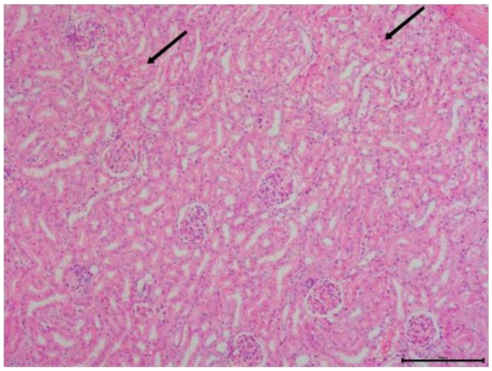

Canine kidney tissue section*2

(10×， H.E.)

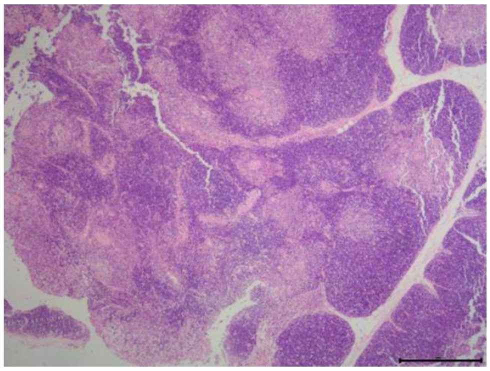

Canine thymus tissue section

(4×， H.E.)

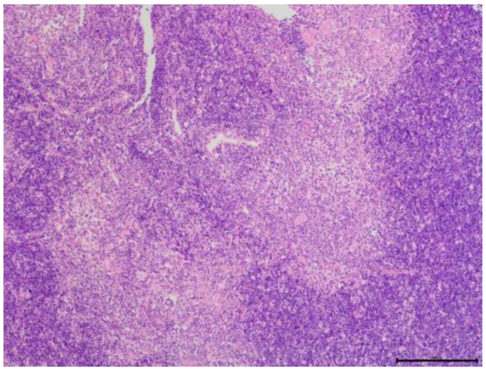

Canine thymus tissue section

(10×， H.E.)

"*2": proteinaceous exudate in renal tubules accompanied by epithelial necrosis.

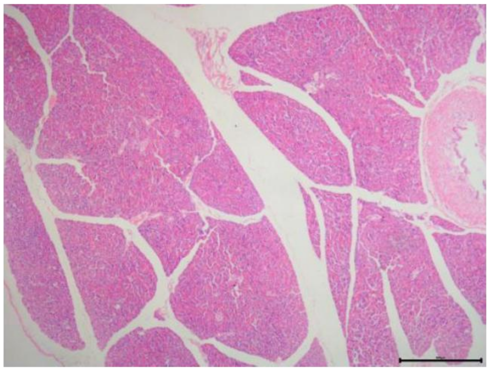

Canine pancreas tissue section

(4×，H.E.)

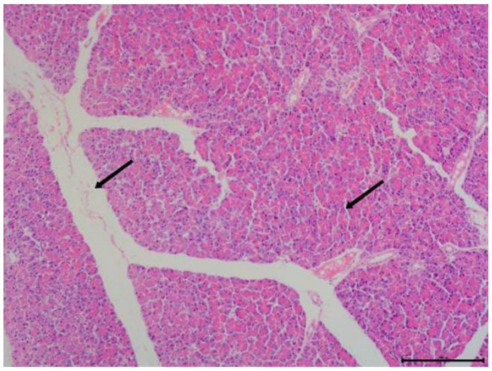

Canine pancreas tissue section*3

(10×，H.E.)

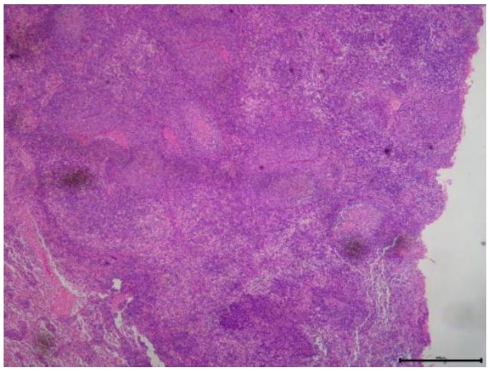

Canine lymph node tissue section

(4×，H.E.)

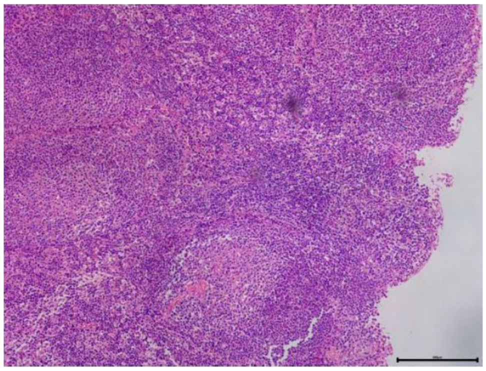

Canine lymph node tissue section

(10×，H.E.)

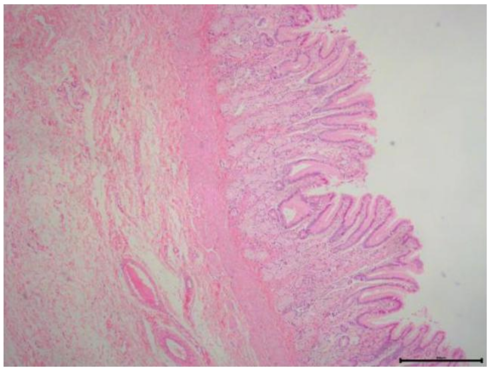

Canine stomach tissue section

(4×，H.E.)

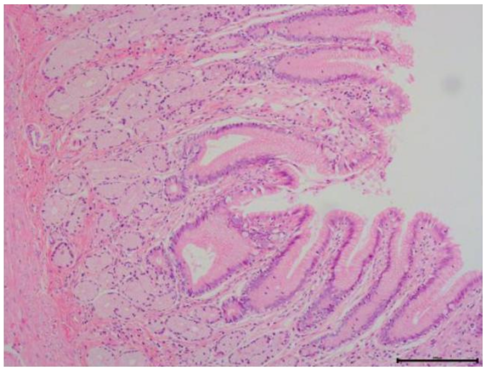

Canine stomach tissue section

(10×，H.E.)

"*3": pancreatic edema

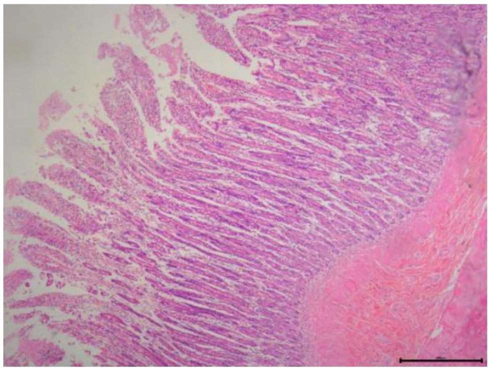

Canine duodenum tissue section

(4×，H.E.)

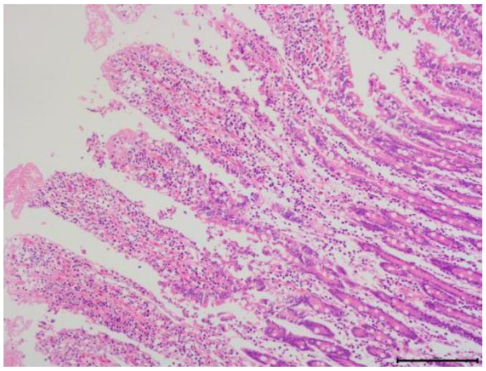

Canine duodenum tissue section

(10×，H.E.)

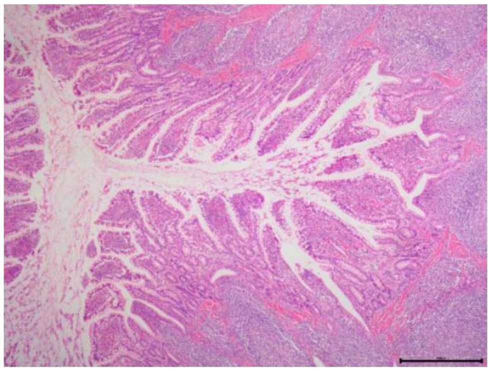

Canine ileum tissue section

(10×，H.E.)

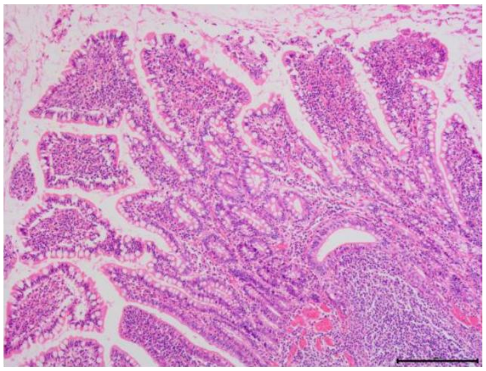

Canine ileum tissue section

(10×，H.E.)

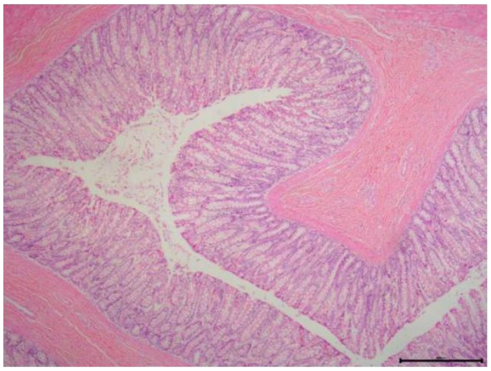

Canine rectum tissue section

(4×，H.E.)

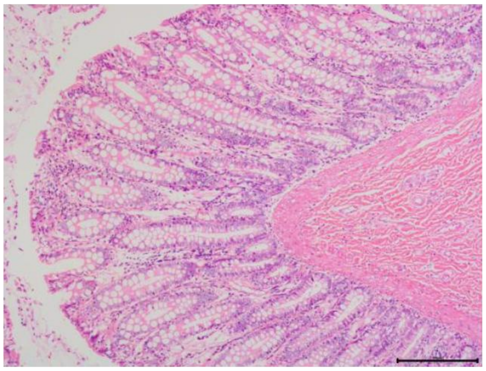

Canine rectum tissue section

(10×，H.E.)
